# Geotemporospatial and causal inference epidemiological analysis of US survey and overview of cannabis, cannabidiol and cannabinoid genotoxicity in relation to congenital anomalies 2001–2015

**DOI:** 10.1186/s12887-021-02996-3

**Published:** 2022-01-19

**Authors:** Albert Stuart Reece, Gary Kenneth Hulse

**Affiliations:** 1 Division of Psychiatry, University of Western Australia, Crawley, WA 6009 Australia; 2 School of Medical and Health Sciences, Edith Cowan University, Joondalup, WA 6027 Australia

**Keywords:** Cannabis, Cannabinoid, Δ9-tetrahydrocannabinol, Cannabigerol, Cannabidiol, Mechanisms, Congenital anomalies, Teratogenesis, Genotoxicity, Epigenotoxicity, Limb reduction deficiencies, Leg reduction deficiencies, Chromosomal toxicity, Multigenerational genotoxicity, Transgenerational teratogenicity

## Abstract

**Background:**

Cannabinoids including cannabidiol have recognized genotoxic activities but their significance has not been studied broadly epidemiologically across the teratological spectrum. We examined these issues including contextual space-time relationships and formal causal inferential analysis in USA.

**Methods:**

State congenital anomaly (CA) rate (CAR) data was taken from the annual reports of the National Birth Defects Prevention Network 2001–2005 to 2011–2015. Substance abuse rates were from the National Survey of Drug Use and Health a nationally representative longitudinal survey of the non-institutionalized US population with 74.1% response rate. Drugs examined were cigarettes, monthly and binge alcohol, monthly cannabis and analgesic and cocaine abuse. Early termination of pregnancy for abortion (ETOPFA) rates were taken from the published literature. Cannabinoid concentrations were from Drug Enforcement Agency. Ethnicity and income data were from the US Census Bureau. Inverse probability weighted (IPW) regressions and geotemporospatial regressions conducted for selected CAs.

**Results:**

Data on 18,328,529 births from an aggregated population of 2,377,483,589 for mid-year analyses 2005–2013 comprehending 12,611 CARs for 62 CAs was assembled and ETOPFA-corrected (ETOPFACAR) where appropriate. E-Values for ETOPFACARs by substance trends were elevated for THC (40 CAs), cannabis (35 CAs), tobacco (11 CAs), cannabidiol (8 CAs), monthly alcohol (5 CAs) and binge alcohol (2 CAs) with minimum E-Values descending from 16.55, 1.55x10^7^, 555.10, 7.53x10^19^, 9.30 and 32.98. Cardiovascular, gastrointestinal, chromosomal, limb reductions, urinary, face and body wall CAs particularly affected. Highest v. lowest substance use quintile CAR prevalence ratios 2.84 (95%C.I. 2.44, 3.31), 4.85 (4.08, 5.77) and 1.92 (1.63, 2.27) and attributable fraction in exposed 0.28 (0.27, 0.28), 0.57 (0.51, 0.62) and 0.47 (0.38, 0.55) for tobacco, cannabis and cannabidiol. Small intestinal stenosis or atresia and obstructive genitourinary defect were studied in detail in lagged IPW pseudo-randomized causal regressions and spatiotemporal models confirmed the causal role of cannabinoids. Spatiotemporal predictive modelling demonstrated strongly sigmoidal non-linear cannabidiol dose-response power-function relationships (*P* = 2.83x10^−60^ and 1.61x10^−71^ respectively).

**Conclusions:**

Data implicate cannabinoids including cannabidiol in a diverse spectrum of heritable CAs. Sigmoidal non-linear dose-response relationships are of grave concern.

These transgenerational genotoxic, epigenotoxic, chromosomal-toxic putatively causal teratogenic effects strongly indicate tight restrictions on community cannabinoid penetration.

**Supplementary Information:**

The online version contains supplementary material available at 10.1186/s12887-021-02996-3.

## Background

Both “Epidiolex” (cannabidiol) registered in the USA by the Food and Drug Administration (FDA) and Sativex (Δ9-tetrahydrocannabinol (THC) - cannabidiol) registered by the Medicines and Healthcare Products Regulatory Authority (MHRA) of the United Kingdom carry strong warnings on their Product Information and Prescribers Information leaflets against their use in pregnancy and breast feeding which is the standard warning for genotoxic effects which routinely accompanies medicines including cytotoxic and cancer agents [1, 2]. Similar warnings occur on the labelling of “Hemp Oil” which is made freely accessible to the Australian public on supermarket shelves. Such overt warnings relating to acknowledged genotoxicity by the distributors and marketers of cannabinoids, and mandated warnings required by official drug regulators on both sides of the Atlantic directly imply that the genotoxicity of these agents is acknowledged in laboratory and preclinical studies and is in truth an established fact of science.

Paradoxically what might be termed the “standard” or “establishment” view of the risks posed by the use of cannabinoid products in pregnancy is relatively benign. Major authorities and several smaller convenience sample series claim that the use of cannabis in pregnancy is associated with increased prematurity, smaller head circumference, increased small for gestational age, low birth weight and relative infertility in male and female users [3–5]. This view which enjoys widespread currency in the medical profession, is clearly at odds with the official governmental view endorsed in the requirements on registered product information for the medical profession and consumers, but is nevertheless typical of the community-wide confusion relating to much of the information on cannabis and cannabinoids.

A broader and more concerning view on cannabinoid teratogenicity is expressed by other authorities including the Centres for Disease Control (CDC) Atlanta, Georgia, the American Heart Association (AHA) and the American Academy of Paediatrics (AAP) who have together warned of increased rates of six birth defects after prenatal cannabis exposure including ventricular septal defect, Epsteins anomaly, gastroschisis, diaphragmatic hernia, oesophageal atresia with or without tracheoesophageal fistula and anencephalus [6–8]. The American College of Obstetricians and Gynaecologists (ACOG) strongly warn against the use of cannabis products in pregnancy [9]. Longitudinal studies of neurological and psychomotor development in prenatally exposed children conducted in Pittsburgh, Toronto and Netherlands uniformly indicate worrying levels of autism-like and ADHD-like features with altered neurological development and impairments of emotional development, motor tone and fine motor skills and cortical executive and visuospatial processing [10].

The most useful experimental animal models in which to study the effects of prenatal drug exposure are New Zealand white rabbits and hamsters. Classical studies from 1969 performed in rodents and hamsters showed a variety of defects including limb reduction, exencephaly, spina bifida, omphalocele, multiple malformations and myelocoele [11, 12]. As was noted at the time “this is a formidable list” [12]. However clinical confirmation of such a concerning and wide-ranging spectrum of congenital anomalies was mostly lacking. In 2007 a novel report from Hawaii listed 21 birth defects as being elevated after prenatal cannabis exposure, particularly affecting the cardiovascular, gastrointestinal, urinary and chromosomal systems and including arm defects, syndactyly and polydactyly however this study remained very much an exception and outlier for many years [13].

In an historical case series of illicit poly-drugs users from Washington DC 148 pregnancies amongst 140 women produced 12 embryos or infants with major congenital abnormalities, 43% had spontaneous first trimester abortions and four of eight serial pregnancies produced infants or embryos with major abnormalities [14]. The major congenital anomaly rate was a calculated by the authors at 96/1000 live births or 16 times the then control rate in USA in 1972 [14]. The usually quoted rate for spontaneous abortions at that time in USA was up to 20%. Of the eight infants whose major congenital anomaly was listed six had neural tube closure defects (meningomyelocele, myelocele, spina bifida or hydrocephalus), one had a cardiovascular defect (Tetralogy of Fallot), one had neuroblastoma and one had limb abnormalities (absent feet, absent finger and absent phalanges from fingers). All patients smoked cannabis [14, 15].

A report on atrial septal defect secundum type from the CDC database showing much higher rates and a steep acceleration of the rate of increase of atrial septal defect in high cannabis use states in the USA in recent years appeared which carried two major corollaries [16]. Firstly it implied that the list of cardiovascular anomalies jointly proposed by the AAP and AHA was incomplete. Secondly it implied that our knowledge of the subject of clinical cannabinoid teratogenesis including the list of cannabis-related congenital anomalies was similarly incomplete.

The concerning Hawaiian study has since been supported by studies from other locations. Confirmation of the experimentally identified spina bifida and encephalocele findings recently came from an analysis of Canadian data [17]. Indeed total congenital anomalies, particularly including cardiovascular defects and chromosomal anomalies were recently noted to be three times higher in the northern Territories of Canada which traditionally smoke two to three times as much cannabis as Canadians living in the south [18]. An Australian report showed that 18 congenital defects were higher in high cannabis using parts of Northern New South Wales [19]. Colorado was noted to have a 29% jump in the expected rate of total birth defects across the period of cannabis legalization 2000–2013 and included particularly cardiovascular, central nervous system, genitourinary, musculoskeletal and chromosomal CAs [20].

Cannabinoids including cannabidiol have been implicated in direct damage by oxidation to DNA bases which is a major genotoxic and mutagenic lesion [21]. They have long been known to be toxic to chromosomes which are the natural way in which DNA is packaged inside the cell nucleus [22]. It was shown long ago that cannabinoids reduce the synthesis of the major molecules of biology DNA, RNA proteins and histones [23–34]. Such gross level changes necessarily impact the genomic code. Translated into a twenty-first century understanding this would imply major interference in the epigenetic code where genome accessibility, controlled by histone modifications, the formation of euchromatin and the assembly of topologically organized transcriptionally active domains (the chromosomal “A compartment”) within the nucleus constitutes a major portion of normal gene regulation, cell function and indeed epigenetic cell specification and lineage determination [35]. And it has been well established that cannabinoids carry a heavy epigenetic footprint which is inheritable for several subsequent generations [35–41].

As was recently observed chromosomal toxicity, genotoxic and epigenotoxic lesions can reasonably be expected to manifest in congenital anomaly profiles and patterns of cancerogenesis [42]. What is clearly lacking in the literature is a genotoxic survey of a national teratological database to study the issue of patterns of teratogenesis as they relate to substance exposure. The application of the formal techniques of geospatial analysis and causal inferential analysis to the whole database tracked by CDC of 62 birth defects is a massive task which can only be commenced in this forum. It is therefore our purpose in the present paper to present an overview and introduction to this topic with a few teratological case examples to illustrate the way in which such studies can be extended and the power of these analytical techniques. Formal treatment of the whole field must be left for another occasion. Since the required teratological and substance exposure and related data is available for USA that nation has been chosen for the present investigation.

As has been pointedly observed it is vitally important in any review of teratological epidemiology to consider the impact of early termination of pregnancy for anomalies (ETOPFA) [43, 44]. Our study provides estimates of these ETOPFA practices which are used to complete applicable datasets for affected congenital anomalies (CAs).

Given the rapid increase in the penetration of cannabis and cannabinoids into modern American society, all studies related to cannabinoid teratogenesis and cannabinoid genotoxicity must be regarded as urgent and of high priority in the national research agenda.

A related concern is the potential for cannabinoids to enter the food chain. Cases of babies born without limbs have been noted in France and Germany where cannabis has become widely available [45–50] however this has not been seen in nearby Switzerland where its entry into the food chain is not permitted. Rapid introduction of cannabis into Colorado recently was associated with a 29% jump in total congenital anomalies [20] and Kentucky saw a massive and sharp spike in the incidence of atrial septal defect in recent years as cannabis has increasingly replaced tobacco as a major cash crop [16].

Not since Distillers unleashed thalidomide on the global market in 1957 has an agent which is known to be genotoxic been aggressively marketed for commercial reasons [51]. Of note the thalidomide debacle was avoided in the USA primarily because of genotoxic concerns [52, 53]. This international tragedy of recent history is also the foundational reason for the development of the modern drug regulatory scheme in many nations [53].

Aside from the fact of cannabis mutagenicity and genotoxicity itself one of the aspects of this subject which we find of most concern is the clear replication in many predictive geotemporospatial models of a sigmoidal relationship between cannabidiol and cannabinoid exposure and teratogenic outcomes for many congenital anomalies which is clearly highly reminiscent of the exponential dose-response relationships observed in numerous in vitro studies of cannabinoid genotoxicity and mitochondriopathy-epigenotoxicity [24, 26, 31, 54–65]. It is the non-linear power function of dose-response between increased cannabinoid exposure and teratological outcomes which must be of particular concern to any community moving into a higher cannabinoid exposure zone. Equally of concern an exponential relationship was observed in both actual and predicted modelled trend studies of the relationship between cannabinoid exposure and US autism incidence [66]. Taken together such findings imply exponentiation both of major neurotoxic and major genotoxic developmental outcomes.

It is self-evident that with the endocannabinoids playing critical roles in many body systems drugs modulating the endocannabinoid system will increasingly enter the international therapeutic marketplace in the coming years. We also feel that in order to assist cannabinoid therapeutics to find their appropriate niche in the global market that a proper understanding and appreciation of their long term neurotoxic and genotoxic activities is an absolute requirement both for regulators and for the public at large so that intergenerational community safety continues to be prioritized as a central and principal concern.

The overall purpose of the present analysis was to investigate substance and particularly cannabinoid exposure as a putative environmental risk factor for the observed spectrum of congenital anomalies. This was done directly using ecological USA data in bivariate analysis of continuous covariates. Key epidemiological parameters of public health interest such as the prevalence ratio, the aetiological fraction in the exposed and the population attributable risk were calculated from an analysis of categorized data. Detailed multivariable regression was undertaken using inverse probability weighted mixed effects, robust and panel regression for two selected CAs and spatiotemporal regression was also conducted for these CAs. Extensive use of the formal techniques of causal inference namely E-Values and inverse probability weighting was engaged to correct for the ecological fallacy and convert data into a pseudo-randomized quasi-experimental design. Finally predictive mathematical modelling was conducted to study overall trends of selected CAs as a function of cannabinoid exposure.

The minimum E-Value indicates the minimum strength of association required of some extraneous confounder covariate with both the outcome of interest and the exposure of concern to explain an observed assocation [67–69]. It plays a central role in formal epidemiological assignment of causal relationships.

An overview and survey of a geospatial consideration of the field of genotoxicity manifested as cancerogenesis is the subject of a series of companion papers.

## Methods

### Data

Rates of birth defects were taken from the annual reports of the National Birth Defects Prevention Network (NBDPN) 2001–2005 to 2011–2015 which is coordinated from the Centres for Disease Control (CDC), Atlanta, Georgia. For the purposes of conducting the analysis the nominal year of the report was taken as the temporal midpoint of the year of the report. Hence for the most recent report we used which was 2011–2015 [70] the nominal year for analysis was 2013. We analyzed all the major CAs collected long term by NBDPN across this period totally 62 CAs. This was joined with annual USA state based drug use cross-tabulation data from the National Survey of Drug Use and Health (NSDUH) Substance Use and Mental Health Data Archive (SAMHDA) Restricted-Use Data Analysis System (RDAS) maintained by the Substance Abuse and Mental Health Services Administration (SAMHSA) [71]. The drugs of interest were last month cigarette use, last month alcohol use, last year binge alcohol use, last year non-medical use of opioid analgesia (Analgesics), last month use of cannabis and last year use of cocaine. Substance exposure was also considered as a categorical variable. This was facilitated by establishing substance exposure quintiles for each year with the first quintile representing the lowest exposure and the fifth quintile the highest exposure. The cannabinoid concentration in Federal cannabis seizures was taken from published reports of the Drug Enforcement agency [72–74]. Estimates of state level cannabinoid exposure was derived by multiplying the last month cannabis use rates by the Federal cannabinoid concentration. Quintiles for cannabinoid exposure were calculated across the whole period as a single group.

Some CAs and those particularly affecting chromosomal defects are heavily impacted by ETOPFA practice. The final ETOPFA rate by anomaly was arrived at as a composite synthesis of several published ETOPFA rates [75–82]. Moreover, as defined in at least one longitudinal annual time series of ETOPFA rates it seems highly likely that the ETOPFA rate has been incrementally increasing over time [83]. In the longitudinal time series the ETOPFA rate for Downs syndrome rose from low levels in 1980 to 70% in 2014. This approximately linear rate of rise has been projected across all CAs according to the following formula:

$$ETOPFA\_ Rate= Reported\_ Rate/\left( 1-\left( Composite\ast FMaxTR\right)\right)$$

where ETOPFA_Rate represents the adjusted CA rate, the Reported_Rate is the gazetted rate reported by NBDPN, the Composite rate is the composite rate derived from literature review shown in Table 1 and the FMaxTR is the Fraction of the Maximal Termination Rate in the year in question given in Supplementary Table 1 which is a tabular representation of graphical data taken from the only longitudinal series of ETOPFAs in the world we were able to identify [83].

**Table 1:** Regression Slopes for ETOPFA-Corrected Congenital Anomaly Rates by Cigarette Exposure

		Parameters				Model				E-Values	
Congenital Anomaly	Term	Estimate	Std.Error	t-Value	P_Value	Adj.R.Squared	S.D.	t-Statistic	***P***-Value	E-Value - Point	E-Value - Lower
Atrial septal defect	Cigarettes	461.4473	80.9277	5.7020	2.98E-08	0.0999	49.0262	32.5125	2.98E-08	10,490.78	555.10
Common truncus (truncus arteriosus)	Cigarettes	10.0328	1.8075	5.5506	6.33E-08	0.0912	1.0985	30.8095	6.33E-08	8137.59	434.55
Pyloric stenosis	Cigarettes	108.3707	29.4229	3.6832	0.0004	0.1025	9.7747	13.5660	0.0004	48,155.03	226.33
Tetralogy of Fallot	Cigarettes	8.6017	2.5730	3.3431	0.0009	0.0319	1.5895	11.1763	0.0009	274.78	14.91
Diaphragmatic hernia	Cigarettes	6.1187	1.9038	3.2139	0.0015	0.0306	1.1633	10.3291	0.0015	239.26	12.50
Double outlet right ventricle	Cigarettes	5.8943	2.1948	2.6856	0.0080	0.0369	1.0114	7.2122	0.0080	401.58	7.91
Rectal and large intestinal atresia/stenosis	Cigarettes	5.9460	2.2635	2.6269	0.0091	0.0198	1.3888	6.9008	0.0091	97.91	4.85
Dextro-transposition of great arteries (d-TGA)	Cigarettes	5.1147	2.1421	2.3877	0.0180	0.0260	1.0324	5.7011	0.0180	181.05	3.95
Transposition of great arteries	Cigarettes	5.8459	2.5671	2.2773	0.0235	0.0138	1.5155	5.1859	0.0235	66.41	2.67
Hypoplastic left heart syndrome	Cigarettes	4.7634	2.3941	1.9897	0.0475	0.0095	1.4718	3.9588	0.0475	37.52	1.28
Holoprosencephaly	Cigarettes	44.6731	22.6580	1.9716	0.0506	0.0195	10.1998	3.8873	0.0506	107.14	1.21
Cloacal exstrophy	Cigarettes	13.4134	7.0820	1.8940	0.0608	0.0220	2.7748	3.5873	0.0608	162.22	1.00
Ventricular septal defect	Cigarettes	64.3023	34.2374	1.8781	0.0614	0.0088	20.1650	3.5274	0.0614	35.91	1.00
Hydrocephalus without spina bifida	Cigarettes	21.5055	12.6853	1.6953	0.0926	0.0153	4.5872	2.8741	0.0926	142.01	1.00
Hypospadias	Cigarettes	58.5925	40.8897	1.4329	0.1530	0.0038	24.2028	2.0533	0.1530	17.59	1.00
Bladder exstrophy	Cigarettes	0.3683	0.2706	1.3607	0.1747	0.0031	0.1604	1.8515	0.1747	15.63	1.00
Biliary atresia	Cigarettes	0.8244	0.7004	1.1770	0.2402	0.0014	0.4223	1.3853	0.2402	11.30	1.00
Hirschsprung disease (congenital megacolon)	Cigarettes	5.2534	4.4871	1.1708	0.2441	0.0032	1.5328	1.3708	0.2441	44.74	1.00
Craniosynostosis	Cigarettes	13.2676	11.3680	1.1671	0.2462	0.0039	3.8587	1.3621	0.2462	45.19	1.00
Choanal atresia	Cigarettes	1.3216	1.1914	1.1093	0.2682	0.0008	0.7233	1.2307	0.2682	10.02	1.00
Amniotic Bands	Cigarettes	1.9776	1.8318	1.0796	0.2840	0.0023	0.5285	1.1656	0.2840	59.76	1.00
Cleft palate alone	Cigarettes	5.3064	4.9157	1.0795	0.2814	0.0007	2.7774	1.1653	0.2814	10.85	1.00
Ebstein anomaly	Cigarettes	0.7405	0.7584	0.9763	0.3297	−0.0002	0.4646	0.9532	0.3297	8.00	1.00
Reduction deformity, Lower limbs	Cigarettes	8.6524	9.1172	0.9490	0.3445	−0.0008	3.2313	0.9006	0.3445	22.36	1.00
Pulmonary valve atresia	Cigarettes	3.3612	3.8692	0.8687	0.3861	−0.0013	1.9006	0.7546	0.3861	9.47	1.00
Cleft lip with cleft palate	Cigarettes	3.2169	4.0414	0.7960	0.4271	−0.0020	1.8393	0.6336	0.4271	9.29	1.00
Gastroschisis	Cigarettes	2.1392	3.3067	0.6469	0.5182	−0.0021	1.9126	0.4185	0.5182	4.98	1.00
Clubfoot	Cigarettes	7.7021	14.8418	0.5189	0.6047	−0.0057	5.8097	0.2693	0.6047	6.14	1.00
Obstructive genitourinary defect	Cigarettes	19.2026	38.0533	0.5046	0.6148	−0.0066	12.9550	0.2546	0.6148	7.17	1.00
Coarctation of the aorta	Cigarettes	3.2398	6.6943	0.4840	0.6288	−0.0025	4.1228	0.2342	0.6288	3.51	1.00
Aniridia	Cigarettes	0.5344	1.4146	0.3778	0.7063	−0.0082	0.4681	0.1427	0.7063	5.10	1.00
Anophthalmia/microphthalmia	Cigarettes	1.5564	6.3984	0.2432	0.8080	−0.0034	3.8287	0.0592	0.8080	2.25	1.00
Epispadias	Cigarettes	0.2592	2.5368	0.1022	0.9189	−0.0121	0.7690	0.0104	0.9189	2.06	1.00
Interrupted aortic arch	Cigarettes	0.1509	2.0077	0.0751	0.9402	−0.0072	0.8982	0.0056	0.9402	1.60	1.00
Microcephalus	Cigarettes	0.3421	12.8915	0.0265	0.9789	−0.0084	4.5413	0.0007	0.9789	1.35	1.00
Encephalocele	Cigarettes	−0.0017	2.1734	−0.0008	0.9994	−0.0034	1.3370	0.0000	0.9994	1.04	NA
Congenital posterior urethral valves	Cigarettes	−0.5966	6.8233	−0.0874	0.9305	−0.0069	2.9831	0.0076	0.9305	1.69	NA
Single ventricle	Cigarettes	−0.3417	2.1972	−0.1555	0.8766	−0.0065	0.9898	0.0242	0.8766	2.08	NA
Congenital hip dislocation	Cigarettes	−6.9146	20.8003	−0.3324	0.7402	−0.0086	5.9638	0.1105	0.7402	5.19	NA
Renal agenesis/hypoplasia	Cigarettes	−2.2676	5.0062	−0.4530	0.6509	−0.0027	3.0895	0.2052	0.6509	3.31	NA
Esophageal atresia/tracheoesophageal fistula	Cigarettes	−0.6793	1.1994	−0.5664	0.5716	−0.0023	0.7428	0.3208	0.5716	4.03	NA
Small intestinal atresia/stenosis	Cigarettes	−1.8286	2.8694	−0.6373	0.5250	−0.0042	1.2732	0.4061	0.5250	6.85	NA
Pulmonary valve atresia and stenosis	Cigarettes	−42.7272	64.7992	−0.6594	0.5102	−0.0019	38.9612	0.4348	0.5102	4.87	NA
Spina bifida without anencephalus	Cigarettes	−4.8101	6.5013	−0.7399	0.4599	−0.0014	4.0680	0.5474	0.4599	5.31	NA
Atrioventricular septal defect	Cigarettes	−3.1546	4.0985	−0.7697	0.4422	−0.0015	2.4370	0.5924	0.4422	5.95	NA
Anencephalus	Cigarettes	−9.9229	12.0861	−0.8210	0.4123	−0.0010	7.5960	0.6741	0.4123	6.02	NA
Cleft lip with and without cleft palate	Cigarettes	−7.2523	8.1749	−0.8871	0.3767	−0.0016	3.0661	0.7870	0.3767	16.70	NA
Omphalocele	Cigarettes	−6.3434	6.2594	−1.0134	0.3118	0.0001	3.5702	1.0270	0.3118	9.55	NA
Patent ductus arteriosus	Cigarettes	−134.9204	93.6508	−1.4407	0.1527	0.0103	26.7177	2.0755	0.1527	197.55	NA
Cleft lip alone	Cigarettes	−7.6263	5.0141	−1.5210	0.1300	0.0072	2.3847	2.3134	0.1300	36.22	NA
Aortic valve stenosis	Cigarettes	−5.5657	3.5177	−1.5822	0.1147	0.0052	2.1283	2.5034	0.1147	21.09	NA
Limb deficiencies (reduction defects)	Cigarettes	−9.2468	5.6656	−1.6321	0.1044	0.0093	2.6416	2.6637	0.1044	47.85	NA
Congenital cataract	Cigarettes	−3.1133	1.7342	−1.7952	0.0737	0.0077	1.0449	3.2228	0.0737	29.59	NA
Reduction deformity, Upper limbs	Cigarettes	−9.9676	4.2945	−2.3210	0.0219	0.0342	1.5208	5.3870	0.0219	778.14	NA
Total anomalous pulmonary venous connection	Cigarettes	−2.9518	0.9933	−2.9718	0.0034	0.0421	0.4914	8.8318	0.0034	472.81	NA
Tricuspid valve atresia and stenosis	Cigarettes	−13.5992	4.5124	−3.0137	0.0028	0.0268	2.7617	9.0825	0.0028	176.14	NA
Deletion 22q11.2	Cigarettes	−4.0755	1.2068	−3.3771	0.0010	0.0817	0.5118	11.4051	0.0010	2803.97	NA
Turner syndrome	Cigarettes	−67.5119	15.2076	−4.4394	0.0000	0.1217	6.7057	19.7079	0.0000	19,050.01	NA
Trisomy 13	Cigarettes	−47.5542	8.4152	−5.6510	0.0000	0.0943	5.1389	31.9335	0.0000	9081.76	NA
Trisomy 18	Cigarettes	−102.6539	15.9192	−6.4485	0.0000	0.1174	9.7711	41.5825	0.0000	28,380.44	NA
Trisomy 21 (Down syndrome)	Cigarettes	−145.2252	19.7758	−7.3436	0.0000	0.1423	12.4068	53.9284	0.0000	84,541.57	NA
Anotia/microtia	Cigarettes	−47.4905	6.3089	−7.5275	0.0000	0.1587	3.8479	56.6635	0.0000	150,869.58	NA

Median household income and ethnicity data by state and year was sourced using tidycensus package [84] in R directly from the US Census bureau including linear interpolation for missing year data. The main ethnicities which were tracked included: Native Hawaiian / Pacific Islander (NHPI), American Indian / Alaska Native (AIAN), Asian-American, Hispanic-American, African-American and Caucasian-American. Cannabinoid concentration data in USA at the Federal level was taken from published reports of the US Drug Enforcement Agency (DEA) [72–74]. The five cannabinoids of interest were Δ9-tetrahydrocannabinol (THC), cannabidiol (CBD), cannabigerol (CBG), cannabinol (CBN), and cannabichromene (CBC). Federal cannabinoid concentration was multiplied by state level cannabis use to compute an estimate of cannabinoid exposure in each state.

Further technical details relating to statistical methodology are provided in an online Statistical Appendix.

### Data availability

Data, including R-code, spatial weights, ipw weights and main source datasets has been made freely available through the Mendeley Data repository online and can be accessed at 10.17632/w6ks529sxd.1 .

### Ethics

The University of Western Australia Human Research Ethics Committee granted ethical approval for this study on 7th January 2020 RA/4/20/7724.

## Results

This section is set out in three sections. First we examine bivariate continuous associations. We then calculate key epidemiological parameters of interest from categorization of key exposure variables. We then demonstrate how inverse probability weighting can be employed in multivariable regression models and also use spatiotemporal models to investigate causal relationships formally and in a space-time context as an analytical pathway proof of concept for subsequent detailed studies across all congenital anomalies.

18,328,529 births occurred in USA in the eight nominal years 2005–2013. 2008 was omitted as CA data was not available for that year. The cumulative aggregated population of the USA for these eight years year-on-year was 2,377,483,589. 12,611 birth defect rates relating to 62 birth defects in the 50 states of the USA were extracted from the published reports of the National Birth Defects Prevention Network which is coordinated by the CDC. The defects of interest are listed in Supplementary Table 1. The period of interest was 2005–2013 as that period could be related to drug and substance exposure data from the NSDUH from SAMHSA. Since NBDPN reports are issued for quinquennia this report comprehends the NBDPN reports from 2003–2007 to 2011–2015.

It is well known that several congenital anomalies are actively sought out by active antenatal screening programs. Some of these are subject to indications for early therapeutic termination of pregnancy for anomaly (ETOPFA). In considering the likely rate of congenital anomalies it is important to take this effect into consideration. Supplementary Table 1 also lists the ETOPFA rates from various published series [72–74]. Series were selected for their breadth of coverage of multiple congenital anomalies. The right hand column lists the ETOPFA rates applied in the present work which were a composite of these series. This estimate of the ETOPFA-corrected rate was a dependent variable of interest in some of the present analyses. Supplementary Table 2 shows the time-dependent progression of the only longitudinal series of ETOPFA’s we were able to identify which was the Down Syndrome ETOPFA rate in Western Australia [83].

### Continuous bivariate exposure survey

**Fig. 1:**
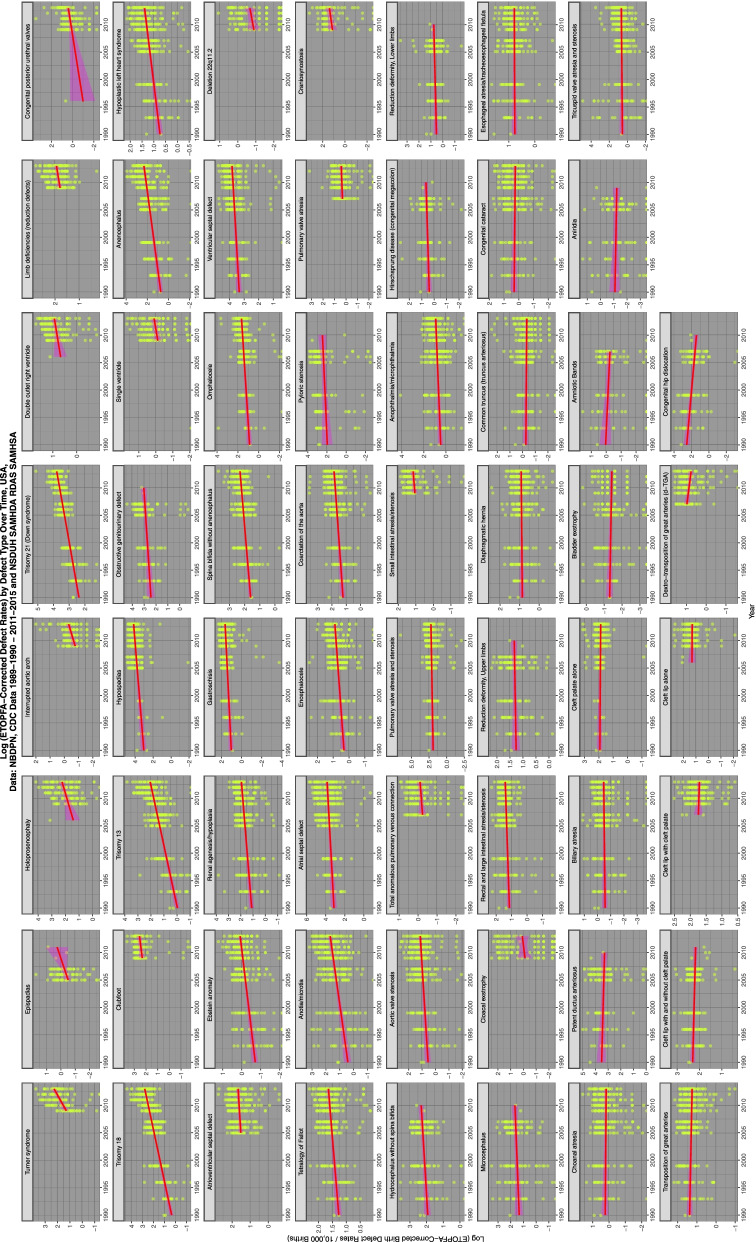
Time trends of selected congenital anomaly incidence rates

Figure 1 shows the time dependent trajectories of these various CAs corrected for estimates of ETOPFA.

**Fig. 2:**
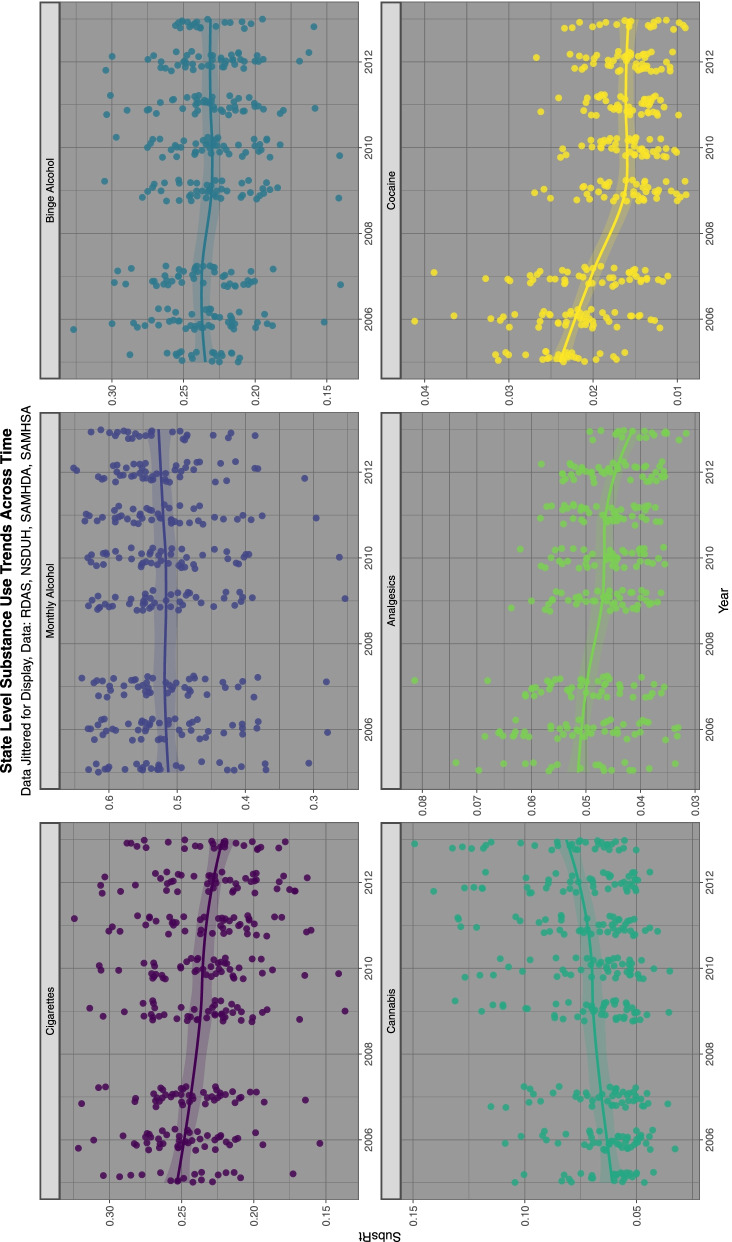
Trends over time of various selected substances, data from National Survey of Drug Use and Health

Figure 2 shows the substance exposure trends over this time period. Data was taken from the nationally representative annual SAMHSA NSDUH which reports a 74.1% response rate [85].

**Fig. 3:**
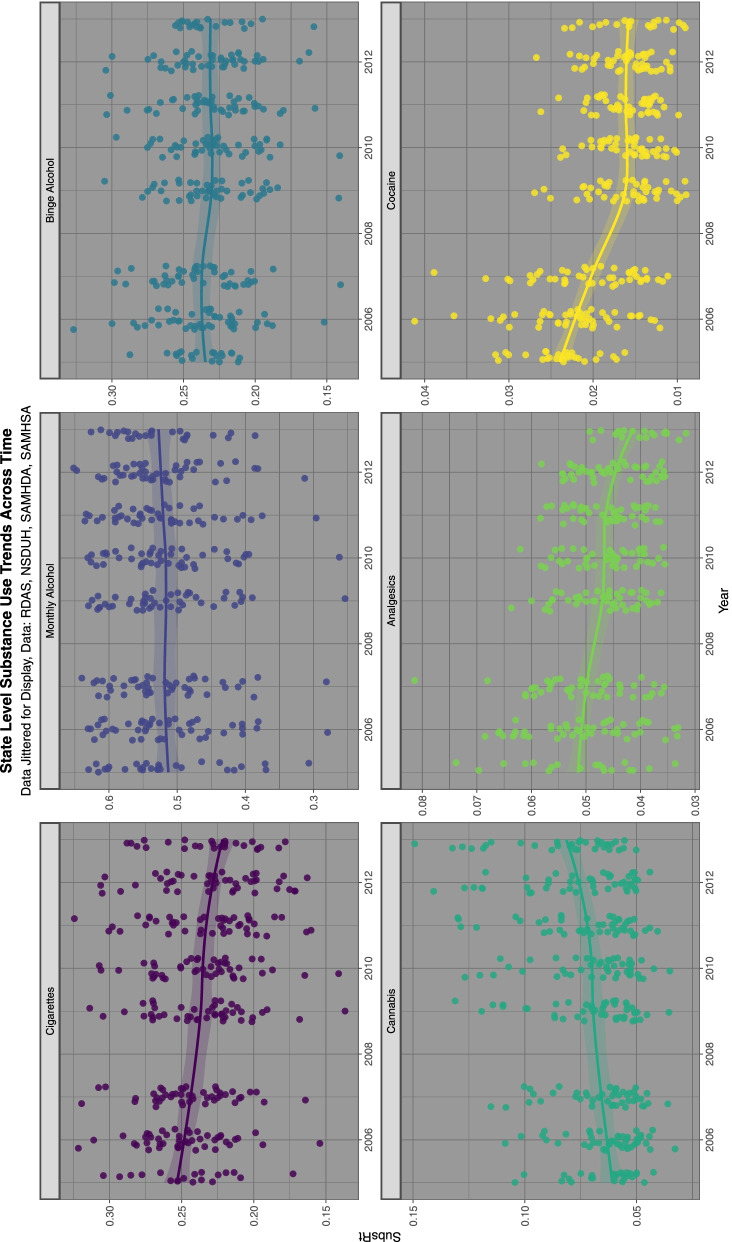
Trends over time of various selected estimates of cannabinoid exposure, data from National Survey of Drug Use and Health and Drug Enforcement Agency

Figure 3 shows the annual estimated cannabinoid exposure for state level data estimated from Federal data from the DEA relating to cannabinoid concentrations in drug seizures and the state level last month cannabis consumption. Rising trends are noted for all cannabinoids except cannabidiol which is declining.

**Fig. 4:**
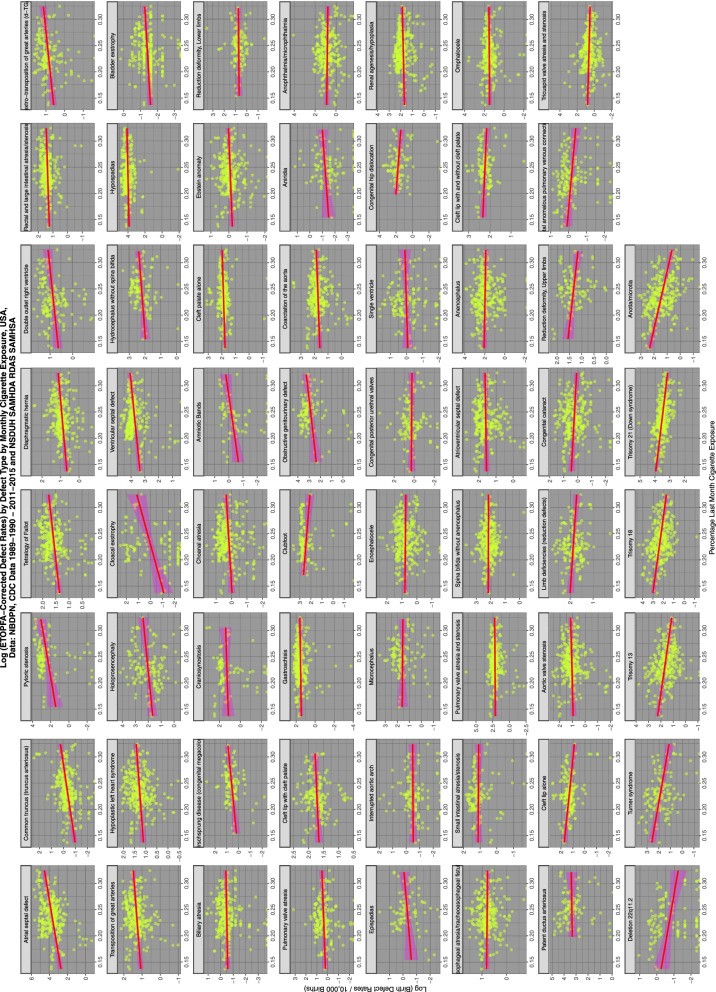
Trends of various congenital anomaly incidence rates in relationship to tobacco exposure

Figure 4 shows the relationship of the various ETOPFA-corrected CA rates (ETOPFACAR) to tobacco exposure. As is expected many show a rising and positive relationship.

Supplementary Fig. 1 shows the relationship of the ETOPFACAR estimates to binge alcohol exposure. Mostly weak or negative relationships are demonstrated.

Supplementary Fig. 2 shows the relationship of the ETOPFACARs to last month alcohol use. Similar appearances are seen.

**Fig. 5:**
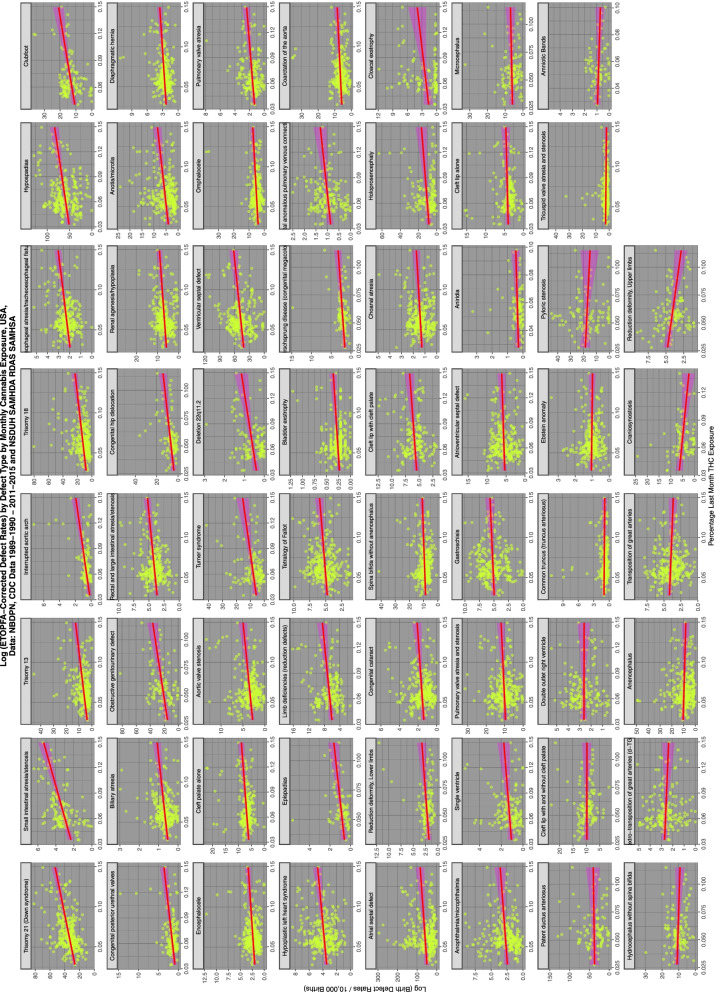
Trends of various congenital anomaly incidence rates in relationship to cannabis exposure

Moving to Fig. 5 and considering the relationship of ETOPFACARs to cannabis exposure the pattern changes dramatically from weak associations to many clearly strongly positive and apparently highly significant associations.

**Fig. 6:**
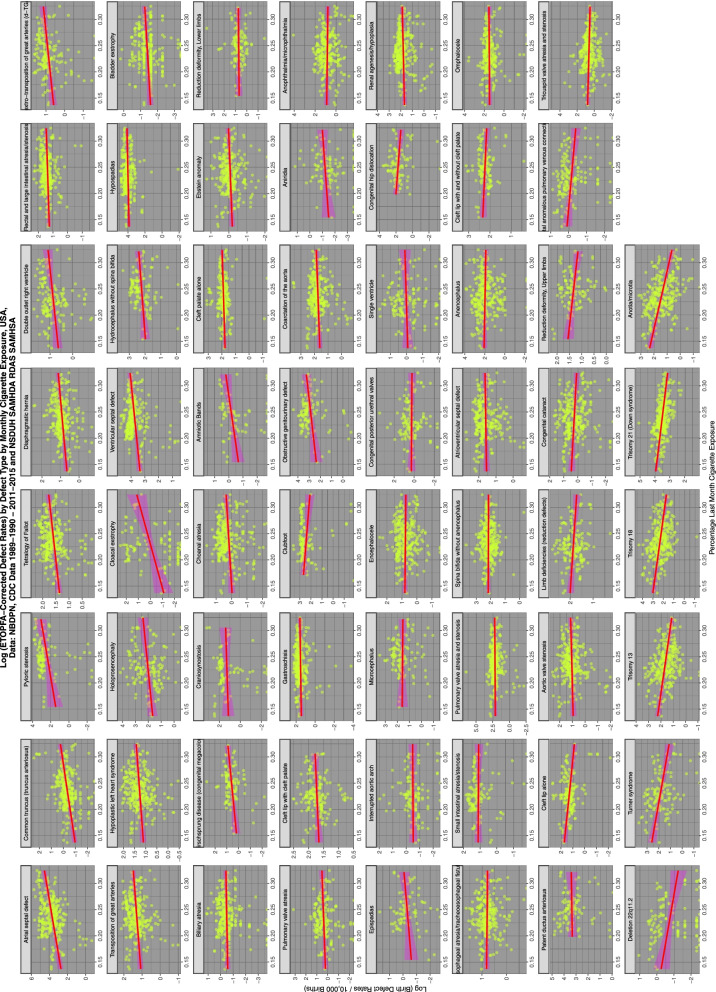
Trends of various congenital anomaly incidence rates in relationship to THC exposure

Figure 6 shows the relationship of the ETOPFACAR to THC exposure. Many of these relationships are clearly positive and highly significant.

**Fig. 7:**
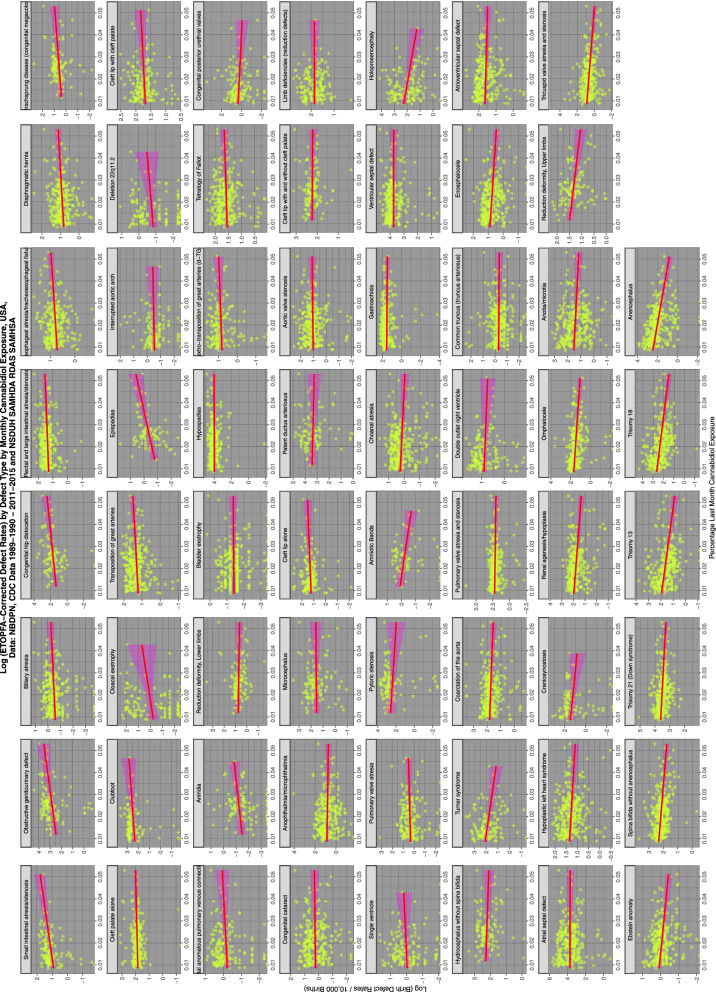
Trends of various congenital anomaly incidence rates in relationship to cannabidiol exposure

Figure 7 shows the relationship of the ETOPFACARs to state level estimated cannabidiol exposure. Some relationships appear to be positive, particularly in the top line of CAs.

Supplementary Table 3 provides details of the slopes of the ETOPFACARs over time. The table was produced using the purrr-broom package combination in R using the nest-map-unnest workflow whereby multiple linear models can be processed simultaneously for each CA. The table lists the model β-estimates, the t-values and various model statistics. Lastly the table lists the point estimates of the E-Values for these regression lines together with the 95% lower bound of the E-Value.

Table 1 performs a similar function for tobacco exposure. One notes that in this Table 12 ETOPFACARs have minimum E-Values greater than 1.00.

Supplementary Table 4 performs the same function for binge alcohol exposure. Only two ETOPFACARs have elevated minimum E-Values in this table which are cleft lip alone and epispadias.

Supplementary Table 5 performs the same function for last month alcohol exposure. Here six ETOPFACARs have elevated minimum E-Values.

Contrariwise Table 2, which illustrates the relationship of the ETOPFACARs with cannabis exposure, contrasts sharply with Table 5. In Table 6 one notes that 35 ETOPFACARs are shown to have elevated minimum E-Values. These pertain particularly to cardiovascular system (9 anomalies), urinary tract (6 anomalies), gastrointestinal tract (five anomalies), all five chromosomal anomalies, four musculoskeletal or limb development anomalies (club foot, congenital hip dislocation, limb reduction deficiencies and leg reduction deficiencies), two anomalies each of face and body wall, and one anomaly of brain development.

**Table 2:** Regression Slopes for ETOPFA-Corrected Congenital Anomaly Rates by Cannabis Exposure

		Parameters				Model				E-Values	
Congenital Anomaly	Term	Estimate	Std.Error	t-Value	P_Value	Adj.R.Squared	S.D.	t-Statistic	***P***-Value	E-Value - Point	E-Value - Lower
Small intestinal atresia/stenosis	Cannabis	26.5037	3.7660	7.0377	7.66E-11	0.2534	1.0978	49.5291	7.66E-11	6.95E+09	1.55E+07
Trisomy 21 (Down syndrome)	Cannabis	221.1194	25.4625	8.6841	2.03E-16	0.1891	10.2305	75.4141	2.03E-16	6.97E+08	8.30E+06
Interrupted aortic arch	Cannabis	15.4036	3.1814	4.8418	3.40E-06	0.1390	0.8305	23.4430	3.40E-06	4.28E+07	4.68E+04
Clubfoot	Cannabis	94.0309	21.7820	4.3169	3.16E-05	0.1211	5.4311	18.6357	3.16E-05	1.39E+07	1.10E+04
Congenital hip dislocation	Cannabis	115.8679	32.7515	3.5378	6.07E-04	0.0997	5.6345	12.5159	6.07E-04	2.68E+08	8.60E+03
Trisomy 13	Cannabis	75.1394	14.1320	5.3170	2.08E-07	0.0841	5.1679	28.2701	2.08E-07	1.11E+06	8.58E+03
Obstructive genitourinary defect	Cannabis	241.0897	66.6741	3.6159	4.49E-04	0.0958	12.2786	13.0750	4.49E-04	1.15E+08	7.30E+03
Congenital posterior urethral valves	Cannabis	23.9399	6.0470	3.9590	1.18E-04	0.0925	1.6001	15.6734	1.18E-04	1.64E+06	1.96E+03
Trisomy 18	Cannabis	126.9696	26.3799	4.8131	2.34E-06	0.0678	10.0424	23.1662	2.34E-06	1.99E+05	1.85E+03
Esophageal atresia/tracheoesophageal fistula	Cannabis	8.8449	1.8993	4.6570	4.83E-06	0.0645	0.7176	21.6880	4.83E-06	1.49E+05	1.34E+03
Hypospadias	Cannabis	277.1790	62.0518	4.4669	1.16E-05	0.0640	23.4595	19.9532	1.16E-05	9.34E+04	842.36
Biliary atresia	Cannabis	4.4970	1.2418	3.6215	0.0003	0.0418	0.4136	13.1152	3.48E-04	3.96E+04	188.70
Deletion 22q11.2	Cannabis	6.6430	2.1356	3.1106	0.0024	0.0690	0.5153	9.6756	0.0024	2.49E+05	155.04
Turner syndrome	Cannabis	85.6995	27.3283	3.1359	0.0021	0.0614	6.9321	9.8340	0.0021	1.54E+05	137.32
Rectal and large intestinal atresia/stenosis	Cannabis	13.0849	3.6262	3.6085	0.0004	0.0395	1.3748	13.0210	3.62E-04	1.16E+04	105.07
Epispadias	Cannabis	12.5446	4.8274	2.5986	0.0111	0.0648	0.7392	6.7528	0.0111	1.02E+07	90.57
Renal agenesis/hypoplasia	Cannabis	27.3954	8.0283	3.4124	7.34E-04	0.0346	3.0315	11.6442	0.0007	7.45E+03	66.37
Anotia/microtia	Cannabis	37.2830	10.9541	3.4036	7.57E-04	0.0346	4.1220	11.5843	0.0008	7.51E+03	65.76
Diaphragmatic hernia	Cannabis	10.2830	3.0660	3.3539	9.01E-04	0.0335	1.1615	11.2486	0.0009	6.31E+03	56.94
Cleft palate alone	Cannabis	24.1946	7.4701	3.2389	0.0014	0.0366	2.7271	10.4902	0.0014	6.42E+03	48.45
Encephalocele	Cannabis	11.3770	3.4999	3.2507	0.0013	0.0311	1.3138	10.5670	0.0013	5.29E+03	45.63
Aortic valve stenosis	Cannabis	17.8815	5.6987	3.1378	0.0019	0.0296	2.1020	9.8461	0.0019	4.60E+03	36.41
Ventricular septal defect	Cannabis	166.2143	53.4999	3.1068	0.0021	0.0296	19.9528	9.6523	0.0021	3.92E+03	32.64
Pulmonary valve atresia	Cannabis	9.4232	3.2900	2.8642	0.0047	0.0369	1.0048	8.2037	0.0047	1.02E+04	29.43
Omphalocele	Cannabis	28.8975	9.4470	3.0589	0.0025	0.0311	3.5144	9.3568	0.0025	3.55E+03	29.18
Hypoplastic left heart syndrome	Cannabis	10.7890	3.7873	2.8487	0.0047	0.0224	1.4621	8.1152	0.0047	1.65E+03	15.88
Hirschsprung disease (congenital megacolon)	Cannabis	19.3922	8.4341	2.2993	0.0233	0.0356	1.5076	5.2866	0.0233	2.42E+05	10.95
Limb deficiencies (reduction defects)	Cannabis	21.4215	8.5782	2.4972	0.0134	0.0287	2.6156	6.2360	0.0134	3.45E+03	9.53
Bladder exstrophy	Cannabis	1.0618	0.4420	2.4021	0.0170	0.0173	0.1593	5.7701	0.0170	860.98	5.62
Tetralogy of Fallot	Cannabis	9.9067	4.1188	2.4052	0.0168	0.0152	1.6031	5.7852	0.0168	553.33	5.16
Total anomalous pulmonary venous connection	Cannabis	3.9176	1.7901	2.1885	0.0299	0.0208	0.4968	4.7896	0.0299	2.61E+03	3.71
Reduction deformity, Lower limbs	Cannabis	16.8233	8.1886	2.0545	0.0420	0.0251	1.5723	4.2209	0.0420	3.39E+04	2.57
Coarctation of the aorta	Cannabis	22.5596	10.7794	2.0928	0.0372	0.0111	4.0947	4.3800	0.0372	300.37	2.12
Atrial septal defect	Cannabis	285.3616	136.7781	2.0863	0.0378	0.0117	51.3723	4.3527	0.0378	313.06	2.08
Congenital cataract	Cannabis	5.9492	2.9939	1.9871	0.0479	0.0102	1.0436	3.9486	0.0479	357.58	1.39
Spina bifida without anencephalus	Cannabis	19.7183	10.1652	1.9398	0.0533	0.0086	4.0477	3.7628	0.0533	167.88	1.00
Cleft lip with cleft palate	Cannabis	11.1868	5.7863	1.9333	0.0548	0.0149	1.8237	3.7377	0.0548	530.72	1.00
Choanal atresia	Cannabis	3.9066	2.0476	1.9078	0.0574	0.0090	0.7204	3.6399	0.0574	277.66	1.00
Holoprosencephaly	Cannabis	72.7261	39.0245	1.8636	0.0644	0.0168	10.2141	3.4730	0.0644	1.30E+03	1.00
Cloacal exstrophy	Cannabis	20.4977	11.5158	1.7800	0.0777	0.0185	2.7798	3.1683	0.0777	1.64E+03	1.00
Anophthalmia/microphthalmia	Cannabis	9.2992	5.2676	1.7654	0.0786	0.0075	1.7798	3.1165	0.0786	231.75	1.00
Single ventricle	Cannabis	6.1305	3.7353	1.6412	0.1029	0.0112	0.9811	2.6936	0.1029	589.19	1.00
Pulmonary valve atresia and stenosis	Cannabis	19.6240	13.4446	1.4596	0.1455	0.0038	5.0335	2.1305	0.1455	68.97	1.00
Gastroschisis	Cannabis	5.8564	4.9756	1.1770	0.2402	0.0014	1.9092	1.3854	0.2402	32.10	1.00
Atrioventricular septal defect	Cannabis	6.8595	6.7893	1.0103	0.3132	7.69E-05	2.4351	1.0208	0.3132	25.45	1.00
Aniridia	Cannabis	2.4802	3.2029	0.7744	0.4405	−0.0038	0.4671	0.5996	0.4405	250.47	1.00
Cleft lip alone	Cannabis	5.3804	7.5548	0.7122	0.4773	−0.0027	2.3966	0.5072	0.4773	14.91	1.00
Microcephalus	Cannabis	12.6431	24.1277	0.5240	0.6012	−0.0061	4.5361	0.2746	0.6012	24.76	1.00
Patent ductus arteriosus	Cannabis	39.3210	155.2633	0.2533	0.8006	−0.0092	26.9797	0.0641	0.8006	7.00	1.00
Cleft lip with and without cleft palate	Cannabis	−0.2957	15.7796	−0.0187	0.9851	−0.0077	3.0755	0.0004	0.9851	1.41	–
Double outlet right ventricle	Cannabis	−0.1429	3.4924	−0.0409	0.9674	−0.0062	1.0338	0.0017	0.9674	1.52	–
Common truncus (truncus arteriosus)	Cannabis	−1.2255	3.0794	−0.3980	0.6909	−0.0028	1.1539	0.1584	0.6909	4.70	–
Ebstein anomaly	Cannabis	−0.5654	1.2641	−0.4473	0.6550	−0.0028	0.4652	0.2001	0.6550	5.49	–
Pyloric stenosis	Cannabis	−41.8439	60.3518	−0.6933	0.4896	−0.0047	10.3424	0.4807	0.4896	78.93	–
Tricuspid valve atresia and stenosis	Cannabis	−5.6602	7.5460	−0.7501	0.4538	−0.0015	2.8017	0.5626	0.4538	12.05	–
Amniotic Bands	Cannabis	−3.2223	4.0536	−0.7949	0.4293	−0.0052	0.5304	0.6319	0.4293	502.82	–
Hydrocephalus without spina bifida	Cannabis	−20.4351	24.5694	−0.8317	0.4072	−0.0026	4.6285	0.6918	0.4072	110.65	–
Dextro-transposition of great arteries (d-TGA)	Cannabis	−3.1308	3.5121	−0.8915	0.3739	−0.0012	1.0467	0.7947	0.3739	29.91	–
Anencephalus	Cannabis	−18.7394	19.5370	−0.9592	0.3382	−0.0003	7.5930	0.9200	0.3382	18.38	–
Transposition of great arteries	Cannabis	−4.6234	3.9852	−1.1601	0.2469	0.0012	1.5252	1.3459	0.2469	31.05	–
Craniosynostosis	Cannabis	−38.5041	18.9772	−2.0290	0.0454	0.0328	3.8024	4.1167	0.0454	2.01E+04	–
Reduction deformity, Upper limbs	Cannabis	−22.0440	7.9002	−2.7903	0.0061	0.0519	1.5068	7.7858	0.0061	1.21E+06	–

Supplementary Table 6 performs the same function for estimated THC exposure. In this Table 40 ETOPFACARs have minimum E-Values greater than 1.00. Chromosomal and cardiovascular defects are particularly featured but microtia, limb and leg reduction defects, club foot, gastroschisis, omphalocele, anencephalus, spina bifida, esophageal atresia, small and large intestinal stenosis or atresia and obstructive genitourinary defects and congenital posterior urethral valves also feature.

As shown in Table 3 the list of ETOPFACARs with minimum E-Values greater than 1.00 is shorter for cannabidiol. Eleven defects are featured which are in order: congenital dislocation of the hip, small intestinal stenosis or atresia, biliary atresia, obstructive genitourinary defect, large bowel atresia or stenosis, Hirschsprungs disease (congenital megacolon), esophageal atresia, diaphragmatic hernia cleft palate, reduction deformities of the legs and transposition of the great vessels.

**Table 3:** Regression Slopes for ETOPFA-Corrected Congenital Anomaly Rates by Cannabidiol Exposure

		Parameters				Model				E-Values	
Congenital Anomaly	Term	Estimate	Std.Error	t-Value	P_Value	Adj.R.Squared	S.D.	t-Statistic	***P***-Value	E-Value - Point	E-Value - Lower
Congenital hip dislocation	Cannabidiol	298.2937	55.1100	5.4127	6.32E-07	0.2589	3.8459	29.2973	6.32E-07	9.00E+30	7.53E+19
Small intestinal atresia/stenosis	Cannabidiol	61.6605	12.7480	4.8369	3.39E-06	0.1354	1.1814	23.3954	3.39E-06	8.48E+20	3.86E+12
Biliary atresia	Cannabidiol	10.9598	2.9445	3.7222	2.43E-04	0.0480	0.3922	13.8546	2.43E-04	2.22E+11	3.48E+05
Obstructive genitourinary defect	Cannabidiol	486.0939	176.6878	2.7511	0.0072	0.0680	13.0815	7.5688	0.0072	9.69E+14	3.51E+04
Hirschsprung disease (congenital megacolon)	Cannabidiol	38.1800	14.1676	2.6949	0.0084	0.0637	1.0029	7.2624	0.0084	2.22E+15	2.67E+04
Rectal and large intestinal atresia/stenosis	Cannabidiol	26.0458	8.9678	2.9044	0.0040	0.0274	1.3051	8.4354	0.0040	1.54E+08	751.61
Esophageal atresia/tracheoesophageal fistula	Cannabidiol	13.7132	4.8352	2.8361	0.0049	0.0253	0.7108	8.0437	0.0049	8.43E+07	464.16
Diaphragmatic hernia	Cannabidiol	21.8501	7.9675	2.7424	0.0065	0.0237	1.1678	7.5207	0.0065	4.96E+07	263.36
Cleft palate alone	Cannabidiol	46.0706	20.0476	2.2981	0.0224	0.0172	2.7752	5.2811	0.0224	7.27E+06	18.43
Reduction deformity, Lower limbs	Cannabidiol	42.6901	21.4422	1.9909	0.0492	0.0288	1.6564	3.9638	0.0492	3.07E+10	2.38
Transposition of great arteries	Cannabidiol	19.6282	9.8766	1.9873	0.0479	0.0106	1.4902	3.9496	0.0479	3.21E+05	1.71
Cloacal exstrophy	Cannabidiol	76.8088	39.8261	1.9286	0.0563	0.0231	2.7733	3.7195	0.0563	1.76E+11	1.00
Epispadias	Cannabidiol	19.8920	10.4475	1.9040	0.0604	0.0307	0.7526	3.6252	0.0604	5.58E+10	1.00
Clubfoot	Cannabidiol	123.4731	76.7503	1.6088	0.1102	0.0123	5.7575	2.5881	0.1102	5.98E+08	1.00
Deletion 22q11.2	Cannabidiol	11.7674	7.4174	1.5865	0.1154	0.0128	0.5307	2.5169	0.1154	1.16E+09	1.00
Pulmonary valve atresia	Cannabidiol	14.2898	9.1696	1.5584	0.1208	0.0075	1.0200	2.4285	0.1208	6.89E+05	1.00
Aniridia	Cannabidiol	11.4134	7.6646	1.4891	0.1403	0.0146	0.4236	2.2174	0.1403	8.91E+10	1.00
Cleft lip with and without cleft palate	Cannabidiol	47.9755	35.6346	1.3463	0.1812	0.0078	2.8627	1.8126	0.1812	8.40E+06	1.00
Hypospadias	Cannabidiol	215.6799	160.8491	1.3409	0.1811	0.0029	24.1209	1.7980	0.1811	6.84E+03	1.00
Interrupted aortic arch	Cannabidiol	12.3060	10.7579	1.1439	0.2546	0.0022	0.8940	1.3085	0.2546	5.51E+05	1.00
Cleft lip with cleft palate	Cannabidiol	18.8934	17.5531	1.0764	0.2832	8.75E-04	1.8366	1.1585	0.2832	2.33E+04	1.00
Bladder exstrophy	Cannabidiol	1.2975	1.2080	1.0741	0.2838	6.22E-04	0.1585	1.1537	0.2838	3.44E+03	1.00
Total anomalous pulmonary venous connection	Cannabidiol	4.1211	4.3730	0.9424	0.3473	−6.29E-04	0.5022	0.8881	0.3473	3.50E+03	1.00
Congenital cataract	Cannabidiol	4.4981	7.4397	0.6046	0.5460	−0.0024	1.0520	0.3655	0.5460	97.42	1.00
Dextro-transposition of great arteries (d-TGA)	Cannabidiol	5.4634	9.0926	0.6009	0.5487	−0.0036	1.0480	0.3610	0.5487	229.30	1.00
Aortic valve stenosis	Cannabidiol	9.0139	15.8331	0.5693	0.5696	−0.0025	2.1657	0.3241	0.5696	87.79	1.00
Microcephalus	Cannabidiol	25.8143	51.1470	0.5047	0.6150	−0.0082	3.7981	0.2547	0.6150	970.31	1.00
Cleft lip alone	Cannabidiol	10.5197	24.3612	0.4318	0.6664	−0.0045	2.3987	0.1865	0.6664	107.70	1.00
Tetralogy of Fallot	Cannabidiol	4.4464	10.6210	0.4186	0.6758	−0.0029	1.6091	0.1753	0.6758	24.21	1.00
Patent ductus arteriosus	Cannabidiol	104.0952	399.2821	0.2607	0.7950	−0.0114	28.5501	0.0680	0.7950	54.70	1.00
Congenital posterior urethral valves	Cannabidiol	2.4488	20.4918	0.1195	0.9050	−0.0069	1.6854	0.0143	0.9050	6.96	1.00
Ventricular septal defect	Cannabidiol	−23.7333	139.2946	−0.1704	0.8648	−0.0037	20.2028	0.0290	0.8648	5.27	NA
Choanal atresia	Cannabidiol	−1.0493	5.0746	−0.2068	0.8363	−0.0036	0.7189	0.0428	0.8363	7.01	NA
Limb deficiencies (reduction defects)	Cannabidiol	−9.2029	28.6458	−0.3213	0.7484	−0.0051	2.6608	0.1032	0.7484	46.05	NA
Single ventricle	Cannabidiol	−3.9066	11.5847	−0.3372	0.7364	−0.0059	0.9895	0.1137	0.7364	72.15	NA
Pulmonary valve atresia and stenosis	Cannabidiol	−22.4845	34.4394	−0.6529	0.5144	−0.0021	5.0837	0.4262	0.5144	111.44	NA
Gastroschisis	Cannabidiol	−9.7272	13.2026	−0.7368	0.4619	−0.0018	1.9055	0.5428	0.4619	207.72	NA
Coarctation of the aorta	Cannabidiol	−27.6410	27.9781	−0.9880	0.3240	−8.62E-05	4.2271	0.9760	0.3240	767.41	NA
Common truncus (truncus arteriosus)	Cannabidiol	−9.2806	8.3564	−1.1106	0.2677	8.54E-04	1.1939	1.2334	0.2677	2.36E+03	NA
Anophthalmia/microphthalmia	Cannabidiol	−14.7546	12.6122	−1.1699	0.2431	0.0014	1.7384	1.3686	0.2431	4.52E+03	NA
Encephalocele	Cannabidiol	−11.9747	9.2992	−1.2877	0.1989	0.0024	1.3294	1.6582	0.1989	7.26E+03	NA
Atrial septal defect	Cannabidiol	−610.8850	361.7188	−1.6888	0.0925	0.0071	52.7719	2.8522	0.0925	7.52E+04	NA
Atrioventricular septal defect	Cannabidiol	−31.8080	16.5300	−1.9243	0.0554	0.0099	2.4231	3.7028	0.0554	3.08E+05	NA
Hydrocephalus without spina bifida	Cannabidiol	−56.5887	54.7420	−1.0337	0.3040	7.37E-04	4.0806	1.0686	0.3040	6.05E+05	NA
Holoprosencephaly	Cannabidiol	−146.0616	130.5839	−1.1185	0.2652	0.0017	10.2919	1.2511	0.2652	8.12E+05	NA
Turner syndrome	Cannabidiol	−103.2404	93.9159	−1.0993	0.2736	0.0015	7.1498	1.2084	0.2736	1.02E+06	NA
Hypoplastic left heart syndrome	Cannabidiol	−21.8275	9.7759	−2.2328	0.0263	0.0137	1.4903	4.9854	0.0263	1.23E+06	NA
Amniotic Bands	Cannabidiol	−8.5621	9.9023	−0.8647	0.3909	−0.0044	0.5072	0.7476	0.3909	9.39E+06	NA
Double outlet right ventricle	Cannabidiol	−17.4959	10.8027	−1.6196	0.1073	0.0099	1.0255	2.6230	0.1073	1.11E+07	NA
Anotia/microtia	Cannabidiol	−75.4583	28.5853	−2.6398	0.0088	0.0215	4.1677	6.9683	0.0088	2.86E+07	NA
Renal agenesis/hypoplasia	Cannabidiol	−55.5432	21.1694	−2.6237	0.0092	0.0213	3.0263	6.8841	0.0092	3.59E+07	NA
Omphalocele	Cannabidiol	−72.7238	25.9206	−2.8056	0.0054	0.0273	3.5964	7.8716	0.0054	1.96E+08	NA
Tricuspid valve atresia and stenosis	Cannabidiol	−66.1395	18.6247	−3.5512	4.53E-04	0.0414	2.7738	12.6108	4.53E-04	5.30E+09	NA
Spina bifida without anencephalus	Cannabidiol	−100.5390	26.3548	−3.8148	1.67E-04	0.0446	4.0223	14.5529	1.67E-04	1.51E+10	NA
Trisomy 21 (Down syndrome)	Cannabidiol	−294.7787	68.5834	−4.2981	2.36E-05	0.0568	10.4764	18.4737	2.36E-05	2.64E+11	NA
Trisomy 13	Cannabidiol	−159.8606	36.7241	−4.3530	1.90E-05	0.0617	5.3524	18.9488	1.90E-05	1.27E+12	NA
Ebstein anomaly	Cannabidiol	−13.6797	3.1851	−4.2949	2.46E-05	0.0620	0.4446	18.4464	2.46E-05	2.90E+12	NA
Reduction deformity, Upper limbs	Cannabidiol	−50.5739	20.4153	−2.4773	0.0150	0.0493	1.5707	6.1368	0.0150	1.06E+13	NA
Craniosynostosis	Cannabidiol	−128.6709	60.4738	−2.1277	0.0361	0.0369	3.7943	4.5272	0.0361	5.05E+13	NA
Trisomy 18	Cannabidiol	−376.7155	67.6238	−5.5708	5.95E-08	0.0966	10.0334	31.0333	5.95E-08	1.38E+15	NA
Anencephalus	Cannabidiol	−405.9858	49.6283	−8.1805	9.98E-15	0.1900	7.0466	66.9210	9.98E-15	1.18E+23	NA

Hence from this series of data we note that the sequence of teratogens is THC (40 CAs) > cannabis (35 CAs) > tobacco (11 CAs) > cannabidiol (11 CAs) > monthly alcohol (5 CAs) > binge alcohol (2 CAs).

To aid with understanding and comparison these minimum E-Values are also presented graphically using a log scale. A horizontal line marks the literature described cut-off for causality at (log) 1.25 [67]. Supplementary Fig. 3 shows the minimum E-Values for ETOPFACARs over time.

**Fig. 8:**
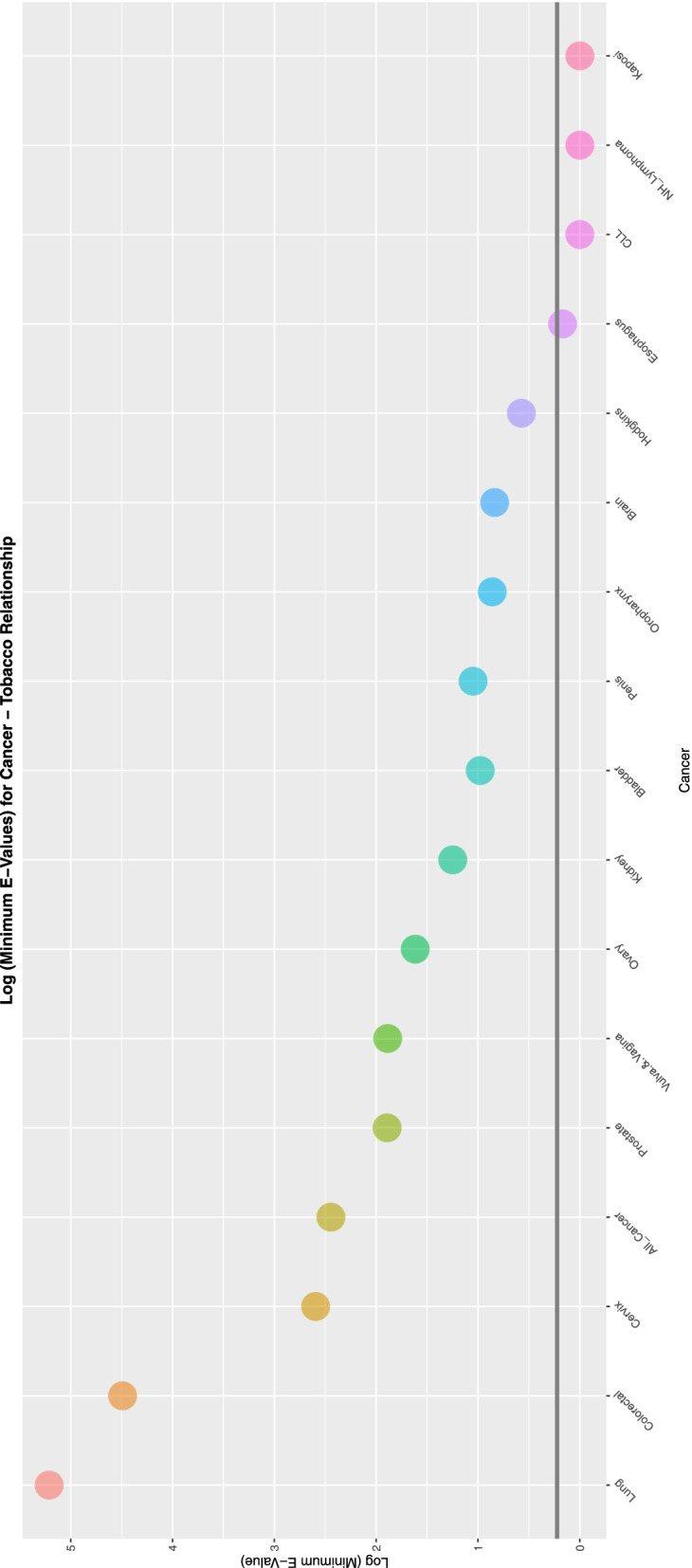
E-Values of regression lines of relationship of congenital anomaly incidence rates with tobacco exposure

Figure 8 lists the E-Values by CA for those ETOPFACARs which reported elevated finite minimum E-Values for tobacco.

**Fig. 9:**
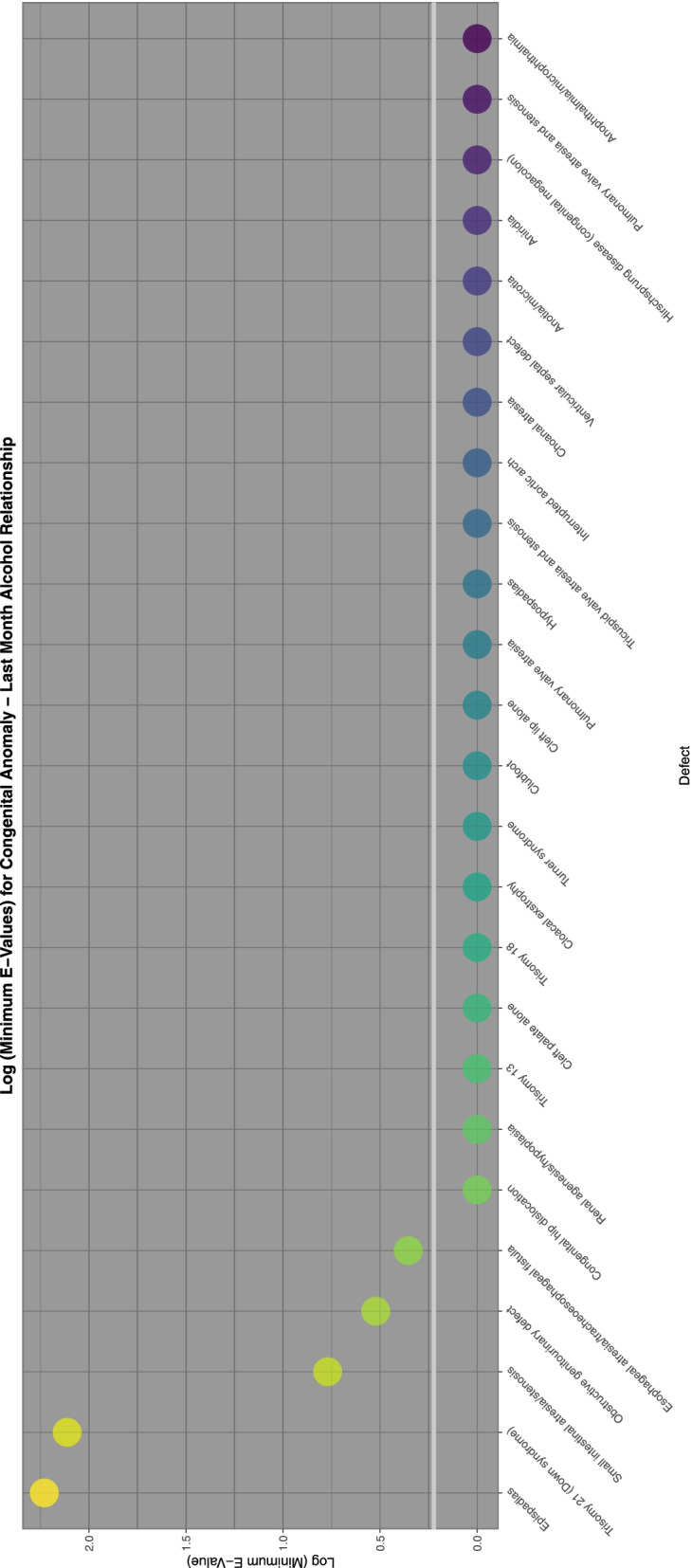
E-Values of regression lines of relationship of congenital anomaly incidence rates with last month exposure

**Fig. 10:**
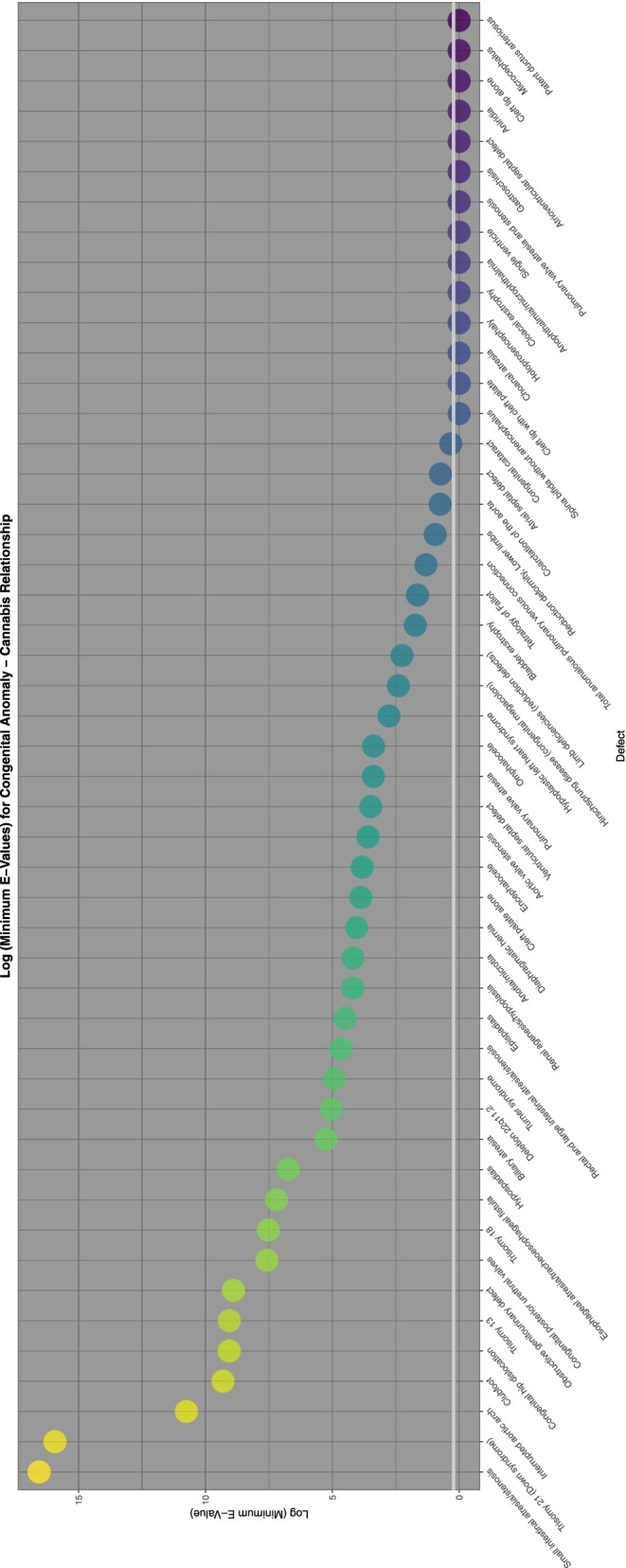
E-Values of regression lines of relationship of congenital anomaly incidence rates with cannabis exposure

**Fig. 11:**
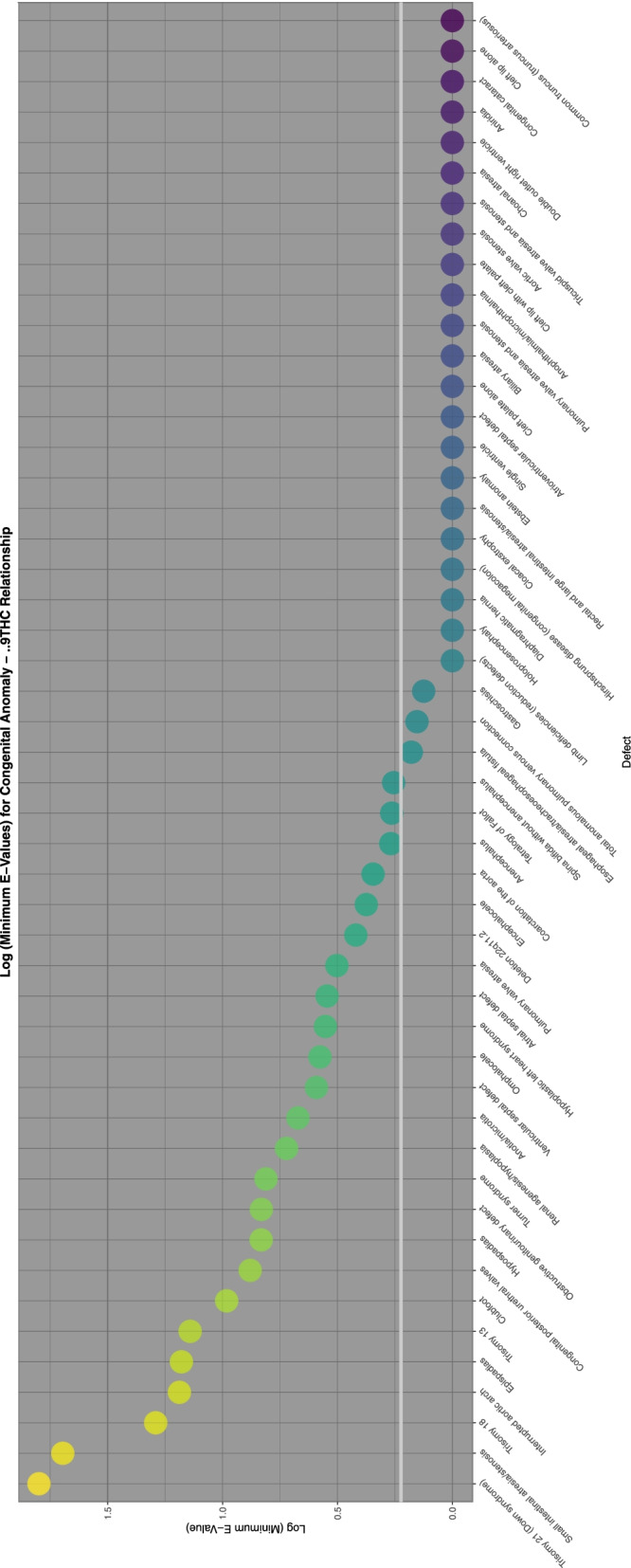
E-Values of regression lines of relationship of congenital anomaly incidence rates with THC exposure

**Fig. 12:**
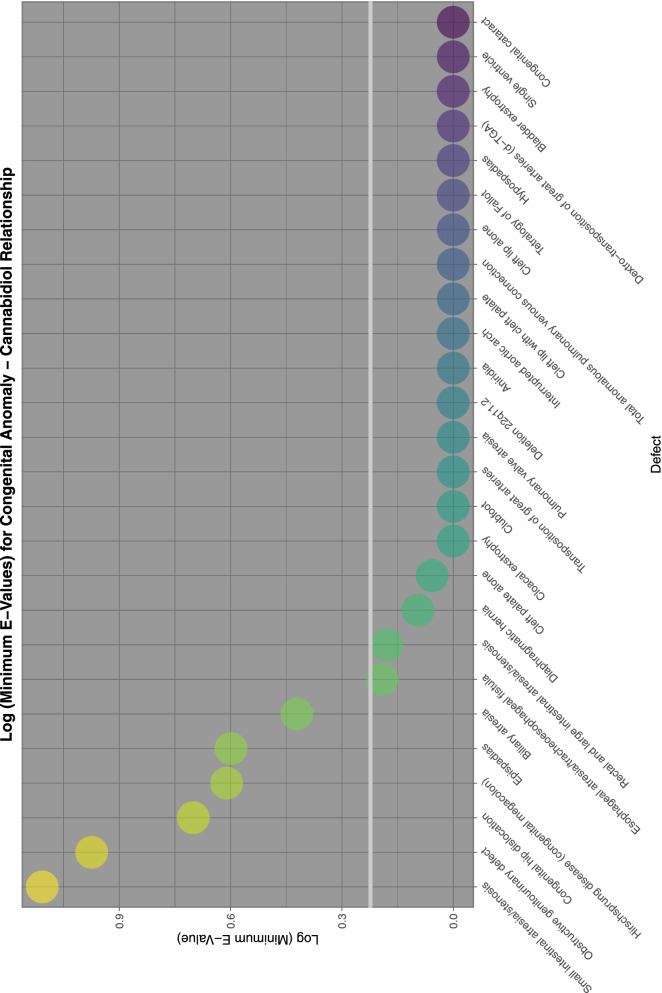
E-Values of regression lines of relationship of congenital anomaly incidence rates with cannabidiol exposure

Supplementary Fig. 4 and Figs. 9, 10, 11, 12 do this for binge alcohol, last month alcohol, cannabis, THC and cannabidiol exposure respectively. One notes that the graph for THC clearly has more defects listed.

### Categorical exposure survey

Exposure data was categorized to allow the calculation of key parameters of public health interest such as the prevalence ratio, the aetiological fraction in the exposed and the population attributable risk.

In the following categorical analysis the data was taken from the raw unadjusted NBDPN rates themselves i.e. ETOPFACARs were not used in this series.

**Fig. 13:**
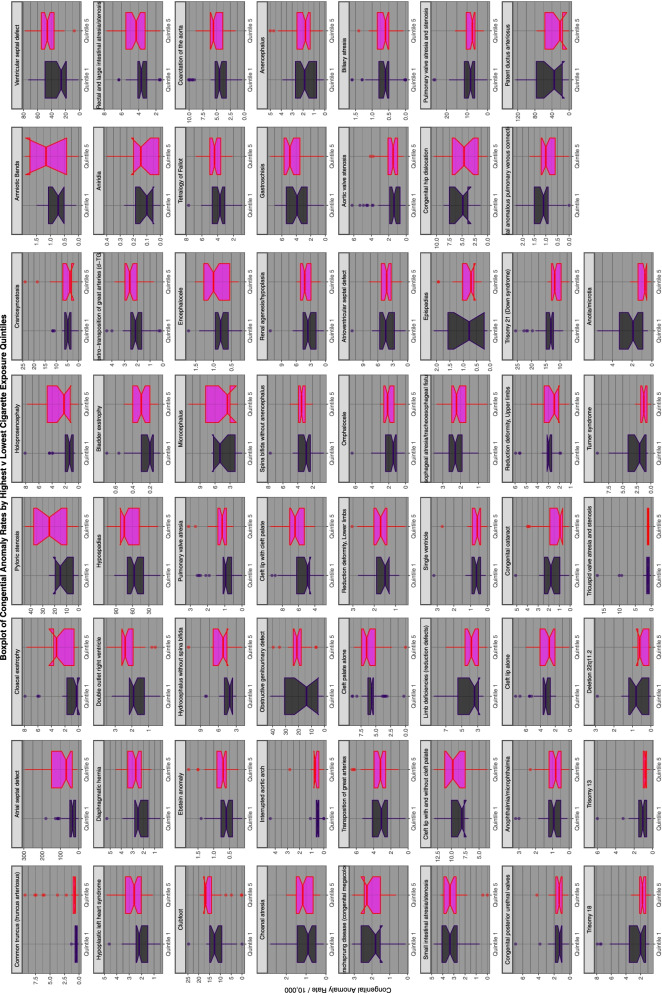
Categorical analysis of congenital anomaly incidence rates rates for extreme quintiles of tobacco exposure

Figure 13 shows boxplots by CA and contrasts the highest and lowest quintiles of cigarette exposure by CA listing them in the order of the decreasing ratios between the highest and lowest quintiles.

**Fig. 14:**
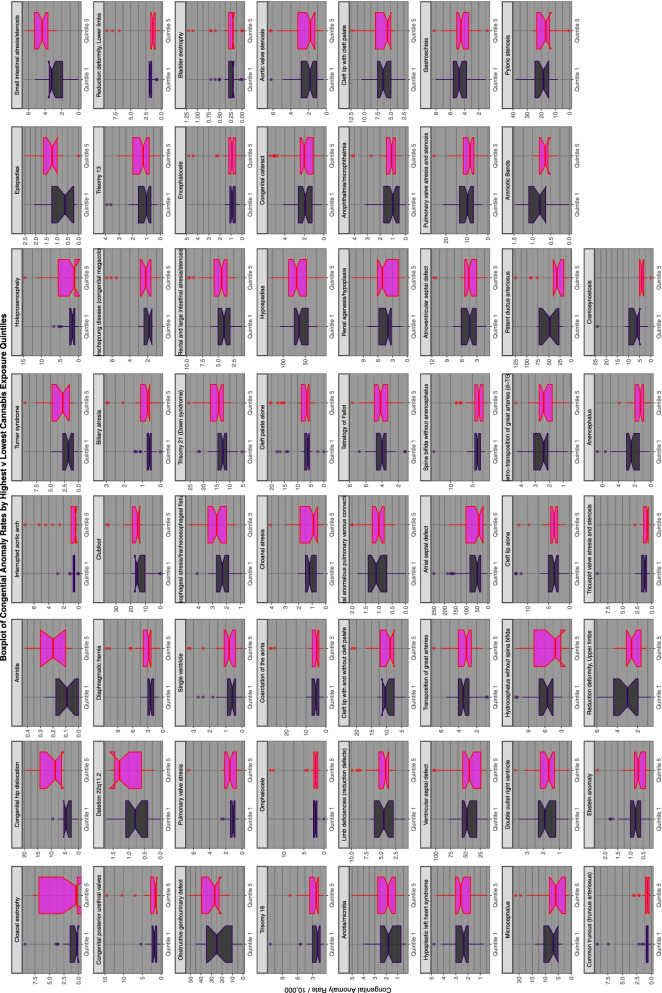
Categorical analysis of congenital anomaly incidence rates for extreme quintiles of last month cannabis exposure

**Fig. 15:**
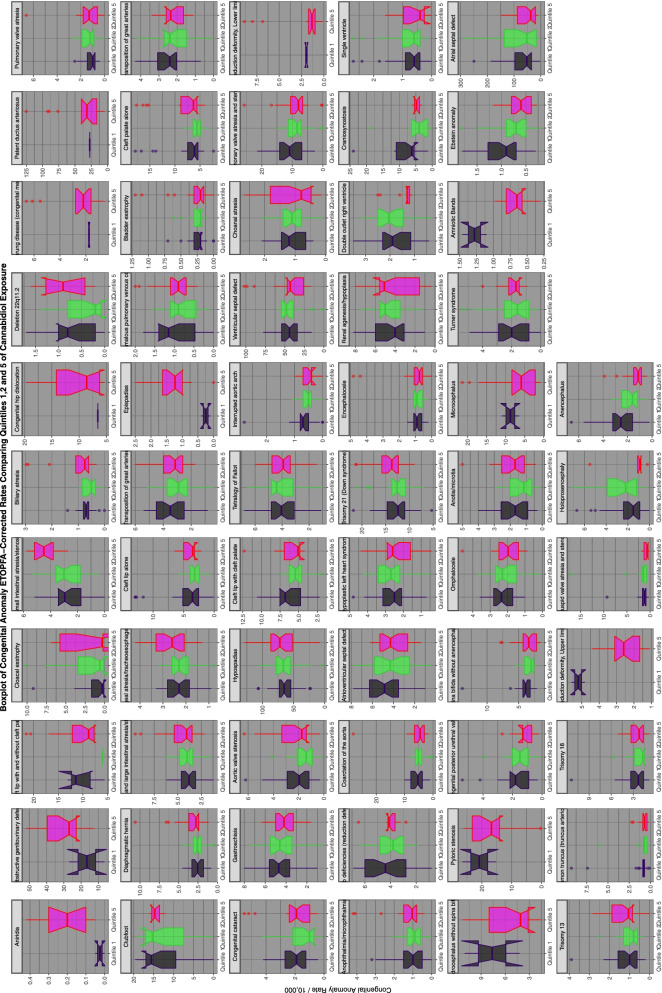
Categorical analysis of congenital anomaly incidence rates for extreme quintiles of canabidiol exposure

Supplementary Figs. 5, 6, 7, 8 and Figs. 14 and 15 do this for binge alcohol, last month alcohol, analgesic, cocaine, last month cannabis and cannabidiol exposure. Cannabidiol quintiles in Fig. 15 are not grouped by year but calculated across the whole period.

Supplementary Table 7 presents the numbers born with and without CAs in the highest and lowest quintiles of tobacco use states. The Prevalence Ratio (like the Odds Ratio for cohort studies), Attributable Fraction in the Exposed (AFE), the Population Attributable Risk (PAR), the Chi Squared value and the P-level of significance is also shown. The right most columns show the point estimate for the E-Value together with its 95% lower bound. In this Supplementary Table 7 defects are noted to have minimum E-Values elevated above 1.00.

Supplementary Tables 8, 9, 10 and Tables 4, 5 perform a similar function for binge alcohol, analgesics, cocaine, cannabis and cannabidiol respectively. As the CAs tracked by NBDPN / CDC changed over time as the cannabidiol exposure was falling 11 defects have no entries in Quintile 1 (see Fig. 15 for details). Numbers exposed in Quintile 2 were used for these CAs. In these five tables one notes respectively that 1, 21, 27, 10 and 11 CAs demonstrate elevated minimum E-Values. These data suggest that cannabis (21 defects) is the third most important teratogen behind analgesics (27 CAs) and tobacco (26 CAs). Teratogenesis from cannabidiol also appears to be significant (11 CAs).

**Table 4:** Numbers, Calculated Rates, Significance Levels and E-Values of Highest v. Lowest Cannabis Exposure Quintiles

Congenital Anomaly	Numbers				Calculated Rates			Significance		E-Values	
	Highest Defect Count	Highest Not Defect Count	Lowest Defect Count	Lowest Not Defect Count	Prevalence Ratio (C.I.)	Atrributable Fraction in the Exposed (C.I.)	Population Attributable Risk (C.I.)	Chi Squared	***P***-Value	E-Value - Point	E-Value - Lower
Cloacal exstrophy	444	1,141,378	177	2,207,096	4.8507 (4.075, 5.774)	0.7938 (0.7545, 0.8268)	0.5675 (0.5102, 0.6182)	386.73	2.13E-86	9.17	7.61
Congenital hip dislocation	773	722,717	973	2,078,182	2.2845 (2.0785, 2.5108)	0.562 (0.5186, 0.6014)	0.2488 (0.2167, 0.2796)	310.82	7.27E-70	3.99	3.57
Turner syndrome	1577	956,504	3159	2,999,000	1.5652 (1.4734, 1.6628)	0.3607 (0.3209, 0.3982)	0.1201 (0.1022, 0.1376)	214.37	7.69E-49	2.50	2.31
Coarctation of the aorta	3630	3,825,817	3787	5,516,098	1.382 (1.3205, 1.4464)	0.2762 (0.2425, 0.3084)	0.1352 (0.1157, 0.1542)	195.57	9.74E-45	2.11	1.97
Trisomy 18	7276	3,863,762	7853	5,454,321	1.3079 (1.2668, 1.3504)	0.2351 (0.2103, 0.2591)	0.1131 (0.0994, 0.1266)	273.35	1.06E-61	1.94	1.85
Hirschsprung disease (congenital megacolon)	230	943,071	372	2,220,573	1.4558 (1.2351, 1.716)	0.313 (0.1903, 0.4172)	0.1196 (0.0625, 0.1732)	20.28	6.69E-06	2.27	1.77
Trisomy 13	3310	3,809,527	3677	5,440,392	1.2856 (1.2266, 1.3474)	0.222 (0.1846, 0.2577)	0.1052 (0.085, 0.1248)	110.41	3.50E-06	1.89	1.75
Holoprosencephaly	2307	2,735,097	2013	2,952,020	1.2369 (1.1651, 1.3132)	0.1914 (0.1416, 0.2383)	0.1022 (0.0731, 0.1304)	48.76	2.90E-12	1.78	1.60
Diaphragmatic hernia	1210	3,785,854	1417	5,518,468	1.2447 (1.1528, 1.344)	0.1966 (0.1325, 0.2559)	0.0905 (0.0578, 0.1221)	31.39	2.11E-08	1.80	1.57
Congenital posterior urethral valves	272	1,222,110	516	3,083,046	1.3298 (1.1482, 1.5402)	0.248 (0.129, 0.3507)	0.0856 (0.038, 0.1308)	14.57	1.35E-04	1.99	1.56
Pulmonary valve atresia	622	3,217,047	573	3,778,215	1.2749 (1.1381, 1.4281)	0.2156 (0.1213, 0.2997)	0.1122 (0.0582, 0.1631)	17.67	2.62E-05	1.87	1.53
Small intestinal atresia/stenosis	1125	2,778,116	957	2,890,697	1.2232 (1.1222, 1.3333)	0.1824 (0.1088, 0.2499)	0.0986 (0.0556, 0.1396)	21.05	4.47E-06	1.75	1.49
Trisomy 21 (Down syndrome)	17,749	4,160,407	20,309	5,441,865	1.1431 (1.1203, 1.1664)	0.1247 (0.107, 0.1422)	0.0582 (0.0493, 0.067)	169.07	4.02E-26	1.55	1.49
Deletion 22q11.2	129	1,175,941	236	2,919,417	1.357 (1.0949, 1.6819)	0.2631 (0.0867, 0.4054)	0.093 (0.0215, 0.1592)	7.83	0.0051	2.05	1.42
Double outlet right ventricle	854	2,779,998	684	2,647,487	1.189 (1.0752, 1.3148)	0.1589 (0.07, 0.2394)	0.0883 (0.0359, 0.1378)	11.41	7.31E-04	1.66	1.36
Single ventricle	435	2,750,022	377	2,891,287	1.2131 (1.0568, 1.3925)	0.1757 (0.0538, 0.2818)	0.0941 (0.0246, 0.1586)	7.56	0.0060	1.72	1.30
Hypoplastic left heart syndrome	1608	3,991,321	2023	5,517,862	1.0989 (1.0292, 1.1732)	0.0899 (0.0284, 0.1476)	0.0398 (0.0116, 0.0673)	7.97	0.0048	1.43	1.20
Epispadias	93	733,098	214	2,211,891	1.3112 (1.0279, 1.6726)	0.2373 (0.0271, 0.4021)	0.0719 (9e-04, 0.1379)	4.79	0.0287	1.95	1.20
Biliary atresia	303	3,696,288	367	5,330,359	1.1906 (1.0226, 1.3862)	0.1601 (0.0221, 0.2786)	0.0724 (0.0063, 0.1341)	5.06	0.0244	1.67	1.17
Esophageal atresia/tracheoesophageal fistula	924	3,774,953	1207	5,460,967	1.1074 (1.0165, 1.2065)	0.097 (0.0162, 0.1711)	0.0421 (0.0058, 0.077)	5.45	0.0195	1.45	1.15
Clubfoot	1709	1,072,816	4038	2,722,058	1.0739 (1.0148, 1.1364)	0.0687 (0.0145, 0.1199)	0.0204 (0.0038, 0.0367)	6.09	0.0136	1.36	1.14
Spina bifida without anencephalus	4268	4,086,425	5488	5,514,397	1.0495 (1.0083, 1.0923)	0.0471 (0.0082, 0.0844)	0.0206 (0.0033, 0.0376)	5.59	0.0181	1.28	1.10
Atrioventricular septal defect	2405	3,784,659	3324	5,516,561	1.0546 (1.0007, 1.1114)	0.0518 (7e-04, 0.1002)	0.0217 (−1e-04, 0.043)	3.94	0.0470	1.29	1.03
Aniridia	32	866,880	55	2,154,589	1.4461 (0.9353, 2.2359)	0.3085 (−0.0692, 0.5527)	0.1135 (−0.0407, 0.2448)	2.78	0.0952	2.25	1.00
Total anomalous pulmonary venous connection	470	3,196,783	494	3,532,159	1.0512 (0.9265, 1.1928)	0.0487 (−0.0793, 0.1616)	0.0238 (−0.0382, 0.0821)	0.60	0.4381	1.28	1.00
Hydrocephalus without spina bifida	1057	954,941	2408	2,276,248	1.0463 (0.9733, 1.1248)	0.0442 (−0.0274, 0.1109)	0.0135 (−0.0085, 0.035)	1.50	0.2200	1.27	1.00
Interrupted aortic arch	184	2,761,460	180	2,801,964	1.0372 (0.8446, 1.2738)	0.0359 (−0.184, 0.2149)	0.0181 (−0.0893, 0.115)	0.12	0.7274	1.23	1.00
Bladder exstrophy	60	2,670,828	115	5,256,233	1.0268 (0.7515, 1.4029)	0.0261 (−0.3307, 0.2872)	0.0089 (−0.103, 0.1095)	0.03	0.8681	1.19	1.00
Transposition of great arteries	1049	2,418,662	2201	5,131,566	1.0112 (0.9395, 1.0884)	0.0111 (−0.0644, 0.0812)	0.0036 (−0.0204, 0.0269)	0.09	0.7669	1.12	1.00
Anotia/microtia	3396	3,783,668	4802	5,355,143	1.0009 (0.9579, 1.0459)	9e-04 (−0.0439, 0.0439)	4e-04 (−0.018, 0.0184)	0.00	0.9670	1.03	1.00
Cleft lip alone	1178	2,903,931	1366	3,261,461	0.9685 (0.8959, 1.0471)	−0.0325 (−0.1161, 0.0449)	−0.015 (−0.0523, 0.0209)	0.65	0.4216	1.22	NA
Tetralogy of Fallot	2063	3,991,594	2966	5,516,919	0.9613 (0.9088, 1.0169)	−0.0402 (−0.1003, 0.0166)	−0.0165 (−0.0402, 0.0067)	1.89	0.1692	1.24	NA
Cleft palate alone	2483	3,990,680	3402	5,227,758	0.9561 (0.9079, 1.0069)	−0.0459 (−0.1014, 0.0068)	−0.0194 (−0.0418, 0.0026)	2.89	0.0892	1.26	NA
Cleft lip with cleft palate	1907	3,032,425	2001	3,038,883	0.9551 (0.897, 1.0169)	−0.047 (−0.1148, 0.0166)	−0.0229 (−0.0547, 0.0079)	2.06	0.1508	1.27	NA
Congenital cataract	670	3,755,999	1016	5,381,175	0.9448 (0.857, 1.0416)	−0.0584 (−0.1669, 0.0399)	−0.0232 (−0.0637, 0.0157)	1.30	0.2537	1.31	NA
Encephalocele	932	3,791,070	1446	5,518,439	0.9382 (0.8641, 1.0187)	−0.0658 (−0.1573, 0.0184)	−0.0258 (−0.0594, 0.0068)	2.31	0.1289	1.33	NA
Cleft lip with and without cleft palate	969	1,026,247	2304	2,286,080	0.9369 (0.8691, 1.0099)	−0.0673 (−0.1504, 0.0098)	−0.0199 (−0.0428, 0.0025)	2.90	0.0887	1.34	NA
Gastroschisis	2165	4,016,001	3073	5,301,775	0.9301 (0.8803, 0.9827)	−0.0751 (−0.1359, −0.0176)	−0.0311 (−0.0548, −0.0079)	6.67	0.0098	1.36	NA
Anencephalus	4669	4,093,278	6782	5,513,103	0.9272 (0.8933, 0.9625)	−0.0784 (−0.1193, −0.0389)	−0.032 (−0.0477, −0.0164)	15.77	7.15E-05	1.37	NA
Omphalocele	1876	3,828,924	2621	4,919,500	0.9196 (0.8667, 0.9758)	−0.0874 (−0.1537, −0.0248)	−0.0364 (−0.0624, −0.0111)	7.68	0.0056	1.40	NA
Rectal and large intestinal atresia/stenosis	1581	3,785,483	2532	5,459,642	0.9006 (0.8457, 0.959)	−0.1104 (−0.1824, −0.0428)	−0.0424 (−0.0679, −0.0176)	10.68	0.0011	1.46	NA
Aortic valve stenosis	1391	3,730,418	2298	5,517,587	0.8953 (0.8376, 0.957)	−0.1169 (−0.1938, −0.045)	−0.0441 (−0.0706, −0.0182)	10.61	0.0011	1.48	NA
Pyloric stenosis	1264	709,529	4414	2,216,531	0.8946 (0.8403, 0.9524)	−0.1176 (−0.1897, −0.0499)	−0.0262 (−0.0406, −0.012)	12.19	4.82E-04	1.48	NA
Ebstein anomaly	360	3,742,751	592	5,439,310	0.8838 (0.7752, 1.0075)	−0.1315 (−0.2899, 0.0074)	−0.0497 (−0.103, 0.001)	3.42	0.0643	1.52	NA
Pulmonary valve atresia and stenosis	2810	2,731,571	6450	5,513,435	0.8793 (0.8412, 0.9192)	−0.1371 (−0.1885, −0.0878)	−0.0416 (−0.0557, −0.0277)	32.37	1.27E-08	1.53	NA
Obstructive genitourinary defect	2840	953,158	7681	2,213,264	0.8586 (0.8223, 0.8964)	−0.1642 (−0.2153, −0.1152)	−0.0443 (−0.0565, −0.0323)	48.16	3.92E-12	1.60	NA
Amniotic Bands	32	483,538	162	2,064,910	0.8435 (0.5773, 1.2325)	−0.1855 (−0.732, 0.1886)	−0.0306 (−0.0971, 0.0319)	0.78	0.3785	1.65	NA
Reduction deformity, Lower limbs	215	1,012,889	578	2,278,078	0.8366 (0.7153, 0.9784)	−0.1953 (−0.3978, −0.0221)	−0.0529 (−0.0986, −0.0092)	5.00	0.0253	1.68	NA
Choanal atresia	423	3,737,112	798	5,467,622	0.7755 (0.6893, 0.8726)	−0.2894 (−0.4507, −0.146)	−0.1003 (−0.1461, −0.0562)	17.96	2.26E-05	1.90	NA
Limb deficiencies (reduction defects)	1947	3,044,005	2616	3,159,025	0.7724 (0.7284, 0.8191)	−0.2944 (−0.3726, −0.2207)	−0.1256 (−0.1542, −0.0978)	74.81	2.33E-06	1.91	NA
Dextro-transposition of great arteries (d-TGA)	787	3,139,477	1263	3,768,524	0.748 (0.6843, 0.8176)	−0.3368 (−0.4613, −0.223)	−0.1293 (−0.1686, −0.0914)	41.16	1.40E-10	2.01	NA
Ventricular septal defect	10,038	2,237,688	33,128	5,486,757	0.743 (0.7265, 0.7598)	−0.3439 (−0.3742, −0.3143)	−0.08 (−0.0856, −0.0744)	681.68	0.0033	2.02	NA
Hypospadias	19,468	4,144,576	34,580	5,427,594	0.7373 (0.7244, 0.7504)	−0.3541 (−0.378, −0.3306)	−0.1275 (−0.1347, −0.1205)	1159.93	1.38E-05	2.05	NA
Common truncus (truncus arteriosus)	265	3,819,026	521	5,470,721	0.7286 (0.6285, 0.8447)	−0.3724 (−0.5911, −0.1838)	−0.1256 (−0.1831, −0.0708)	17.75	2.51E-05	2.09	NA
Atrial septal defect	17,822	3,769,242	36,035	5,426,139	0.712 (0.6993, 0.7249)	−0.4019 (−0.4272, −0.377)	−0.133 (−0.1397, −0.1263)	1381.73	1.09E-08	2.15	NA
Microcephalus	595	955,403	1996	2,276,660	0.7103 (0.6482, 0.7785)	−0.4074 (−0.5423, −0.2843)	−0.0936 (−0.1168, −0.0708)	54.10	1.90E-13	2.16	NA
Reduction deformity, Upper limbs	381	998,572	1239	2,277,417	0.7013 (0.6252, 0.7867)	−0.4256 (−0.5991, −0.271)	−0.1001 (−0.1302, −0.0708)	37.05	1.15E-09	2.20	NA
Anophthalmia/microphthalmia	1081	3,720,258	2643	5,328,083	0.5858 (0.5457, 0.6287)	−0.7068 (−0.8319, −0.5902)	−0.2052 (−0.2302, −0.1807)	224.65	1.48E-12	2.81	NA
Patent ductus arteriosus	3025	952,973	10,938	1,995,801	0.5792 (0.5563, 0.603)	−0.7226 (−0.7932, −0.6547)	−0.1565 (−0.1667, −0.1465)	721.65	5.95E-39	2.84	NA
Craniosynostosis	709	1,970,408	1706	2,570,932	0.5423 (0.4968, 0.5919)	−0.8436 (−1.0123, −0.689)	−0.2477 (−0.2802, −0.216)	193.44	7.18E-05	3.09	NA
Renal agenesis/hypoplasia	1705	3,788,008	4576	5,457,598	0.5368 (0.5078, 0.5675)	−0.8621 (−0.9686, −0.7614)	−0.234 (−0.2528, −0.2155)	496.12	0.0028	3.13	NA
Tricuspid valve atresia and stenosis	643	3,773,660	1767	5,518,118	0.5321 (0.4862, 0.5824)	−0.879 (−1.0565, −0.7169)	−0.2345 (−0.2646, −0.2052)	193.92	3.93E-04	3.16	NA

**Table 5:** Numbers, Calculated Rates, Significance Levels and E-Values of Highest v. Lowest Cannabidiol Exposure Quintiles

Congenital Anomaly	Numbers				Calculated Rates			Significance		E-Values	
	Highest Defect Count	Highest Not Defect Count	Lowest Defect Count	Lowest Not Defect Count	Prevalence Ratio (C.I.)	Atrributable Fraction in the Exposed (C.I.)	Population Attributable Risk (C.I.)	Chi Squared	***P***-Value	E-Value - Point	E-Value - Lower
Obstructive genitourinary defect	7247	2,484,854	145	95,592	1.9227 (1.631, 2.2665)	0.4792 (0.3862, 0.5581)	0.4698 (0.3771, 0.5486)	62.8480	2.22E-15	3.25	2.64
Pulmonary valve atresia	396	2,552,400	462	4,024,227	1.3514 (1.1817, 1.5455)	0.26 (0.1537, 0.3529)	0.12 (0.0638, 0.1729)	19.4818	1.02E-05	2.04	1.64
Small intestinal atresia/stenosis	566	1,402,095	1224	3,815,218	1.2583 (1.1389, 1.3901)	0.2052 (0.122, 0.2806)	0.0649 (0.035, 0.0939)	20.5107	5.93E-06	1.83	1.54
Cloacal exstrophy	238	834,373	661	3,007,409	1.2978 (1.1191, 1.5051)	0.2294 (0.1064, 0.3355)	0.0607 (0.0232, 0.0969)	11.9548	5.45E-04	1.92	1.48
Cleft lip with and without cleft palate	3437	3,791,717	33	55,337	1.52 (1.0787, 2.1418)	0.3419 (0.0729, 0.5329)	0.3387 (0.0713, 0.529)	5.8113	0.0159	2.41	1.37
Clubfoot	1057	721,190	5114	3,838,593	1.1001 (1.0296, 1.1755)	0.0909 (0.0287, 0.1491)	0.0156 (0.0043, 0.0267)	7.9686	0.0048	1.43	1.20
Biliary atresia	385	5,083,733	265	4,192,774	1.1982 (1.0247, 1.4011)	0.1654 (0.0241, 0.2863)	0.098 (0.0104, 0.1778)	5.1462	0.0233	1.69	1.18
Trisomy 21 (Down syndrome)	7317	5,291,885	5706	4,357,620	1.0559 (1.02, 1.0932)	0.0529 (0.0196, 0.0851)	0.0297 (0.0107, 0.0484)	9.4889	0.0021	1.30	1.16
Double outlet right ventricle	282	1,402,379	745	4,282,993	1.156 (1.008, 1.3258)	0.135 (0.0079, 0.2457)	0.0371 (1e-04, 0.0726)	4.3080	0.0379	1.58	1.10
Diaphragmatic hernia	1433	5,035,560	1143	4,362,183	1.0861 (1.0048, 1.1739)	0.0792 (0.0048, 0.1481)	0.0441 (0.0018, 0.0845)	4.3354	0.0373	1.39	1.07
Trisomy 13	597	5,167,619	443	4,353,779	1.1354 (1.0041, 1.2839)	0.1192 (0.0041, 0.2211)	0.0684 (4e-04, 0.1319)	4.1053	0.0427	1.53	1.07
Single ventricle	136	1,397,584	315	3,963,023	1.2243 (1.0012, 1.497)	0.1832 (0.0012, 0.332)	0.0552 (−0.0038, 0.1108)	3.9021	0.0482	1.75	1.04
Spina bifida without anencephalus	1838	5,297,364	1509	4,361,817	1.0029 (0.9369, 1.0736)	0.0029 (−0.0673, 0.0685)	0.0016 (−0.0364, 0.0382)	0.0070	0.9332	1.06	1.00
Rectal and large intestinal atresia/stenosis	1883	4,468,696	1830	4,361,496	1.0043 (0.9417, 1.071)	0.0043 (−0.0619, 0.0663)	0.0022 (−0.0309, 0.0342)	0.0169	0.8966	1.07	1.00
Anotia/microtia	1049	5,138,925	856	4,221,402	1.0067 (0.9198, 1.1018)	0.0066 (−0.0872, 0.0924)	0.0036 (−0.0471, 0.052)	0.0208	0.8853	1.09	1.00
Transposition of great arteries	1324	4,204,444	1356	4,361,970	1.013 (0.9391, 1.0927)	0.0128 (−0.0648, 0.0848)	0.0063 (−0.0315, 0.0428)	0.1114	0.7385	1.13	1.00
Aortic valve stenosis	961	5,083,758	806	4,362,520	1.0232 (0.9317, 1.1236)	0.0226 (−0.0733, 0.11)	0.0123 (−0.0393, 0.0613)	0.2296	0.6318	1.18	1.00
Hypoplastic left heart syndrome	1308	5,297,894	1048	4,362,278	1.0277 (0.9475, 1.1147)	0.0269 (−0.0554, 0.1029)	0.0149 (−0.0305, 0.0584)	0.4336	0.5102	1.20	1.00
Cleft lip alone	478	1,444,653	1378	4,282,360	1.0282 (0.9266, 1.141)	0.0275 (−0.0792, 0.1235)	0.0071 (−0.0199, 0.0333)	0.2753	0.5998	1.20	1.00
Trisomy 18	1219	5,196,590	983	4,362,343	1.041 (0.9571, 1.1323)	0.0394 (−0.0448, 0.1168)	0.0218 (−0.0248, 0.0663)	0.8786	0.3486	1.25	1.00
Atrioventricular septal defect	2286	5,034,707	1877	4,361,449	1.055 (0.9925, 1.1215)	0.0521 (−0.0075, 0.1083)	0.0286 (−0.0045, 0.0607)	2.9581	0.0854	1.30	1.00
Patent ductus arteriosus	7335	2,484,766	111	40,496	1.077 (0.8927, 1.2993)	0.0713 (−0.1199, 0.2298)	0.0702 (−0.1181, 0.2268)	0.5999	0.4386	1.36	1.00
Total anomalous pulmonary venous connection	389	3,073,253	459	4,095,548	1.1294 (0.9867, 1.2928)	0.1146 (−0.0135, 0.2264)	0.0526 (−0.008, 0.1095)	3.1216	0.0773	1.51	1.00
Deletion 22q11.2	53	718,890	216	3,557,414	1.2142 (0.8991, 1.6398)	0.1764 (−0.1122, 0.3901)	0.0348 (−0.0241, 0.0902)	1.6082	0.2047	1.72	1.00
Congenital hip dislocation	1807	2,126,749	27	40,580	1.277 (0.8732, 1.8676)	0.2168 (−0.1452, 0.4643)	0.2136 (−0.1434, 0.4591)	1.5973	0.2063	1.87	1.00
Hirschsprung disease (congenital megacolon)	591	2,581,794	17	95,720	1.2889 (0.7958, 2.0875)	0.2241 (−0.2565, 0.5209)	0.2178 (−0.2497, 0.5105)	1.0699	0.3010	1.90	1.00
Gastroschisis	2169	4,985,466	1842	4,215,660	0.9957 (0.9357, 1.0595)	−0.0043 (−0.0686, 0.0561)	−0.0023 (−0.0366, 0.0308)	0.0185	0.8919	1.07	NA
Cleft lip with cleft palate	1220	2,126,674	2452	4,225,916	0.9887 (0.9231, 1.059)	−0.0114 (−0.0833, 0.0557)	−0.0038 (−0.027, 0.0188)	0.1054	0.7455	1.12	NA
Coarctation of the aorta	2728	5,193,099	2332	4,360,994	0.9824 (0.9295, 1.0382)	−0.0179 (−0.0758, 0.0368)	−0.0097 (−0.0402, 0.02)	0.3976	0.5283	1.15	NA
Anencephalus	920	5,240,706	775	4,279,449	0.9694 (0.881, 1.0666)	−0.0316 (−0.135, 0.0624)	−0.0172 (−0.0713, 0.0343)	0.4074	0.5233	1.21	NA
Esophageal atresia/tracheoesophageal fistula	1127	5,138,847	988	4,362,338	0.9683 (0.889, 1.0547)	−0.0327 (−0.1248, 0.0518)	−0.0174 (−0.0648, 0.0278)	0.5454	0.4602	1.22	NA
Tetralogy of Fallot	2121	5,286,622	1815	4,361,511	0.9641 (0.9055, 1.0265)	−0.0372 (−0.1043, 0.0258)	−0.0201 (−0.0551, 0.0138)	1.3068	0.2530	1.23	NA
Encephalocele	411	4,911,620	370	4,241,015	0.9591 (0.8335, 1.1038)	−0.0426 (−0.1998, 0.094)	−0.0224 (−0.1008, 0.0504)	0.3388	0.5605	1.25	NA
Congenital posterior urethral valves	138	857,092	685	4,036,200	0.9487 (0.7901, 1.1391)	−0.0541 (−0.2655, 0.1221)	−0.0091 (−0.0405, 0.0214)	0.3184	0.5725	1.29	NA
Interrupted aortic arch	86	1,397,634	247	3,744,641	0.9329 (0.7299, 1.1923)	−0.072 (−0.3701, 0.1613)	−0.0186 (−0.0852, 0.044)	0.3082	0.5788	1.35	NA
Dextro-transposition of great arteries (d-TGA)	710	3,124,610	928	3,803,093	0.9312 (0.8445, 1.0268)	−0.0738 (−0.1841, 0.0261)	−0.032 (−0.0767, 0.0108)	2.0431	0.1529	1.36	NA
Congenital cataract	718	5,120,845	664	4,243,840	0.8961 (0.8064, 0.9959)	−0.1159 (−0.2401, −0.0041)	−0.0602 (−0.1199, −0.0036)	4.1522	0.0416	1.48	NA
Bladder exstrophy	105	5,028,475	92	3,896,531	0.8844 (0.6685, 1.17)	−0.1307 (−0.4959, 0.1453)	−0.0697 (−0.2418, 0.0786)	0.7411	0.3893	1.52	NA
Cleft palate alone	2399	4,579,165	2605	4,360,721	0.877 (0.8297, 0.927)	−0.1402 (−0.2052, −0.0787)	−0.0672 (−0.096, −0.0392)	21.5360	3.47E-06	1.54	NA
Pyloric stenosis	3934	2,242,962	192	95,545	0.8728 (0.755, 1.009)	−0.1454 (−0.3238, 0.0089)	−0.1387 (−0.3071, 0.0081)	3.3864	0.0657	1.55	NA
Pulmonary valve atresia and stenosis	3896	5,109,183	3880	4,359,446	0.8568 (0.8195, 0.8957)	−0.167 (−0.22, −0.1163)	−0.0837 (−0.1081, −0.0598)	46.5051	9.14E-12	1.61	NA
Limb deficiencies (reduction defects)	558	1,485,079	1830	4,142,972	0.8506 (0.7737, 0.9352)	−0.1755 (−0.2924, −0.0692)	−0.041 (−0.0643, −0.0182)	11.2100	8.14E-04	1.63	NA
Hydrocephalus without spina bifida	2149	3,618,806	68	95,669	0.8355 (0.6562, 1.0637)	−0.1968 (−0.5234, 0.0598)	−0.1907 (−0.5046, 0.0576)	2.1340	0.1441	1.68	NA
Amniotic Bands	270	2,585,899	12	95,725	0.8329 (0.4672, 1.485)	−0.2006 (−1.1404, 0.3266)	−0.1921 (−1.0736, 0.3147)	0.3851	0.5349	1.69	NA
Ebstein anomaly	334	5,107,133	323	4,105,245	0.8312 (0.7133, 0.9686)	−0.2031 (−0.4019, −0.0324)	−0.1032 (−0.1924, −0.0207)	5.6284	0.0177	1.70	NA
Choanal atresia	521	5,096,396	542	4,311,319	0.8132 (0.721, 0.9171)	−0.2297 (−0.3868, −0.0904)	−0.1126 (−0.1801, −0.0489)	11.4006	7.34E-04	1.76	NA
Omphalocele	759	4,673,203	785	3,901,285	0.8072 (0.7305, 0.8919)	−0.2388 (−0.3688, −0.1212)	−0.1174 (−0.1736, −0.0639)	17.7733	2.49E-05	1.78	NA
Common truncus (truncus arteriosus)	278	4,620,690	336	4,240,246	0.7593 (0.6477, 0.89)	−0.317 (−0.5439, −0.1235)	−0.1435 (−0.2289, −0.0642)	11.6117	6.55E-04	1.96	NA
Reduction deformity, Lower limbs	537	3,780,505	18	95,719	0.7554 (0.4722, 1.2082)	−0.3238 (−1.1173, 0.1723)	−0.3133 (−1.0687, 0.1662)	1.3798	0.2401	1.98	NA
Ventricular septal defect	14,557	3,929,002	21,430	4,341,896	0.7507 (0.735, 0.7667)	−0.3305 (−0.3588, −0.3029)	−0.1337 (−0.1434, −0.1241)	714.8336	2.2e-320	1.99	NA
Craniosynostosis	470	1,120,061	1679	2,992,172	0.7478 (0.6751, 0.8284)	−0.337 (−0.481, −0.2071)	−0.0737 (−0.098, −0.05)	31.2156	2.31E-08	2.01	NA
Hypospadias	24,587	5,157,522	27,515	4,196,875	0.7271 (0.7147, 0.7398)	−0.3728 (−0.3965, −0.3495)	−0.1759 (−0.1855, −0.1664)	1321.8209	1.02E-289	2.09	NA
Anophthalmia/microphthalmia	561	5,108,305	636	4,192,403	0.7239 (0.6462, 0.811)	−0.3813 (−0.5474, −0.2331)	−0.1787 (−0.2431, −0.1176)	31.3790	2.12E-08	2.11	NA
Turner syndrome	125	845,663	838	4,079,070	0.7195 (0.5962, 0.8683)	−0.3898 (−0.6771, −0.1517)	−0.0506 (−0.0765, −0.0253)	11.8932	5.63E-04	2.13	NA
Microcephalus	1436	2,490,665	81	95,656	0.6809 (0.5443, 0.8518)	−0.4683 (−0.8365, −0.1739)	−0.4433 (−0.7838, −0.1678)	11.4591	7.11E-04	2.30	NA
Holoprosencephaly	198	1,372,610	882	3,954,496	0.6468 (0.5544, 0.7545)	−0.5461 (−0.8037, −0.3252)	−0.1001 (−0.1316, −0.0695)	31.1923	2.34E-08	2.46	NA
Epispadias	230	2,250,082	41	257,008	0.6408 (0.4596, 0.8933)	−0.5606 (−1.1756, −0.1194)	−0.4758 (−0.9565, −0.1132)	7.0075	0.0081	2.50	NA
Renal agenesis/hypoplasia	1586	5,011,917	2240	4,361,086	0.6161 (0.5777, 0.657)	−0.6228 (−0.7306, −0.5217)	−0.2582 (−0.2922, −0.2251)	222.0395	1.63E-50	2.63	NA
Atrial septal defect	20,411	5,016,582	33,798	4,329,528	0.5212 (0.5122, 0.5304)	−0.9115 (−0.9449, −0.8787)	−0.3432 (−0.352, −0.3345)	5563.5773	2.2e-320	3.23	NA
Reduction deformity, Upper limbs	1001	3,765,890	50	95,687	0.5087 (0.3829, 0.6758)	−0.9653 (−1.6107, −0.4795)	−0.9194 (−1.5155, −0.4646)	22.5864	2.01E-06	3.34	NA
Tricuspid valve atresia and stenosis	510	5,059,825	973	4,362,353	0.4519 (0.406, 0.503)	−1.2126 (−1.4628, −0.9878)	−0.417 (−0.4702, −0.3658)	222.4058	1.36E-50	3.85	NA

As shown in Table 4 six cardiovascular anomalies, five chromosomal, five gastrointestinal, two urinary, two limb, and one each facial (Holoprosencephaly), body wall (Diaphragmatic hernia) and CNS (spina bifida without anencephalus) anomaly are accompanied by higher E-Values in the high cannabis use quintiles. Interestingly both congenital posterior urethral vales and diaphragmatic hernia and several gastrointestinal anomalies appear both on this list and on the list of elevated E-Values shown in Table 2 where cannabis exposure is treated as a continuous covariate.

As indicated in Table 5 12 anomalies including three cardiovascular (pulmonary valve atresia, double outlet right ventricle, single ventricle), three gastrointestinal (small intestinal atresia /stenosis, biliary atresia, cloacal extrophy), two chromosomal (Trisomies 14 and 21) and one each limb (clubfoot), body wall (diaphragmatic hernia), face (cleft lip with and without cleft palate) and genitourinary (obstructive genitourinary defect) anomaly were noted to have elevated minimum E-Values in highest cannabidiol exposure quintiles.

For ease of comparison these Prevalence Ratios are presented together by substance in Table 6. The prevalence ratios for cannabidiol appear in the right hand column and are listed in descending order.

**Table 6:** Prevalence Ratios by Substance

Congenital Anomaly	Cigarettes Prevalence Ratio	Binge Alcohol Prevalence Ratio	Analgesics Prevalence Ratio	Ccoaine Prevalence Ratio	Cannabis Prevalence Ratio	Cannabidiol Prevalence Ratio
Obstructive genitourinary defect	0.92 (0.87, 0.97)	1.02 (0.97, 1.07)	0.9 (0.85, 0.94)	1.17 (1.11, 1.23)	0.86 (0.82, 0.9)	1.92 (1.63, 2.27)
Cleft lip with and without cleft palate	1.06 (0.98, 1.13)	0.95 (0.88, 1.02)	1.3 (1.2, 1.41)	1.02 (0.94, 1.11)	0.94 (0.87, 1.01)	1.52 (1.08, 2.14)
Pulmonary valve atresia	1.06 (0.91, 1.22)	0.45 (0.38, 0.54)	1.64 (1.46, 1.85)	0.97 (0.86, 1.1)	1.27 (1.14, 1.43)	1.35 (1.18, 1.55)
Cloacal exstrophy	2.84 (2.44, 3.31)	0.85 (0.73, 0.98)	1.61 (1.41, 1.83)	0.63 (0.54, 0.72)	4.85 (4.08, 5.77)	1.3 (1.12, 1.51)
Hirschsprung disease (congenital megacolon)	1.12 (0.95, 1.33)	0.57 (0.47, 0.7)	1.06 (0.89, 1.27)	1.01 (0.84, 1.21)	1.46 (1.24, 1.72)	1.29 (0.8, 2.09)
Congenital hip dislocation	0.93 (0.84, 1.04)	1.09 (0.97, 1.24)	0.95 (0.85, 1.06)	1.85 (1.65, 2.07)	2.28 (2.08, 2.51)	1.28 (0.87, 1.87)
Small intestinal atresia/stenosis	0.86 (0.77, 0.97)	0.87 (0.77, 0.97)	1.09 (0.99, 1.2)	1.1 (1, 1.21)	1.22 (1.12, 1.33)	1.26 (1.14, 1.39)
Single ventricle	0.84 (0.66, 1.05)	0.46 (0.36, 0.59)	1.07 (0.93, 1.23)	0.81 (0.7, 0.93)	1.21 (1.06, 1.39)	1.22 (1, 1.5)
Deletion 22q11.2	0.59 (0.45, 0.77)	1.26 (0.98, 1.6)	2.93 (2.39, 3.58)	1.81 (1.46, 2.25)	1.36 (1.09, 1.68)	1.21 (0.9, 1.64)
Biliary atresia	1.02 (0.87, 1.2)	0.63 (0.51, 0.77)	0.88 (0.74, 1.05)	1.12 (0.94, 1.32)	1.19 (1.02, 1.39)	1.2 (1.02, 1.4)
Double outlet right ventricle	1.04 (0.92, 1.17)	0.69 (0.6, 0.79)	1.12 (1.01, 1.23)	0.91 (0.82, 1.01)	1.19 (1.08, 1.31)	1.16 (1.01, 1.33)
Trisomy 13	0.62 (0.53, 0.71)	0.97 (0.85, 1.1)	0.86 (0.82, 0.9)	1.76 (1.67, 1.85)	1.29 (1.23, 1.35)	1.14 (1, 1.28)
Total anomalous pulmonary venous connection	0.62 (0.52, 0.74)	0.62 (0.5, 0.75)	1.44 (1.24, 1.66)	1.31 (1.13, 1.52)	1.05 (0.93, 1.19)	1.13 (0.99, 1.29)
Clubfoot	1.02 (0.97, 1.08)	0.88 (0.83, 0.93)	1.03 (0.97, 1.1)	0.99 (0.94, 1.05)	1.07 (1.01, 1.14)	1.1 (1.03, 1.18)
Diaphragmatic hernia	1.22 (1.13, 1.33)	0.83 (0.76, 0.91)	1.15 (1.06, 1.26)	0.87 (0.79, 0.95)	1.24 (1.15, 1.34)	1.09 (1, 1.17)
Patent ductus arteriosus	0.96 (0.92, 1)	0.72 (0.69, 0.75)	1.13 (1.08, 1.18)	0.79 (0.75, 0.82)	0.58 (0.56, 0.6)	1.08 (0.89, 1.3)
Trisomy 21 (Down syndrome)	0.83 (0.8, 0.87)	1.03 (0.99, 1.07)	1.02 (0.99, 1.04)	1.13 (1.11, 1.16)	1.14 (1.12, 1.17)	1.06 (1.02, 1.09)
Atrioventricular septal defect	0.95 (0.89, 1.01)	0.79 (0.74, 0.84)	1.07 (1.01, 1.13)	0.92 (0.87, 0.98)	1.05 (1, 1.11)	1.06 (0.99, 1.12)
Trisomy 18	0.66 (0.6, 0.73)	0.89 (0.82, 0.98)	1.06 (1.02, 1.1)	1.34 (1.29, 1.39)	1.31 (1.27, 1.35)	1.04 (0.96, 1.13)
Cleft lip alone	1.06 (0.96, 1.17)	1.04 (0.94, 1.15)	1.18 (1.08, 1.28)	0.82 (0.75, 0.89)	0.97 (0.9, 1.05)	1.03 (0.93, 1.14)
Hypoplastic left heart syndrome	1.2 (1.1, 1.3)	0.69 (0.62, 0.75)	1.12 (1.04, 1.19)	0.91 (0.85, 0.98)	1.1 (1.03, 1.17)	1.03 (0.95, 1.11)
Aortic valve stenosis	0.96 (0.87, 1.06)	0.71 (0.64, 0.8)	1.59 (1.48, 1.71)	1.03 (0.96, 1.11)	0.9 (0.84, 0.96)	1.02 (0.93, 1.12)
Transposition of great arteries	1.25 (1.16, 1.36)	0.8 (0.73, 0.87)	1.23 (1.14, 1.32)	0.85 (0.79, 0.91)	1.01 (0.94, 1.09)	1.01 (0.94, 1.09)
Anotia/microtia	0.37 (0.32, 0.42)	0.87 (0.77, 0.97)	1.38 (1.3, 1.46)	1.62 (1.53, 1.71)	1 (0.96, 1.05)	1.01 (0.92, 1.1)
Rectal and large intestinal atresia/stenosis	1.2 (1.12, 1.28)	0.84 (0.78, 0.9)	1.08 (1, 1.16)	0.87 (0.81, 0.94)	0.9 (0.85, 0.96)	1 (0.94, 1.07)
Spina bifida without anencephalus	1.04 (0.97, 1.11)	0.83 (0.77, 0.9)	1.36 (1.3, 1.42)	1 (0.96, 1.05)	1.05 (1.01, 1.09)	1 (0.94, 1.07)
Gastroschisis	1 (0.94, 1.07)	0.89 (0.83, 0.96)	1.47 (1.39, 1.56)	0.97 (0.91, 1.03)	0.93 (0.88, 0.98)	1 (0.94, 1.06)
Cleft lip with cleft palate	1.14 (1.06, 1.22)	0.86 (0.79, 0.93)	1.37 (1.28, 1.46)	0.93 (0.87, 0.99)	0.96 (0.9, 1.02)	0.99 (0.92, 1.06)
Coarctation of the aorta	1.16 (1.09, 1.23)	0.7 (0.66, 0.75)	1.16 (1.1, 1.22)	0.87 (0.82, 0.92)	1.38 (1.32, 1.45)	0.98 (0.93, 1.04)
Anencephalus	0.89 (0.81, 0.99)	0.7 (0.63, 0.78)	1.36 (1.29, 1.42)	1.03 (0.99, 1.08)	0.93 (0.89, 0.96)	0.97 (0.88, 1.07)
Esophageal atresia/tracheoesophageal fistula	1.08 (0.99, 1.18)	1.06 (0.97, 1.16)	1.04 (0.95, 1.14)	1.01 (0.92, 1.1)	1.11 (1.02, 1.21)	0.97 (0.89, 1.05)
Tetralogy of Fallot	1.1 (1.03, 1.17)	0.78 (0.73, 0.84)	1.03 (0.97, 1.1)	0.99 (0.93, 1.05)	0.96 (0.91, 1.02)	0.96 (0.91, 1.03)
Encephalocele	1.21 (1.05, 1.39)	0.68 (0.58, 0.81)	1.08 (0.98, 1.18)	0.91 (0.83, 1)	0.94 (0.86, 1.02)	0.96 (0.83, 1.1)
Congenital posterior urethral valves	1.11 (0.95, 1.29)	0.61 (0.52, 0.71)	1.06 (0.92, 1.23)	0.84 (0.73, 0.97)	1.33 (1.15, 1.54)	0.95 (0.79, 1.14)
Interrupted aortic arch	1.27 (0.99, 1.62)	0.85 (0.65, 1.11)	1.38 (1.12, 1.69)	0.81 (0.65, 1)	1.04 (0.84, 1.27)	0.93 (0.73, 1.19)
Dextro-transposition of great arteries (d-TGA)	1.19 (1.08, 1.33)	0.89 (0.79, 1)	0.96 (0.87, 1.07)	0.89 (0.8, 0.98)	0.75 (0.68, 0.82)	0.93 (0.84, 1.03)
Congenital cataract	0.93 (0.83, 1.04)	0.91 (0.81, 1.02)	1.09 (0.97, 1.22)	0.97 (0.87, 1.08)	0.94 (0.86, 1.04)	0.9 (0.81, 1)
Bladder exstrophy	1.57 (1.19, 2.05)	0.92 (0.69, 1.24)	1.16 (0.86, 1.57)	0.86 (0.62, 1.18)	1.03 (0.75, 1.4)	0.88 (0.67, 1.17)
Cleft palate alone	1.23 (1.16, 1.31)	1.01 (0.95, 1.08)	1.16 (1.09, 1.22)	0.97 (0.91, 1.02)	0.96 (0.91, 1.01)	0.88 (0.83, 0.93)
Pyloric stenosis	1.72 (1.63, 1.82)	0.35 (0.32, 0.38)	1.9 (1.8, 2.01)	0.66 (0.62, 0.71)	0.89 (0.84, 0.95)	0.87 (0.75, 1.01)
Pulmonary valve atresia and stenosis	1 (0.95, 1.05)	0.71 (0.67, 0.75)	1.02 (0.97, 1.07)	0.87 (0.83, 0.92)	0.88 (0.84, 0.92)	0.86 (0.82, 0.9)
Limb deficiencies (reduction defects)	1.09 (1, 1.19)	0.87 (0.79, 0.95)	1.07 (1, 1.15)	0.85 (0.79, 0.91)	0.77 (0.73, 0.82)	0.85 (0.77, 0.94)
Hydrocephalus without spina bifida	1.34 (1.22, 1.47)	1.03 (0.94, 1.14)	0.87 (0.8, 0.95)	0.97 (0.89, 1.06)	1.05 (0.97, 1.12)	0.84 (0.66, 1.06)
Amniotic Bands	0.95 (0.67, 1.33)	0.65 (0.49, 0.86)	1.49 (1.04, 2.13)	0.67 (0.44, 1.03)	0.84 (0.58, 1.23)	0.83 (0.47, 1.49)
Ebstein anomaly	1.13 (0.97, 1.33)	0.71 (0.59, 0.85)	1.33 (1.16, 1.53)	0.92 (0.8, 1.05)	0.88 (0.78, 1.01)	0.83 (0.71, 0.97)
Choanal atresia	1.4 (1.23, 1.58)	1.02 (0.9, 1.17)	0.83 (0.74, 0.93)	0.88 (0.78, 0.99)	0.78 (0.69, 0.87)	0.81 (0.72, 0.92)
Omphalocele	1.19 (1.07, 1.31)	0.79 (0.71, 0.87)	1.07 (1.01, 1.15)	0.85 (0.79, 0.91)	0.92 (0.87, 0.98)	0.81 (0.73, 0.89)
Common truncus (truncus arteriosus)	2.1 (1.8, 2.46)	0.58 (0.48, 0.7)	1.02 (0.87, 1.2)	0.72 (0.61, 0.85)	0.73 (0.63, 0.84)	0.76 (0.65, 0.89)
Reduction deformity, Lower limbs	1.22 (1.03, 1.45)	0.94 (0.78, 1.13)	1.04 (0.88, 1.24)	0.82 (0.69, 0.98)	0.84 (0.72, 0.98)	0.76 (0.47, 1.21)
Ventricular septal defect	1.19 (1.17, 1.22)	0.84 (0.82, 0.86)	0.85 (0.83, 0.87)	0.79 (0.77, 0.81)	0.74 (0.73, 0.76)	0.75 (0.73, 0.77)
Craniosynostosis	1.27 (1.14, 1.42)	0.61 (0.54, 0.69)	1.12 (1.01, 1.24)	0.98 (0.89, 1.08)	0.54 (0.5, 0.59)	0.75 (0.68, 0.83)
Hypospadias	1.59 (1.56, 1.62)	0.87 (0.85, 0.89)	0.98 (0.96, 1)	1 (0.98, 1.02)	0.74 (0.72, 0.75)	0.73 (0.71, 0.74)
Anophthalmia/microphthalmia	0.79 (0.69, 0.91)	1.03 (0.9, 1.18)	1.18 (1.08, 1.27)	1.17 (1.07, 1.27)	0.59 (0.55, 0.63)	0.72 (0.65, 0.81)
Turner syndrome	0.61 (0.53, 0.71)	1.14 (1.01, 1.3)	1.01 (0.95, 1.08)	1.11 (1.04, 1.19)	1.57 (1.47, 1.66)	0.72 (0.6, 0.87)
Microcephalus	1.32 (1.18, 1.46)	0.78 (0.7, 0.87)	1.1 (0.98, 1.23)	0.91 (0.82, 1.02)	0.71 (0.65, 0.78)	0.68 (0.54, 0.85)
Holoprosencephaly	1.92 (1.71, 2.16)	0.56 (0.49, 0.65)	0.85 (0.8, 0.9)	0.39 (0.36, 0.41)	1.24 (1.17, 1.31)	0.65 (0.55, 0.75)
Epispadias	0.8 (0.62, 1.04)	1.22 (0.92, 1.62)	0.69 (0.53, 0.91)	1.37 (1.04, 1.8)	1.31 (1.03, 1.67)	0.64 (0.46, 0.89)
Renal agenesis/hypoplasia	1.25 (1.17, 1.34)	0.92 (0.86, 0.99)	0.95 (0.9, 1)	0.84 (0.8, 0.89)	0.54 (0.51, 0.57)	0.62 (0.58, 0.66)
Atrial septal defect	2.53 (2.49, 2.57)	0.56 (0.54, 0.57)	1.31 (1.29, 1.34)	0.71 (0.7, 0.73)	0.71 (0.7, 0.72)	0.52 (0.51, 0.53)
Reduction deformity, Upper limbs	0.9 (0.79, 1.03)	0.95 (0.83, 1.09)	1.02 (0.9, 1.16)	0.83 (0.73, 0.94)	0.7 (0.63, 0.79)	0.51 (0.38, 0.68)
Tricuspid valve atresia and stenosis	0.67 (0.59, 0.76)	0.91 (0.81, 1.03)	0.61 (0.56, 0.68)	1.17 (1.07, 1.29)	0.53 (0.49, 0.58)	0.45 (0.41, 0.5)
Aniridia	1.24 (0.65, 2.38)	0.34 (0.15, 0.78)	1.84 (1.29, 2.63)	1.72 (1.14, 2.6)	1.45 (0.94, 2.24)	–

Table 7 presents the Attributable Fractions in the Exposed (AFEs) in a similar manner. One notes that they descend from a strikingly high rate of 79.38% for cloacal extrophy after cannabis exposure.

**Table 7:** Attributable Fraction in the Exposed by Substance

Congenital Anomaly	Cigarettes AFE	Binge Alcohol AFE	Analgesics AFE	Cocaine AFE	Cannabis AFE	Cannabidiol AFE
Obstructive genitourinary defect	−0.09 (−0.15, −0.03)	0.02 (−0.03, 0.07)	−0.11 (−0.17, −0.06)	0.15 (0.1, 0.19)	−0.16 (−0.22, −0.12)	0.48 (0.39, 0.56)
Cleft lip with and without cleft palate	0.05 (−0.02, 0.12)	−0.06 (−0.14, 0.02)	0.23 (0.17, 0.29)	0.02 (−0.06, 0.1)	−0.07 (−0.15, 0.01)	0.34 (0.07, 0.53)
Pulmonary valve atresia	0.05 (−0.09, 0.18)	−1.21 (−1.63, −0.86)	0.39 (0.32, 0.46)	−0.03 (−0.16, 0.09)	0.22 (0.12, 0.3)	0.26 (0.15, 0.35)
Cloacal exstrophy	0.65 (0.59, 0.7)	−0.18 (−0.36, −0.02)	0.38 (0.29, 0.45)	−0.6 (−0.84, −0.39)	0.79 (0.75, 0.83)	0.23 (0.11, 0.34)
Hirschsprung disease (congenital megacolon)	0.11 (−0.06, 0.25)	−0.75 (−1.12, −0.44)	0.06 (−0.12, 0.21)	0.01 (−0.2, 0.17)	0.31 (0.19, 0.42)	0.22 (−0.26, 0.52)
Congenital hip dislocation	−0.07 (−0.19, 0.04)	0.09 (−0.03, 0.19)	−0.05 (−0.18, 0.06)	0.46 (0.4, 0.52)	0.56 (0.52, 0.6)	0.22 (−0.15, 0.46)
Small intestinal atresia/stenosis	−0.16 (−0.29, −0.03)	−0.15 (−0.29, −0.03)	0.08 (−0.01, 0.16)	0.09 (0, 0.17)	0.18 (0.11, 0.25)	0.21 (0.12, 0.28)
Single ventricle	−0.2 (−0.51, 0.05)	−1.17 (−1.77, −0.7)	0.06 (−0.08, 0.19)	−0.24 (−0.43, −0.07)	0.18 (0.05, 0.28)	0.18 (0, 0.33)
Deletion 22q11.2	−0.69 (−1.2, −0.3)	0.2 (−0.02, 0.38)	0.66 (0.58, 0.72)	0.45 (0.31, 0.56)	0.26 (0.09, 0.41)	0.18 (−0.11, 0.39)
Biliary atresia	0.02 (−0.15, 0.17)	−0.6 (−0.96, −0.31)	−0.13 (−0.34, 0.05)	0.1 (−0.06, 0.24)	0.16 (0.02, 0.28)	0.17 (0.02, 0.29)
Double outlet right ventricle	0.03 (−0.09, 0.15)	−0.46 (−0.67, −0.27)	0.1 (0.01, 0.19)	−0.1 (−0.21, 0.01)	0.16 (0.07, 0.24)	0.13 (0.01, 0.25)
Trisomy 13	−0.62 (−0.87, −0.41)	−0.04 (−0.18, 0.09)	−0.16 (−0.23, −0.11)	0.43 (0.4, 0.46)	0.22 (0.18, 0.26)	0.12 (0, 0.22)
Total anomalous pulmonary venous connection	−0.6 (−0.91, −0.34)	−0.62 (−0.98, −0.33)	0.3 (0.19, 0.4)	0.24 (0.11, 0.34)	0.05 (−0.08, 0.16)	0.11 (−0.01, 0.23)
Clubfoot	0.02 (−0.04, 0.07)	−0.14 (−0.2, −0.08)	0.03 (−0.03, 0.09)	−0.01 (−0.07, 0.05)	0.07 (0.01, 0.12)	0.09 (0.03, 0.15)
Diaphragmatic hernia	0.18 (0.12, 0.25)	−0.21 (−0.32, −0.1)	0.13 (0.05, 0.2)	−0.15 (−0.26, −0.06)	0.2 (0.13, 0.26)	0.08 (0, 0.15)
Patent ductus arteriosus	−0.05 (−0.09, 0)	−0.39 (−0.45, −0.33)	0.11 (0.07, 0.15)	−0.27 (−0.33, −0.21)	−0.72 (−0.79, −0.65)	0.07 (−0.12, 0.23)
Trisomy 21 (Down syndrome)	−0.2 (−0.25, −0.15)	0.03 (−0.01, 0.07)	0.02 (−0.01, 0.04)	0.12 (0.1, 0.14)	0.12 (0.11, 0.14)	0.05 (0.02, 0.09)
Atrioventricular septal defect	−0.06 (−0.13, 0.01)	−0.27 (−0.36, −0.18)	0.06 (0.01, 0.12)	−0.08 (−0.15, −0.02)	0.05 (0, 0.1)	0.05 (−0.01, 0.11)
Trisomy 18	−0.52 (−0.67, −0.38)	−0.12 (−0.22, −0.02)	0.05 (0.02, 0.09)	0.25 (0.23, 0.28)	0.24 (0.21, 0.26)	0.04 (−0.04, 0.12)
Cleft lip alone	0.06 (−0.04, 0.15)	0.04 (−0.07, 0.13)	0.15 (0.07, 0.22)	−0.23 (−0.33, −0.13)	−0.03 (−0.12, 0.04)	0.03 (−0.08, 0.12)
Hypoplastic left heart syndrome	0.17 (0.09, 0.23)	−0.46 (−0.61, −0.32)	0.1 (0.04, 0.16)	−0.1 (−0.18, −0.03)	0.09 (0.03, 0.15)	0.03 (−0.06, 0.1)
Aortic valve stenosis	−0.04 (−0.16, 0.06)	−0.4 (−0.57, −0.25)	0.37 (0.32, 0.42)	0.03 (−0.04, 0.1)	−0.12 (−0.19, −0.04)	0.02 (−0.07, 0.11)
Transposition of great arteries	0.2 (0.14, 0.26)	−0.25 (−0.36, −0.14)	0.18 (0.12, 0.24)	−0.18 (−0.27, −0.09)	0.01 (−0.06, 0.08)	0.01 (−0.06, 0.08)
Anotia/microtia	−1.73 (−2.1, −1.4)	−0.15 (−0.29, −0.03)	0.28 (0.23, 0.32)	0.38 (0.35, 0.42)	0 (−0.04, 0.04)	0.01 (−0.09, 0.09)
Rectal and large intestinal atresia/stenosis	0.17 (0.11, 0.22)	−0.19 (−0.28, −0.11)	0.07 (0, 0.14)	−0.15 (−0.24, −0.07)	−0.11 (−0.18, −0.04)	0 (−0.06, 0.07)
Spina bifida without anencephalus	0.04 (−0.03, 0.1)	−0.2 (−0.3, −0.11)	0.27 (0.23, 0.3)	0 (−0.05, 0.04)	0.05 (0.01, 0.08)	0 (−0.07, 0.07)
Gastroschisis	0 (−0.07, 0.06)	−0.13 (−0.21, −0.05)	0.32 (0.28, 0.36)	−0.03 (−0.1, 0.03)	−0.08 (−0.14, −0.02)	0 (−0.07, 0.06)
Cleft lip with cleft palate	0.12 (0.06, 0.18)	−0.16 (−0.26, −0.07)	0.27 (0.22, 0.32)	−0.08 (−0.15, −0.01)	−0.05 (−0.11, 0.02)	−0.01 (−0.08, 0.06)
Coarctation of the aorta	0.14 (0.09, 0.19)	−0.42 (−0.52, −0.33)	0.14 (0.09, 0.18)	−0.15 (−0.21, −0.09)	0.28 (0.24, 0.31)	−0.02 (−0.08, 0.04)
Anencephalus	−0.12 (−0.24, −0.01)	−0.43 (−0.59, −0.28)	0.26 (0.23, 0.3)	0.03 (−0.01, 0.08)	−0.08 (−0.12, −0.04)	−0.03 (−0.14, 0.06)
Esophageal atresia/tracheoesophageal fistula	0.07 (−0.01, 0.15)	0.06 (−0.03, 0.14)	0.04 (−0.05, 0.12)	0.01 (−0.09, 0.09)	0.1 (0.02, 0.17)	−0.03 (−0.12, 0.05)
Tetralogy of Fallot	0.09 (0.03, 0.15)	−0.28 (−0.38, −0.19)	0.03 (−0.03, 0.09)	−0.01 (−0.08, 0.05)	−0.04 (−0.1, 0.02)	−0.04 (−0.1, 0.03)
Encephalocele	0.17 (0.05, 0.28)	−0.46 (−0.72, −0.24)	0.07 (−0.02, 0.15)	−0.1 (−0.2, 0)	−0.07 (−0.16, 0.02)	−0.04 (−0.2, 0.09)
Congenital posterior urethral valves	0.1 (−0.06, 0.23)	−0.65 (−0.93, −0.42)	0.06 (−0.08, 0.18)	−0.19 (−0.38, −0.03)	0.25 (0.13, 0.35)	−0.05 (−0.27, 0.12)
Interrupted aortic arch	0.21 (−0.01, 0.38)	−0.17 (−0.54, 0.1)	0.27 (0.11, 0.41)	−0.24 (−0.53, 0)	0.04 (−0.18, 0.21)	−0.07 (−0.37, 0.16)
Dextro-transposition of great arteries (d-TGA)	0.16 (0.07, 0.25)	−0.13 (−0.27, 0)	−0.04 (−0.15, 0.06)	−0.13 (−0.25, −0.02)	−0.34 (−0.46, −0.22)	−0.07 (−0.18, 0.03)
Congenital cataract	−0.07 (−0.2, 0.04)	−0.1 (−0.24, 0.02)	0.08 (−0.03, 0.18)	−0.03 (−0.15, 0.08)	−0.06 (−0.17, 0.04)	−0.12 (−0.24, 0)
Bladder exstrophy	0.36 (0.16, 0.51)	−0.08 (−0.45, 0.19)	0.14 (−0.16, 0.36)	−0.17 (−0.61, 0.15)	0.03 (−0.33, 0.29)	−0.13 (−0.5, 0.15)
Cleft palate alone	0.19 (0.14, 0.23)	0.01 (−0.05, 0.07)	0.13 (0.08, 0.18)	−0.04 (−0.1, 0.02)	−0.05 (−0.1, 0.01)	−0.14 (−0.21, −0.08)
Pyloric stenosis	0.42 (0.39, 0.45)	−1.85 (−2.08, −1.63)	0.47 (0.44, 0.5)	−0.5 (−0.61, −0.4)	−0.12 (−0.19, −0.05)	−0.15 (−0.32, 0.01)
Pulmonary valve atresia and stenosis	0 (−0.05, 0.05)	−0.4 (−0.48, −0.33)	0.02 (−0.03, 0.06)	−0.14 (−0.2, −0.09)	−0.14 (−0.19, −0.09)	−0.17 (−0.22, −0.12)
Limb deficiencies (reduction defects)	0.09 (0, 0.16)	−0.15 (−0.26, −0.05)	0.07 (0, 0.13)	−0.18 (−0.26, −0.1)	−0.29 (−0.37, −0.22)	−0.18 (−0.29, −0.07)
Hydrocephalus without spina bifida	0.25 (0.18, 0.32)	0.03 (−0.06, 0.12)	−0.15 (−0.25, −0.06)	−0.03 (−0.12, 0.05)	0.04 (−0.03, 0.11)	−0.2 (−0.52, 0.06)
Amniotic Bands	−0.06 (−0.49, 0.25)	−0.53 (−1.03, −0.16)	0.33 (0.04, 0.53)	−0.49 (−1.28, 0.03)	−0.19 (−0.73, 0.19)	−0.2 (−1.14, 0.33)
Ebstein anomaly	0.12 (−0.03, 0.25)	−0.41 (−0.69, −0.18)	0.25 (0.14, 0.35)	−0.09 (−0.25, 0.05)	−0.13 (−0.29, 0.01)	−0.2 (−0.4, −0.03)
Choanal atresia	0.28 (0.19, 0.37)	0.02 (−0.11, 0.15)	−0.2 (−0.35, −0.07)	−0.14 (−0.28, −0.01)	−0.29 (−0.45, −0.15)	−0.23 (−0.39, −0.09)
Omphalocele	0.16 (0.07, 0.24)	−0.27 (−0.41, −0.14)	0.07 (0.01, 0.13)	−0.18 (−0.26, −0.1)	−0.09 (−0.15, −0.02)	−0.24 (−0.37, −0.12)
Common truncus (truncus arteriosus)	0.52 (0.44, 0.59)	−0.73 (−1.1, −0.43)	0.02 (−0.15, 0.16)	−0.39 (−0.64, −0.18)	−0.37 (−0.59, −0.18)	−0.32 (−0.54, −0.12)
Reduction deformity, Lower limbs	0.18 (0.03, 0.31)	−0.07 (−0.28, 0.12)	0.04 (−0.14, 0.19)	−0.22 (−0.46, −0.02)	−0.2 (−0.4, −0.02)	−0.32 (−1.12, 0.17)
Ventricular septal defect	0.16 (0.14, 0.18)	−0.19 (−0.22, −0.16)	−0.18 (−0.2, −0.15)	−0.26 (−0.29, −0.23)	−0.34 (−0.37, −0.31)	−0.33 (−0.36, −0.3)
Craniosynostosis	0.21 (0.12, 0.29)	−0.64 (−0.84, −0.46)	0.11 (0.01, 0.19)	−0.02 (−0.13, 0.08)	−0.84 (−1.01, −0.69)	−0.34 (−0.48, −0.21)
Hypospadias	0.37 (0.36, 0.38)	−0.15 (−0.17, −0.13)	−0.02 (−0.04, 0)	0 (−0.02, 0.02)	−0.35 (−0.38, −0.33)	−0.37 (−0.4, −0.35)
Anophthalmia/microphthalmia	−0.26 (−0.44, −0.1)	0.03 (−0.11, 0.15)	0.15 (0.08, 0.22)	0.14 (0.07, 0.21)	−0.71 (−0.83, −0.59)	−0.38 (−0.55, −0.23)
Turner syndrome	−0.63 (−0.89, −0.41)	0.13 (0.01, 0.23)	0.01 (−0.05, 0.07)	0.1 (0.04, 0.16)	0.36 (0.32, 0.4)	−0.39 (−0.68, −0.15)
Microcephalus	0.24 (0.16, 0.32)	−0.28 (−0.42, −0.15)	0.09 (−0.02, 0.18)	−0.09 (−0.22, 0.02)	−0.41 (−0.54, −0.28)	−0.47 (−0.84, −0.17)
Holoprosencephaly	0.48 (0.41, 0.54)	−0.78 (−1.05, −0.54)	−0.18 (−0.25, −0.11)	−1.58 (−1.74, −1.43)	0.19 (0.14, 0.24)	−0.55 (−0.8, −0.33)
Epispadias	−0.24 (−0.6, 0.03)	0.18 (−0.08, 0.38)	−0.44 (−0.9, −0.1)	0.27 (0.04, 0.44)	0.24 (0.03, 0.4)	−0.56 (−1.18, −0.12)
Renal agenesis/hypoplasia	0.2 (0.15, 0.25)	−0.08 (−0.16, −0.01)	−0.05 (−0.11, 0)	−0.18 (−0.25, −0.12)	−0.86 (−0.97, −0.76)	−0.62 (−0.73, −0.52)
Atrial septal defect	0.6 (0.6, 0.61)	−0.79 (−0.83, −0.76)	0.24 (0.22, 0.25)	−0.4 (−0.43, −0.37)	−0.4 (−0.43, −0.38)	−0.91 (−0.94, −0.88)
Reduction deformity, Upper limbs	−0.11 (−0.27, 0.03)	−0.05 (−0.21, 0.08)	0.02 (−0.11, 0.14)	−0.2 (−0.36, −0.06)	−0.43 (−0.6, −0.27)	−0.97 (−1.61, −0.48)
Tricuspid valve atresia and stenosis	−0.49 (−0.7, −0.31)	−0.1 (−0.24, 0.03)	−0.63 (−0.8, −0.48)	0.15 (0.07, 0.22)	−0.88 (−1.06, −0.72)	−1.21 (−1.46, −0.99)
Aniridia	0.2 (−0.53, 0.58)	−1.92 (−5.62, −0.28)	0.46 (0.23, 0.62)	0.42 (0.12, 0.61)	0.31 (−0.07, 0.55)	–

Table 8 performs a similar function for Population Attributable Risk (PAR). Cloacal extrophy again heads the list from a PAR of 56.75% after cannabis exposure.

**Table 8:** Population Attributable Risk by Substance

Congenital Anomaly	Cigarettes PAR	Binge Alcohol PAR	Analgesics PAR	Cocaine PAR	Cannabis PAR	Cannabidiol PAR
Obstructive genitourinary defect	−0.03 (−0.05, −0.01)	0.01 (−0.02, 0.03)	−0.03 (−0.05, −0.02)	0.07 (0.05, 0.09)	−0.04 (−0.06, −0.03)	0.47 (0.38, 0.55)
Cleft lip with and without cleft palate	0.02 (−0.01, 0.04)	−0.02 (−0.05, 0.01)	0.09 (0.06, 0.12)	0.01 (−0.03, 0.05)	−0.02 (−0.04, 0)	0.34 (0.07, 0.53)
Hirschsprung disease (congenital megacolon)	0.05 (−0.02, 0.11)	−0.24 (−0.32, −0.16)	0.02 (−0.04, 0.08)	0 (−0.09, 0.09)	0.12 (0.06, 0.17)	0.22 (−0.25, 0.51)
Congenital hip dislocation	−0.03 (−0.08, 0.02)	0.05 (−0.02, 0.11)	−0.02 (−0.05, 0.02)	0.28 (0.23, 0.33)	0.25 (0.22, 0.28)	0.21 (−0.14, 0.46)
Pulmonary valve atresia	0.01 (−0.02, 0.05)	−0.27 (−0.32, −0.22)	0.18 (0.14, 0.23)	−0.02 (−0.09, 0.05)	0.11 (0.06, 0.16)	0.12 (0.06, 0.17)
Biliary atresia	0.01 (−0.04, 0.05)	−0.16 (−0.22, −0.1)	−0.04 (−0.1, 0.01)	0.06 (−0.04, 0.14)	0.07 (0.01, 0.13)	0.1 (0.01, 0.18)
Patent ductus arteriosus	−0.02 (−0.04, 0)	−0.16 (−0.18, −0.13)	0.04 (0.02, 0.05)	−0.12 (−0.14, −0.09)	−0.16 (−0.17, −0.15)	0.07 (−0.12, 0.23)
Trisomy 13	−0.11 (−0.13, −0.08)	−0.01 (−0.06, 0.03)	−0.06 (−0.08, −0.04)	0.3 (0.27, 0.32)	0.11 (0.09, 0.12)	0.07 (0, 0.13)
Small intestinal atresia/stenosis	−0.02 (−0.04, −0.01)	−0.05 (−0.08, −0.01)	0.03 (0, 0.06)	0.06 (0, 0.11)	0.1 (0.06, 0.14)	0.06 (0.03, 0.09)
Cloacal exstrophy	0.27 (0.22, 0.31)	−0.05 (−0.09, −0.01)	0.1 (0.07, 0.14)	−0.23 (−0.29, −0.16)	0.57 (0.51, 0.62)	0.06 (0.02, 0.1)
Single ventricle	−0.03 (−0.06, 0.01)	−0.25 (−0.31, −0.18)	0.02 (−0.03, 0.07)	−0.13 (−0.22, −0.04)	0.09 (0.02, 0.16)	0.06 (0, 0.11)
Total anomalous pulmonary venous connection	−0.08 (−0.1, −0.05)	−0.17 (−0.23, −0.11)	0.13 (0.08, 0.19)	0.15 (0.07, 0.23)	0.02 (−0.04, 0.08)	0.05 (−0.01, 0.11)
Diaphragmatic hernia	0.05 (0.03, 0.08)	−0.06 (−0.1, −0.03)	0.05 (0.02, 0.08)	−0.08 (−0.13, −0.03)	0.09 (0.06, 0.12)	0.04 (0, 0.08)
Double outlet right ventricle	0.01 (−0.02, 0.04)	−0.15 (−0.2, −0.1)	0.04 (0, 0.07)	−0.05 (−0.11, 0)	0.09 (0.04, 0.14)	0.04 (0, 0.07)
Deletion 22q11.2	−0.11 (−0.16, −0.06)	0.09 (−0.01, 0.19)	0.4 (0.33, 0.47)	0.31 (0.2, 0.4)	0.09 (0.02, 0.16)	0.03 (−0.02, 0.09)
Trisomy 21 (Down syndrome)	−0.04 (−0.05, −0.03)	0.01 (0, 0.03)	0.01 (0, 0.01)	0.07 (0.06, 0.08)	0.06 (0.05, 0.07)	0.03 (0.01, 0.05)
Atrioventricular septal defect	−0.01 (−0.03, 0)	−0.08 (−0.11, −0.06)	0.02 (0, 0.04)	−0.04 (−0.07, −0.01)	0.02 (0, 0.04)	0.03 (0, 0.06)
Trisomy 18	−0.09 (−0.11, −0.07)	−0.04 (−0.07, −0.01)	0.02 (0.01, 0.04)	0.16 (0.14, 0.18)	0.11 (0.1, 0.13)	0.02 (−0.02, 0.07)
Clubfoot	0.01 (−0.01, 0.02)	−0.05 (−0.06, −0.03)	0.01 (−0.01, 0.02)	0 (−0.03, 0.02)	0.02 (0, 0.04)	0.02 (0, 0.03)
Hypoplastic left heart syndrome	0.05 (0.02, 0.07)	−0.13 (−0.16, −0.1)	0.04 (0.02, 0.07)	−0.05 (−0.09, −0.01)	0.04 (0.01, 0.07)	0.01 (−0.03, 0.06)
Aortic valve stenosis	−0.01 (−0.04, 0.01)	−0.12 (−0.15, −0.08)	0.18 (0.15, 0.2)	0.02 (−0.02, 0.06)	−0.04 (−0.07, −0.02)	0.01 (−0.04, 0.06)
Cleft lip alone	0.01 (−0.01, 0.03)	0.01 (−0.02, 0.05)	0.06 (0.03, 0.1)	−0.12 (−0.17, −0.07)	−0.02 (−0.05, 0.02)	0.01 (−0.02, 0.03)
Transposition of great arteries	0.08 (0.05, 0.11)	−0.08 (−0.11, −0.05)	0.07 (0.05, 0.1)	−0.08 (−0.12, −0.05)	0 (−0.02, 0.03)	0.01 (−0.03, 0.04)
Anotia/microtia	−0.19 (−0.2, −0.17)	−0.05 (−0.09, −0.01)	0.12 (0.1, 0.14)	0.25 (0.22, 0.28)	0 (−0.02, 0.02)	0 (−0.05, 0.05)
Rectal and large intestinal atresia/stenosis	0.05 (0.03, 0.07)	−0.06 (−0.09, −0.04)	0.02 (0, 0.05)	−0.07 (−0.11, −0.04)	−0.04 (−0.07, −0.02)	0 (−0.03, 0.03)
Spina bifida without anencephalus	0.01 (−0.01, 0.03)	−0.06 (−0.09, −0.04)	0.12 (0.1, 0.14)	0 (−0.02, 0.02)	0.02 (0, 0.04)	0 (−0.04, 0.04)
Gastroschisis	0 (−0.02, 0.02)	−0.04 (−0.06, −0.02)	0.15 (0.12, 0.17)	−0.02 (−0.05, 0.02)	−0.03 (−0.05, −0.01)	0 (−0.04, 0.03)
Cleft lip with cleft palate	0.03 (0.01, 0.04)	−0.06 (−0.09, −0.03)	0.12 (0.1, 0.15)	−0.04 (−0.08, 0)	−0.02 (−0.05, 0.01)	0 (−0.03, 0.02)
Congenital posterior urethral valves	0.03 (−0.02, 0.07)	−0.17 (−0.21, −0.12)	0.02 (−0.03, 0.06)	−0.09 (−0.17, −0.02)	0.09 (0.04, 0.13)	−0.01 (−0.04, 0.02)
Coarctation of the aorta	0.04 (0.02, 0.05)	−0.12 (−0.14, −0.1)	0.05 (0.03, 0.07)	−0.08 (−0.11, −0.05)	0.14 (0.12, 0.15)	−0.01 (−0.04, 0.02)
Anencephalus	−0.03 (−0.05, 0)	−0.12 (−0.16, −0.09)	0.12 (0.1, 0.14)	0.02 (−0.01, 0.04)	−0.03 (−0.05, −0.02)	−0.02 (−0.07, 0.03)
Esophageal atresia/tracheoesophageal fistula	0.02 (0, 0.04)	0.02 (−0.01, 0.06)	0.01 (−0.02, 0.05)	0 (−0.05, 0.05)	0.04 (0.01, 0.08)	−0.02 (−0.06, 0.03)
Interrupted aortic arch	0.04 (−0.01, 0.09)	−0.05 (−0.13, 0.03)	0.12 (0.04, 0.19)	−0.13 (−0.28, −0.01)	0.02 (−0.09, 0.11)	−0.02 (−0.09, 0.04)
Tetralogy of Fallot	0.02 (0.01, 0.04)	−0.09 (−0.11, −0.06)	0.01 (−0.01, 0.04)	−0.01 (−0.04, 0.03)	−0.02 (−0.04, 0.01)	−0.02 (−0.06, 0.01)
Encephalocele	0.05 (0.01, 0.09)	−0.13 (−0.18, −0.08)	0.03 (−0.01, 0.06)	−0.05 (−0.1, 0)	−0.03 (−0.06, 0.01)	−0.02 (−0.1, 0.05)
Dextro-transposition of great arteries (d-TGA)	0.04 (0.02, 0.07)	−0.04 (−0.08, 0)	−0.01 (−0.05, 0.02)	−0.07 (−0.14, −0.01)	−0.13 (−0.17, −0.09)	−0.03 (−0.08, 0.01)
Limb deficiencies (reduction defects)	0.02 (0, 0.04)	−0.05 (−0.08, −0.02)	0.03 (0, 0.05)	−0.1 (−0.14, −0.06)	−0.13 (−0.15, −0.1)	−0.04 (−0.06, −0.02)
Turner syndrome	−0.12 (−0.15, −0.09)	0.05 (0, 0.09)	0 (−0.02, 0.02)	0.06 (0.02, 0.1)	0.12 (0.1, 0.14)	−0.05 (−0.08, −0.03)
Congenital cataract	−0.02 (−0.04, 0.01)	−0.03 (−0.08, 0.01)	0.03 (−0.01, 0.07)	−0.02 (−0.08, 0.04)	−0.02 (−0.06, 0.02)	−0.06 (−0.12, 0)
Cleft palate alone	0.05 (0.03, 0.06)	0.01 (−0.02, 0.03)	0.05 (0.03, 0.08)	−0.02 (−0.05, 0.01)	−0.02 (−0.04, 0)	−0.07 (−0.1, −0.04)
Bladder exstrophy	0.13 (0.04, 0.21)	−0.03 (−0.13, 0.07)	0.05 (−0.05, 0.14)	−0.07 (−0.23, 0.06)	0.01 (−0.1, 0.11)	−0.07 (−0.24, 0.08)
Craniosynostosis	0.03 (0.01, 0.04)	−0.15 (−0.18, −0.12)	0.04 (0, 0.07)	−0.01 (−0.08, 0.05)	−0.25 (−0.28, −0.22)	−0.07 (−0.1, −0.05)
Pulmonary valve atresia and stenosis	0 (−0.01, 0.01)	−0.11 (−0.13, −0.1)	0.01 (−0.01, 0.02)	−0.06 (−0.09, −0.04)	−0.04 (−0.06, −0.03)	−0.08 (−0.11, −0.06)
Holoprosencephaly	0.15 (0.12, 0.18)	−0.18 (−0.22, −0.14)	−0.06 (−0.08, −0.04)	−0.61 (−0.65, −0.57)	0.1 (0.07, 0.13)	−0.1 (−0.13, −0.07)
Ebstein anomaly	0.03 (−0.01, 0.07)	−0.12 (−0.17, −0.06)	0.11 (0.05, 0.16)	−0.05 (−0.12, 0.03)	−0.05 (−0.1, 0)	−0.1 (−0.19, −0.02)
Choanal atresia	0.09 (0.05, 0.13)	0.01 (−0.04, 0.06)	−0.07 (−0.11, −0.03)	−0.07 (−0.14, −0.01)	−0.1 (−0.15, −0.06)	−0.11 (−0.18, −0.05)
Omphalocele	0.04 (0.02, 0.07)	−0.08 (−0.12, −0.05)	0.03 (0, 0.05)	−0.09 (−0.13, −0.06)	−0.04 (−0.06, −0.01)	−0.12 (−0.17, −0.06)
Ventricular septal defect	0.06 (0.05, 0.06)	−0.06 (−0.07, −0.06)	−0.05 (−0.06, −0.04)	−0.11 (−0.12, −0.1)	−0.08 (−0.09, −0.07)	−0.13 (−0.14, −0.12)
Pyloric stenosis	0.24 (0.21, 0.26)	−0.39 (−0.41, −0.36)	0.2 (0.19, 0.22)	−0.16 (−0.19, −0.14)	−0.03 (−0.04, −0.01)	−0.14 (−0.31, 0.01)
Common truncus (truncus arteriosus)	0.22 (0.17, 0.27)	−0.18 (−0.24, −0.13)	0.01 (−0.05, 0.06)	−0.18 (−0.28, −0.1)	−0.13 (−0.18, −0.07)	−0.14 (−0.23, −0.06)
Hypospadias	0.12 (0.11, 0.12)	−0.05 (−0.05, −0.04)	−0.01 (−0.01, 0)	0 (−0.01, 0.01)	−0.13 (−0.13, −0.12)	−0.18 (−0.19, −0.17)
Anophthalmia/microphthalmia	−0.05 (−0.09, −0.02)	0.01 (−0.04, 0.06)	0.06 (0.03, 0.09)	0.08 (0.04, 0.13)	−0.21 (−0.23, −0.18)	−0.18 (−0.24, −0.12)
Hydrocephalus without spina bifida	0.09 (0.06, 0.12)	0.01 (−0.02, 0.05)	−0.04 (−0.06, −0.02)	−0.01 (−0.05, 0.02)	0.01 (−0.01, 0.04)	−0.19 (−0.5, 0.06)
Amniotic Bands	−0.01 (−0.05, 0.04)	−0.14 (−0.22, −0.06)	0.1 (0, 0.2)	−0.11 (−0.22, −0.01)	−0.03 (−0.1, 0.03)	−0.19 (−1.07, 0.31)
Renal agenesis/hypoplasia	0.06 (0.04, 0.08)	−0.03 (−0.05, 0)	−0.02 (−0.03, 0)	−0.09 (−0.12, −0.06)	−0.23 (−0.25, −0.22)	−0.26 (−0.29, −0.23)
Reduction deformity, Lower limbs	0.06 (0, 0.11)	−0.02 (−0.09, 0.04)	0.01 (−0.05, 0.07)	−0.1 (−0.19, −0.01)	−0.05 (−0.1, −0.01)	−0.31 (−1.07, 0.17)
Atrial septal defect	0.28 (0.27, 0.28)	−0.2 (−0.21, −0.2)	0.09 (0.09, 0.1)	−0.17 (−0.18, −0.16)	−0.13 (−0.14, −0.13)	−0.34 (−0.35, −0.33)
Tricuspid valve atresia and stenosis	−0.09 (−0.12, −0.07)	−0.03 (−0.08, 0.01)	−0.16 (−0.18, −0.13)	0.09 (0.04, 0.13)	−0.23 (−0.26, −0.21)	−0.42 (−0.47, −0.37)
Microcephalus	0.11 (0.07, 0.16)	−0.11 (−0.16, −0.06)	0.03 (−0.01, 0.06)	−0.04 (−0.09, 0.01)	−0.09 (−0.12, −0.07)	−0.44 (−0.78, −0.17)
Epispadias	−0.09 (−0.2, 0.01)	0.09 (−0.04, 0.2)	−0.1 (−0.17, −0.03)	0.13 (0.01, 0.24)	0.07 (0, 0.14)	−0.48 (−0.96, −0.11)
Reduction deformity, Upper limbs	−0.03 (−0.07, 0.01)	−0.02 (−0.07, 0.03)	0.01 (−0.04, 0.05)	−0.09 (−0.15, −0.03)	−0.1 (−0.13, −0.07)	−0.92 (−1.52, −0.46)
Aniridia	0.09 (−0.22, 0.32)	−0.35 (−0.57, −0.16)	0.21 (0.07, 0.33)	0.25 (0.04, 0.42)	0.11 (−0.04, 0.24)	–

Applicable *P*-values are listed together by substance in Table 9. In reading this table it should be noted that P values in R are only computed down to 2.2x10^−320^. Such values in the table may be better understood as zeroes.

**Table 9:** Significance Levels by Substance

Congenital Anomaly	Cigarettes ***P***-Value	Binge Alcohol ***P***-Value	Analgesics ***P***-Value	Cocaine ***P***-Value	Cannabis ***P***-Value	Cannabidiol ***P***-Value
Atrial septal defect	2.2e-320	0.0215	9.26E-39	0.00536446	1.09E-08	2.2e-320
Ventricular septal defect	7.36E-08	0.1490	1.24E-20	1.93E-04	0.0033	2.2e-320
Hypospadias	2.2e-320	0.4362	0.0317	0.7536	1.38E-05	1.02E-289
Tricuspid valve atresia and stenosis	7.29E-10	0.1377	4.01E-159	6.31E-04	3.93E-04	1.36E-50
Renal agenesis/hypoplasia	5.30E-11	0.0215	0.0606	2.82E-09	0.0028	1.63E-50
Obstructive genitourinary defect	0.0012	0.4876	4.12E-05	2.37E-09	3.92E-12	2.22E-15
Pulmonary valve atresia and stenosis	0.9716	0.0408	0.4950	3.64E-08	1.27E-08	9.14E-12
Anophthalmia/microphthalmia	8.86E-04	0.6781	7.92E-05	3.62E-04	1.48E-12	2.12E-08
Craniosynostosis	1.80E-05	1.0000	0.0266	0.7020	7.18E-05	2.31E-08
Holoprosencephaly	1.66E-28	2.00E-15	5.89E-08	2.02E-04	2.90E-12	2.34E-08
Reduction deformity, Upper limbs	0.1301	0.4407	0.7110	0.0042	1.15E-09	2.01E-06
Cleft palate alone	2.29E-11	0.6532	3.78E-07	0.2282	0.0892	3.47E-06
Small intestinal atresia/stenosis	0.0106	0.0125	0.0775	0.0531	4.47E-06	5.93E-06
Pulmonary valve atresia	0.4522	0.0978	2.23E-17	0.6602	2.62E-05	1.02E-05
Omphalocele	8.62E-04	6.56E-06	0.0288	7.27E-07	0.0056	2.49E-05
Cloacal exstrophy	1.36E-45	0.0284	1.91E-12	6.90E-11	2.13E-86	5.45E-04
Turner syndrome	9.14E-11	0.0367	0.7522	0.0014	7.69E-49	5.63E-04
Common truncus (truncus arteriosus)	7.03E-22	1.02E-08	0.8321	7.90E-05	2.51E-05	6.55E-04
Microcephalus	3.39E-07	7.86E-06	0.1067	0.1136	1.90E-13	7.11E-04
Choanal atresia	1.42E-07	0.7186	0.0014	0.0290	2.26E-05	7.34E-04
Limb deficiencies (reduction defects)	0.0428	0.0034	0.0405	1.72E-06	2.33E-06	8.14E-04
Trisomy 21 (Down syndrome)	1.75E-07	0.0895	0.1491	1.49E-55	4.02E-26	0.0021
Clubfoot	0.4663	3.66E-06	0.2788	0.7378	0.0136	0.0048
Epispadias	0.0915	0.1591	0.0084	0.0242	0.0287	0.0081
Cleft lip with and without cleft palate	0.1248	0.1441	3.43E-11	0.5715	0.0887	0.0159
Ebstein anomaly	0.1232	1.57E-04	3.81E-05	0.2279	0.0643	0.0177
Biliary atresia	0.7657	4.48E-06	0.1548	0.2026	0.0244	0.0233
Diaphragmatic hernia	5.26E-07	5.72E-05	0.0011	0.0013	2.11E-08	0.0373
Double outlet right ventricle	0.5739	4.13E-08	0.0314	0.0743	7.31E-04	0.0379
Congenital cataract	0.2114	0.1134	0.1381	0.5892	0.2537	0.0416
Trisomy 13	1.77E-11	0.5945	4.20E-09	3.06E-106	3.50E-06	0.0427
Single ventricle	0.1289	2.42E-10	0.3626	0.0037	0.0060	0.0482
Pyloric stenosis	1.38E-84	0.1051	1.78E-122	9.40E-29	4.82E-04	0.0657
Total anomalous pulmonary venous connection	1.05E-07	1.64E-06	9.84E-07	3.78E-04	0.4381	0.0773
Atrioventricular septal defect	0.0937	6.48E-12	0.0269	0.0073	0.0470	0.0854
Hydrocephalus without spina bifida	1.84E-09	0.4705	0.0011	0.5085	0.2200	0.1441
Dextro-transposition of great arteries (d-TGA)	8.25E-04	0.0513	0.4822	0.0198	1.40E-10	0.1529
Deletion 22q11.2	6.98E-05	0.0672	3.67E-28	4.72E-08	0.0051	0.2047
Congenital hip dislocation	0.1991	0.1493	0.3536	1.13E-27	7.27E-70	0.2063
Reduction deformity, Lower limbs	0.0231	0.5017	0.6312	0.0277	0.0253	0.2401
Tetralogy of Fallot	0.0047	7.04E-11	0.2681	0.6597	0.1692	0.2530
Hirschsprung disease (congenital megacolon)	0.1818	1.55E-08	0.4987	0.9565	6.69E-06	0.3010
Trisomy 18	6.05E-04	0.0140	0.0034	2.43E-08	1.06E-61	0.3486
Bladder exstrophy	0.0011	0.5975	0.3200	0.3343	0.8681	0.3893
Patent ductus arteriosus	0.0375	0.8028	2.36E-07	1.22E-09	5.95E-39	0.4386
Esophageal atresia/tracheoesophageal fistula	0.0977	0.2148	0.3880	0.8914	0.0195	0.4602
Hypoplastic left heart syndrome	1.91E-05	1.35E-14	0.0014	0.0080	0.0048	0.5102
Anencephalus	0.0269	1.45E-10	4.05E-44	0.1442	7.15E-05	0.5233
Coarctation of the aorta	5.48E-07	0.8820	3.82E-08	1.56E-07	9.74E-45	0.5283
Amniotic Bands	0.7468	0.0026	0.0271	0.0681	0.3785	0.5349
Encephalocele	0.0079	4.74E-06	0.1125	0.0397	0.1289	0.5605
Congenital posterior urethral valves	0.2039	8.57E-11	0.4012	0.0184	1.35E-04	0.5725
Interrupted aortic arch	0.0582	0.2411	0.0022	0.0472	0.7274	0.5788
Cleft lip alone	0.2215	0.4773	1.95E-04	1.40E-06	0.4216	0.5998
Aortic valve stenosis	0.4160	6.46E-09	9.78E-36	0.4210	0.0011	0.6318
Transposition of great arteries	1.49E-08	5.27E-07	4.54E-08	1.54E-05	0.7669	0.7385
Cleft lip with cleft palate	1.92E-04	2.58E-04	3.32E-04	0.0304	0.1508	0.7455
Anotia/microtia	9.45E-10	0.0148	4.05E-37	2.02E-64	0.9670	0.8853
Gastroschisis	0.9978	0.0014	9.74E-13	0.3662	0.0098	0.8919
Rectal and large intestinal atresia/stenosis	4.34E-08	2.94E-06	0.0493	1.69E-04	0.0011	0.8966
Spina bifida without anencephalus	0.2806	5.86E-06	2.14E-29	0.9854	0.0181	0.9332
Aniridia	0.5068	0.0073	6.17E-04	0.0096	0.0952	–

Minimum E-Values for these comparisons are shown in Table 10 by substance.

**Table 10:** E-Values by Substance

Congenital Anomaly	Cigarettes E-Value	Binge Alcohol E-Value	Analgesics E-Value	Cocaine E-Value	Cannabis E-Value	Cannabidiol E-Value
Obstructive genitourinary defect		1.00		1.46		2.64
Pulmonary valve atresia	1.00		2.28		1.53	1.64
Small intestinal atresia/stenosis			1.00	1.00	1.49	1.54
Cloacal exstrophy	4.32		2.16		7.61	1.48
Cleft lip with and without cleft palate	1.00		1.70	1.00		1.37
Clubfoot	1.00		1.00		1.14	1.20
Biliary atresia	1.00			1.00	1.17	1.18
Trisomy 21 (Down syndrome)		1.00	1.00	1.46	1.49	1.16
Double outlet right ventricle	1.00		1.11		1.36	1.10
Diaphragmatic hernia	1.52		1.31		1.57	1.07
Trisomy 13				2.73	1.75	1.07
Single ventricle			1.00		1.30	1.04
Transposition of great arteries	1.59		1.54		1.00	1.00
Rectal and large intestinal atresia/stenosis	1.50		1.01			1.00
Hypoplastic left heart syndrome	1.44		1.26		1.20	1.00
Cleft lip alone	1.00	1.00	1.38			1.00
Hirschsprung disease (congenital megacolon)	1.00		1.00	1.00	1.77	1.00
Spina bifida without anencephalus	1.00		1.93	1.00	1.10	1.00
Anotia/microtia			1.93	2.43	1.00	1.00
Aortic valve stenosis			2.32	1.00		1.00
Atrioventricular septal defect			1.10		1.03	1.00
Congenital hip dislocation		1.00		2.69	3.57	1.00
Deletion 22q11.2		1.00	4.22	2.28	1.42	1.00
Patent ductus arteriosus			1.37			1.00
Total anomalous pulmonary venous connection			1.79	1.51	1.00	1.00
Trisomy 18			1.16	1.91	1.85	1.00
Atrial septal defect	4.38		1.89			
Common truncus (truncus arteriosus)	2.99		1.00			
Holoprosencephaly	2.80				1.60	
Pyloric stenosis	2.64		3.00			
Hypospadias	2.48					
Choanal atresia	1.77	1.00				
Hydrocephalus without spina bifida	1.73	1.00			1.00	
Bladder exstrophy	1.67		1.00		1.00	
Microcephalus	1.65		1.00			
Renal agenesis/hypoplasia	1.62					
Ventricular septal defect	1.61					
Cleft palate alone	1.58	1.00	1.41			
Craniosynostosis	1.53		1.13			
Coarctation of the aorta	1.42		1.43		1.97	
Dextro-transposition of great arteries (d-TGA)	1.36					
Omphalocele	1.35		1.09			
Cleft lip with cleft palate	1.33		1.88			
Encephalocele	1.28		1.00			
Tetralogy of Fallot	1.20		1.00			
Reduction deformity, Lower limbs	1.20		1.00			
Limb deficiencies (reduction defects)	1.06		1.06			
Aniridia	1.00		1.90	1.53	1.00	
Congenital posterior urethral valves	1.00		1.00		1.56	
Ebstein anomaly	1.00		1.60			
Esophageal atresia/tracheoesophageal fistula	1.00	1.00	1.00	1.00	1.15	
Interrupted aortic arch	1.00		1.49		1.00	
Amniotic Bands			1.26			
Anencephalus			1.91	1.00		
Anophthalmia/microphthalmia		1.00	1.39	1.35		
Congenital cataract			1.00			
Epispadias		1.00		1.25	1.20	
Gastroschisis			2.12			
Pulmonary valve atresia and stenosis			1.00			
Reduction deformity, Upper limbs			1.00			
Tricuspid valve atresia and stenosis				1.35		
Turner syndrome		1.10	1.00	1.25	2.31	

### Summary of bivariate analyses

Given that the above tables present a lot of information it is of interest to distil this information down into more intellectually digestible components.

Supplementary Table 11 extracts the 85 ETOPFACARs which have significant E-Values for the 35 cannabis related CAs, the 40 THC related CAs and the 11 cannabidiol CAs considered as continuous variables. The table is arranged in descending order of the lower bound of the E-Values. 37/85 E-Values are greater than 9.0 which is the E-Value for the tobacco-lung cancer relationship and 84/85 are greater than 1.25 which is the quoted cut-off for causality [68].

Table 11 re-lists the 41 CAs listed in Table 20 and retains only the ETOPFACAR with the highest minimum E-Value. In this Table 28/41 are greater than 9.0 and 40/41 are greater than 1.25. On this list 28 CAs are related to cannabis, 5 to THC and 8 to cannabidiol.

**Table 11:** Summary Single CAs with Significant Cannabinoid E-Values Continuous Variables

Defect	No.	System	Term	Estimate	Std.Error	Students T	P_Value	S.D.	E-Value-Point Estimate	E-Value-Lower Limit
Congenital hip dislocation	1	Limb	CBD	298.2937	55.1100	5.4127	0.0000	3.8459	9.00E+30	7.53E+19
Small intestinal atresia/stenosis	2	GIT	CBD	61.6605	12.7480	4.8369	0.0000	1.1814	8.48E+20	3.86E+12
Trisomy 21 (Down syndrome)	3	Chromosomes	Cannabis	221.1194	25.4625	8.6841	0.0000	10.2305	6.97E+08	8.30E+06
Biliary atresia	4	GIT	CBD	10.9598	2.9445	3.7222	0.0002	0.3922	2.22E+11	3.48E+05
Interrupted aortic arch	5	CVS	Cannabis	15.4036	3.1814	4.8418	0.0000	0.8305	4.28E+07	4.68E+04
Obstructive genitourinary defect	6	GUT	CBD	486.0939	176.6878	2.7511	0.0072	13.0815	9.69E+14	3.51E+04
Hirschsprung disease (congenital megacolon)	7	GIT	CBD	38.1800	14.1676	2.6949	0.0084	1.0029	2.22E+15	2.67E+04
Clubfoot	8	Limb	Cannabis	94.0309	21.7820	4.3169	0.0000	5.4311	1.39E+07	1.10E+04
Trisomy 13	9	Chromosomes	Cannabis	75.1394	14.1320	5.3170	0.0000	5.1679	1.11E+06	8.58E+03
Congenital posterior urethral valves	10	GUT	Cannabis	23.9399	6.0470	3.9590	0.0001	1.6001	1.64E+06	1.96E+03
Trisomy 18	11	Chromosomes	Cannabis	126.9696	26.3799	4.8131	0.0000	10.0424	1.99E+05	1.85E+03
Esophageal atresia/tracheoesophageal fistula	12	GIT	Cannabis	8.8449	1.8993	4.6570	0.0000	0.7176	1.49E+05	1.34E+03
Hypospadias	13	GUT	Cannabis	277.1790	62.0518	4.4669	0.0000	23.4595	9.34E+04	842.36
Rectal and large intestinal atresia/stenosis	14	GIT	CBD	26.0458	8.9678	2.9044	0.0040	1.3051	1.54E+08	751.61
Diaphragmatic hernia	15	Body Wall	CBD	21.8501	7.9675	2.7424	0.0065	1.1678	4.96E+07	263.36
Deletion 22q11.2	16	Chromosomes	Cannabis	6.6430	2.1356	3.1106	0.0024	0.5153	2.49E+05	155.04
Turner syndrom**e**	17	Chromosomes	Cannabis	85.6995	27.3283	3.1359	0.0021	6.9321	1.54E+05	137.32
Epispadias	18	GUT	Cannabis	12.5446	4.8274	2.5986	0.0111	0.7392	1.02E+07	90.57
Renal agenesis/hypoplasia	19	GUT	Cannabis	27.3954	8.0283	3.4124	0.0007	3.0315	7.45E+03	66.37
Anotia/microtia	20	Face	Cannabis	37.2830	10.9541	3.4036	0.0008	4.1220	7.51E+03	65.76
Cleft palate alone	21	Face	Cannabis	24.1946	7.4701	3.2389	0.0014	2.7271	6.42E+03	48.45
Encephalocele	22	CNS	Cannabis	11.3770	3.4999	3.2507	0.0013	1.3138	5.29E+03	45.63
Aortic valve stenosis	23	CVS	Cannabis	17.8815	5.6987	3.1378	0.0019	2.1020	4.60E+03	36.41
Ventricular septal defect	24	CVS	Cannabis	166.2143	53.4999	3.1068	0.0021	19.9528	3.92E+03	32.64
Pulmonary valve atresia	25	CVS	Cannabis	9.4232	3.2900	2.8642	0.0047	1.0048	1.02E+04	29.43
Omphalocele	26	Body Wall	Cannabis	28.8975	9.4470	3.0589	0.0025	3.5144	3.55E+03	29.18
Hypoplastic left heart syndrome	27	CVS	Cannabis	10.7890	3.7873	2.8487	0.0047	1.4621	1.65E+03	15.88
Limb deficiencies (reduction defects)	28	Limb	Cannabis	21.4215	8.5782	2.4972	0.0134	2.6156	3.45E+03	9.53
Bladder exstrophy	29	GUT	Cannabis	1.0618	0.4420	2.4021	0.0170	0.1593	8.61E+02	5.62
Tetralogy of Fallot	30	CVS	Cannabis	9.9067	4.1188	2.4052	0.0168	1.6031	5.53E+02	5.16
Total anomalous pulmonary venous connection	31	CVS	Cannabis	3.9176	1.7901	2.1885	0.0299	0.4968	2.61E+03	3.71
Reduction deformity, Lower limbs	32	Limb	Cannabis	16.8233	8.1886	2.0545	0.0420	1.5723	3.39E+04	2.57
Coarctation of the aorta	33	CVS	Cannabis	22.5596	10.7794	2.0928	0.0372	4.0947	300.37	2.12
Atrial septal defect	34	CVS	Cannabis	285.3616	136.7781	2.0863	0.0378	51.3723	313.06	2.08
Spina bifida without anencephalus	35	CNS	THC	2.8769	0.8458	3.4015	0.0008	4.0422	3.23	1.96
Choanal atresia	36	Face	THC	0.4877	0.1646	2.9621	0.0033	0.7074	3.15	1.78
Anophthalmia/microphthalmia	37	CNS	THC	1.1940	0.4167	2.8651	0.0045	1.7156	3.17	1.74
Transposition of great arteries	38	CVS	CBD	19.6282	9.8766	1.9873	0.0479	1.4902	3.21E+05	1.71
Holoprosencephaly	39	Face	THC	8.0303	3.0912	2.5978	0.0104	10.1025	3.54	1.68
Congenital cataract	40	Face	Cannabis	5.9492	2.9939	1.9871	0.0479	1.0436	357.58	1.39
Single ventricle	41	CVS	THC	0.6263	0.3014	2.0780	0.0394	0.9759	2.99	1.22

To further condense this material Table 12 lists the organ systems of the various CAs listed in descending order of the percentages of the listed CAs for that organ system. It is noted immediately that the list is headed by chromosomal disorders, but that genitourinary, gastrointestinal, limb defects, body wall defects, cardiovascular anomalies and facial anomalies all have more than 50% of their listed CAs positively and potentially causally associated with one of the various cannabinoids.

**Table 12:** Summary Continuous Variables by System

System	No. Anomalies	Total No. Anomalies	% of Total Anomalies
Chromosomes	5	5	100.0%
GUT	6	7	85.7%
GIT	5	6	83.3%
Limb	4	5	80.0%
Body Wall	2	3	66.7%
CVS	11	19	57.9%
Face	5	9	55.6%
CNS	3	7	42.9%
Total	**41**	**61**	**67.2%**

A similar exercise can be performed on the CARs (not corrected for ETOPFAs) treated as categorical variables comparing the highest Quintile (Quintile 5) with the lowest quintile (Quintile 1, or the absence of data, Quintile 2).

Supplementary Table 12 shows selected parameters from this comparison extracted for those 31 CARs with elevated minimum E-Values listed in descending order of E-Values. 21 of these CARs are related to cannabis and 12 are related to cannabidiol.

Table 13 removes the duplicates from these CARs and retains the most significant results leaving 23 CARs, 17 related to cannabis and 6 to cannabidiol.

**Table 13:** Summary CAs with Significant Cannabinoid E-Values Categorical Variables

Defect	No.	System	Term	PR_C.I.	AFE_C.I.	ChiSqu	*P*-Value	E-Value-Point Estimate	E-Value-Lower Limit
Cloacal exstrophy	1	GIT	Cannabis	4.85 (4.08, 5.77)	0.79 (0.75, 0.83)	386.7336	2.13E-86	9.17	7.61
Congenital hip dislocation	2	Limb	Cannabis	2.28 (2.08, 2.51)	0.56 (0.52, 0.60)	310.8170	7.27E-70	3.99	3.57
Coarctation of the aorta	3	CVS	Cannabis	1.38 (1.31, 1.45)	0.28 (0.24, 0.31)	152.3739	2.64E-35	2.10	1.95
Obstructive genitourinary defect	4	GUT	CBD	1.92 (1.63, 2.27)	0.48 (0.39, 0.56)	62.8480	2.22E-15	3.25	2.64
Turner syndrome	5	Chromosomes	Cannabis	1.54 (1.36, 1.75)	0.35 (0.26, 0.43)	46.5388	4.58E-12	2.45	2.06
Trisomy 21 (Down syndrome)	6	Chromosomes	Cannabis	1.12 (1.08, 1.16)	0.11 (0.08, 0.14)	45.1282	9.42E-12	1.49	1.39
Diaphragmatic hernia	7	Body Wall	Cannabis	1.24 (1.15, 1.34)	0.20 (0.13, 0.26)	31.3922	1.09E-08	1.80	1.57
Trisomy 18	8	Chromosomes	Cannabis	1.22 (1.13, 1.32)	0.18 (0.11, 0.24)	25.4031	2.41E-07	1.73	1.51
Small intestinal atresia/stenosis	9	GIT	Cannabis	1.22 (1.12, 1.33)	0.18 (0.11, 0.25)	21.0508	2.33E-06	1.75	1.49
Small intestinal atresia/stenosis	9	GIT	CBD	1.26 (1.14, 1.39)	0.21 (0.12, 0.28)	20.5107	5.93E-06	1.83	1.54
Hirschsprung disease (congenital megacolon)	10	GIT	Cannabis	1.46 (1.24, 1.72)	0.31 (0.19, 0.42)	20.2790	3.50E-06	2.27	1.77
Pulmonary valve atresia	11	CVS	CBD	1.35 (1.18, 1.55)	0.26 (0.15, 0.35)	19.4818	1.02E-05	2.04	1.64
Holoprosencephaly	12	Face	Cannabis	1.27 (1.12, 1.43)	0.21 (0.11, 0.30)	14.9227	5.94E-05	1.86	1.50
Pulmonary valve atresia	13	CVS	Cannabis	1.28 (1.13, 1.45)	0.22 (0.11, 0.31)	14.7343	6.56E-05	1.87	1.50
Congenital posterior urethral valves	14	GUT	Cannabis	1.33 (1.15, 1.54)	0.25 (0.13, 0.35)	14.5658	7.18E-05	1.99	1.56
Cloacal exstrophy	15	GIT	CBD	1.30 (1.12, 1.51)	0.23 (0.11, 0.34)	11.9548	5.45E-04	1.92	1.48
Trisomy 13	16	Chromosomes	Cannabis	1.22 (1.09, 1.38)	0.18 (0.08, 0.27)	11.7980	3.18E-04	1.75	1.41
Trisomy 21 (Down syndrome)	17	Chromosomes	CBD	1.06 (1.02, 1.09)	0.05 (0.02, 0.09)	9.4889	0.0021	1.30	1.16
Double outlet right ventricle	18	CVS	Cannabis	1.21 (1.07, 1.36)	0.17 (0.06, 0.27)	9.2314	0.0013	1.70	1.34
Clubfoot	19	Limb	CBD	1.10 (1.03, 1.18)	0.09 (0.03, 0.15)	7.9686	0.0048	1.43	1.20
Deletion 22q11.2	20	Chromosomes	Cannabis	1.36 (1.09, 1.68)	0.26 (0.09, 0.41)	7.8339	0.0028	2.05	1.42
Clubfoot	21	Limb	Cannabis	1.07 (1.01, 1.14)	0.07 (0.01, 0.12)	6.0907	0.0077	1.36	1.14
Cleft lip with and without cleft palate	22	Face	CBD	1.52 (1.08, 2.14)	0.34 (0.07, 0.53)	5.8113	0.0159	2.41	1.37
Esophageal atresia/tracheoesophageal fistula	23	GIT	Cannabis	1.11 (1.02, 1.21)	0.10 (0.02, 0.17)	5.4545	0.0112	1.45	1.15
Single ventricle	24	CVS	Cannabis	1.23 (1.03, 1.46)	0.19 (0.03, 0.32)	5.4301	0.0113	1.76	1.22
Biliary atresia	25	GIT	CBD	1.20 (1.02, 1.40)	0.17 (0.02, 0.29)	5.1462	0.0233	1.69	1.18
Biliary atresia	25	GIT	Cannabis	1.19 (1.02, 1.39)	0.16 (0.02, 0.28)	5.0640	0.0141	1.67	1.17
Hypoplastic left heart syndrome	26	CVS	Cannabis	1.10 (1.01, 1.19)	0.09 (0.01, 0.16)	4.8102	0.0164	1.42	1.11
Epispadias	27	GUT	Cannabis	1.31 (1.03, 1.67)	0.24 (0.03, 0.40)	4.7877	0.0166	1.95	1.20
Diaphragmatic hernia	28	Body Wall	CBD	1.09 (1.00, 1.17)	0.08 (0.00, 0.15)	4.3354	0.0373	1.39	1.07
Double outlet right ventricle	29	CVS	CBD	1.16 (1.01, 1.33)	0.14 (0.01, 0.25)	4.3080	0.0379	1.58	1.10
Trisomy 13	30	Chromosomes	CBD	1.14 (1.00, 1.28)	0.12 (0.00, 0.22)	4.1053	0.0427	1.53	1.07
Single ventricle	31	CVS	CBD	1.22 (1.00, 1.50)	0.18 (0.00, 0.33)	3.9021	0.0482	1.75	1.04

Table 14 lists these various CARs by body system. The results are qualitatively similar to those presented in Table 12 but less dramatic.

**Table 14:** Summary Categorical Variables by System

System	No. Anomalies	Total No. Anomalies	% of Total Anomalies
Chromosomes	5	5	100.0%
GIT	5	6	83.3%
GUT	3	7	42.9%
Limb	2	5	40.0%
Body Wall	1	3	33.3%
CVS	5	19	26.3%
Face	2	9	22.2%
CNS	0	7	0.0%
Total	**22**	**61**	**36.1%**

### Detailed analyses of specific congenital anomalies

It is of interest to consider two of these defects in detail by way of example of the kinds of space-time analyses which might be performed to investigate these data in greater detail. This brief analytical discussion is intended to be exemplary rather than exhaustive as a thorough spatiotemporal treatment of all of this material would require a very large undertaking indeed beyond the bounds of the space which is presently available.

### Small intestinal stenosis and atresia (SISA)

**Fig. 16:**
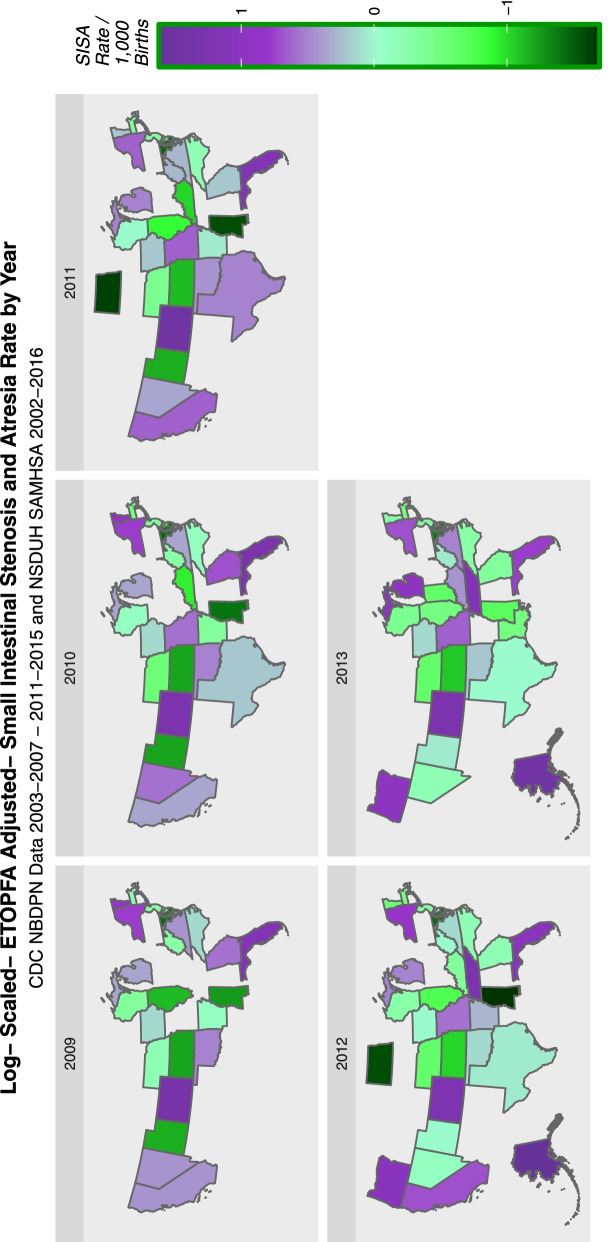
Map-graph of the incidence of small intestinal atresia or stenosis across USA over time

We look first at small intestinal stenosis and atresia (SISA). Figure 16 presents map-graphically the states which provided data for this analysis. SISA is not diagnosed prenatally and is not impacted by ETOPFA practices.

Supplementary Table 13 presents the results of final inverse probability weighted mixed effects models. Interestingly one notes that in these models cannabis and / or cannabinoids are significantly related to SISA incidence. Importantly cannabidiol is independently significantly related and has a positive coefficient in all models in which it appears.

Supplementary Table 14 presents final inverse probability weighted robust generalized linear regression models. Cannabis is significant alone. When all the substances are included in an additive model, only cannabis remains as shown in the second model on this page. In an interactive model with drugs cannabis is again independently significant. In comprehensive additive and interactive models including income and all ethnicities, significant terms including cannabidiol appear in both final models.

Supplementary Table 15 presents the results of inverse probability weighted panel regression models lagging cannabinoids. In both additive and interactive models terms including cannabidiol are significant and have positive coefficients.

States contributing data to the SISA dataset are shown in Supplementary Fig. 9 along with their edited geospatial linkages.

Table 15 presents the results of final geospatial models. Terms including cannabis are positive and significant in all cases.

**Table 15:** Small Intestinal Stenosis or Atresia - Introductory Space – Time Regression Models

Lagged Variables	Parameter			Model Parameters		
	Parameter	Estimate (C.I.)	***P***-Value	Parameter	Value	Parameter ***P***-Value
	***Additive Model - Drugs***			S.D.	0.4633	
	***spreml(Rate ~ Cigarettes + Cannabis + anlyr + Binge.Alcohol + Cocaine)***			LogLik	−112.1308	
	Cannabis	1.15 (0.46, 1.84)	0.0014	psi	0.8736	< 2.2e-16
				lambda	−0.2041	0.04235
	***Interactive Model - Drugs***					
	spreml(Rate ~ Cigarettes * Cannabis * anlyr * Binge.Alcohol + Cocaine)					
	Cigarettes: Cannabis: Binge.Alcohol	57.95 (30.14, 85.75)	4.41E-05	S.D.	0.8069	
	Cannabis: Binge.Alcohol	30.95 (15.37, 46.53)	9.90E-05	LogLik	−100.5249	
	Cigarettes: Cannabis: Binge.Alcohol: Analgesics	11.55 (3.04, 20.06)	0.0078	psi	0.9063	< 2.2e-16
	Cigarettes: Analgesics	−3.12 (−5.07, −1.17)	0.0018	lambda	−0.2276	0.01861
	Cigarettes: Cannabis: Analgesics	−3.96 (−6.04, −1.88)	0.0002			
	Cigarettes: Cannabis	−13.09 (−19.59, −6.59)	7.87E-05			
	***2 Years Lag***					
	***Interactive Model - Drugs***					
	spreml(Rate ~ Cigarettes * Cannabis * anlyr * Binge.Alcohol + Cocaine)					
Cannabis, 2	Cannabis: Analgesics	68.51 (39.94, 97.07)	2.60E-06	S.D.	0.4309	
	Cocaine	−1.36 (−2.18, −0.53)	0.00126	LogLik	−75.0846	
	Cigarettes: Cannabis: Analgesics	−160.88 (−236.65, −85.11)	3.16E-05	psi	0.8940	< 2.2e-16
	Cigarettes: Binge.Alcohol	−159.19 (−224.69, −93.7)	1.90E-06	rho	−0.5234	2.31E-05
	Cannabis: Analgesics: Binge.Alcohol	−170.52 (−233.74, −107.31)	1.24E-07			
	***4 Years Lag***					
	***Interactive Model - Drugs***					
	spreml(Rate ~ Cigarettes * Cannabis * anlyr * Binge.Alcohol + Cocaine)					
Cannabis, 4	Cigarettes: Analgesics	418.42 (221.76, 615.07)	3.04E-05	S.D.	0.4485	
	Cannabis: Analgesics	1284.76 (677.88, 1891.64)	3.34E-05	LogLik	−19.5113	
	Cigarettes	1335.95 (704.65, 1967.25)	3.36E-05	lambda	−0.7130	1.59E-06
	Cannabis	4106.59 (2160.15, 6053.02)	3.55E-05			
	Cigarettes: Cannabis	−17,101.54 (−26,215.01, −7988.07)	0.0002			
	Cigarettes: Cannabis: Analgesics	−5380.17 (−8221.58, −2538.76)	0.0002			
	Analgesics	−101.13 (−144.83, −57.43)	5.73E-06			

Table 16 shows the results of final geospatial models looking at substances using the cannabinoids as covariates. In all cases terms including the cannabinoids are significant. In models lagged at one, two and three years terms including cannabidiol are significant and the coefficients positive.

**Table 16:** Small Intestinal Stenosis or Atresia - Cannabinoid Space – Time Regression Models

Lagged Variables	Parameter			Model Parameters		
	Parameter	Estimate (C.I.)	***P***-Value	Parameter	Value	Parameter ***P***-Value
	***Additive Model - Cannabinoids***					
	***spreml(Rate ~ Cigarettes + THC + CBG + CBD + anlyr + Binge.Alcohol + Cocaine)***					
	CBG	0.96221 (0.28, 1.64)	0.0055	S.D.	0.4323	
	Binge.Alcohol	8.50833 (1.49, 15.53)	0.0175	LogLik	−107.7976	
	THC	−1.57158 (−3.08, −0.06)	0.0416	psi	0.9129	< 2.2e-16
	Cigarettes	−6.73252 (−13.04, −0.43)	0.0363	rho	−0.2431	0.01896
	***Interactive Model - Cannabinoids***					
	spreml(Rate ~ Cigarettes * THC * CBG * CBD + anlyr + Binge.Alcohol + Cocaine)					
	Cigarettes: THC: Binge.Alcohol	5169.433 (3191.79, 7147.08)	3.00E-07	S.D.	0.6566	
	THC	172.247 (93.57, 250.92)	1.78E-05	LogLik	−87.0831	
	Cigarettes	1748.111 (926.38, 2569.84)	3.05E-05	psi	0.9267	< 2.2e-16
	Cigarettes: THC: CBG: Binge.Alcohol	480.252 (250.09, 710.41)	4.32E-05	lambda	−0.2760	0.0039
	Cigarettes: CBG	339.558 (175.8, 503.32)	4.82E-05			
	Binge.Alcohol	1561.587 (780.78, 2342.4)	8.86E-05			
	CBG: Binge.Alcohol	276.267 (124.22, 428.31)	0.0004			
	Cigarettes: CBG: Binge.Alcohol	−1470.381 (−2232.93, −707.83)	0.0002			
	CBG	−63.136 (−95.55, −30.73)	0.0001			
	Cigarettes: THC: CBG	−109.577 (−164.86, −54.3)	0.0001			
	Cigarettes: Binge.Alcohol	−7753.892 (−11,552.85, −3954.94)	6.32E-05			
	THC: Binge.Alcohol	−796.23 (−1149.18, −443.28)	9.79E-06			
	Cigarettes: THC	−1143.639 (−1586.1, −701.18)	4.06E-07			
	***1 Years Lag***					
	***Interactive Model - Cannabinoids***					
	spreml(Rate ~ Cigarettes * THC * CBG * CBD + anlyr + Binge.Alcohol + Cocaine)					
THC, 1	Cigarettes: CBD	510 (212.08, 807.92)	0.0008	S.D.	0.4457	
CBG, 1	Cigarettes: THC: CBD	563 (229.8, 896.2)	0.0009	LogLik	−91.2983	
CBD, 1	Cigarettes: THC: CBG	1770 (513.64, 3026.36)	0.0056	psi	0.8824	< 2.2e-16
	THC	5.51 (0.37, 10.65)	0.0356	lambda	−0.3009	0.0050
	Cigarettes: THC	−25.5 (−50.78, −0.22)	0.0479			
	THC: CBG	−367 (−625.72, −108.28)	0.0054			
	Cigarettes: CBG: CBD	−13,800 (−22,286.8, −5313.2)	0.0014			
	***2 Years Lag***					
	***Interactive Model - Cannabinoids***					
	spreml(Rate ~ Cigarettes * THC * CBG * CBD + anlyr + Binge.Alcohol + Cocaine)					
THC, 2	Cigarettes: CBG	2040.99 (821.21, 3260.77)	0.0010	S.D.	0.4457	
CBG, 2	CBG: CBD	6381.11 (2226.34, 10,535.89)	0.0026	LogLik	−91.2983	
CBD, 2	THC	10.36 (1.06, 19.65)	0.0289	psi	0.8779	< 2.2e-16
	Cigarettes: THC	−44.97 (−88.58, −1.36)	0.0432	lambda	−0.4332	0.0001
	THC: CBG: CBD	−4896.22 (−8596.78, −1195.65)	0.0095			
	CBD	−177.12 (−308.63, −45.6)	0.0083			
	Cigarettes	−70.36 (−115.93, −24.79)	0.0025			
	CBG	−493.37 (−753.84, −232.89)	0.0002			
	***3 Years Lag***					
	***Interactive Model - Cannabinoids***					
	spreml(Rate ~ Cigarettes * THC * CBG * CBD + anlyr + Binge.Alcohol + Cocaine)			S.D.	0.4457	
THC, 3	CBD	3.38 (0.51, 6.26)	0.0211	LogLik	−91.2983	
CBG, 3	Cigarettes: CBD	−16.7 (−29.52, −3.87)	0.0107	psi	0.8615	< 2.2e-16
CBD, 3	Cigarettes	−72.4 (−122.84, −21.96)	0.0049	lambda	−0.3782	0.0162

Table 17 presents a similar analysis this time including all income and ethnicity covariates. In each model terms for the cannabinoids are positive and significant. In each model terms including cannabidiol are also positive and significant.

**Table 17:** Small Intestinal Stenosis or Atresia - Comprehensive Cannabinoid Space – Time Regression Models

Lagged Variables	Parameter			Model Parameters		
	Parameter	Estimate (C.I.)	***P***-Value	Parameter	Value	Parameter ***P***-Value
	***Interactive Model - Including Sociodemographics***					
	spreml(Rate ~ Cigarettes * THC * CBG * CBD + anlyr + Binge.Alcohol + Cocaine + Income + 5_Races)					
	CBG	1.15 (0.45, 1.85)	0.0014	S.D.	0.4457	
	Cigarettes: CBD	1.33 (0.36, 2.3)	0.0071	LogLik	−91.2983	
	Binge.Alcohol	7.21 (0.37, 14.04)	0.0388	psi	0.9046	< 2.2e-16
	THC	−0.98 (−1.96, −0.01)	0.0476	rho	−0.2587	0.01168
	***1 Years Lag***					
	***Interactive Model - Including Sociodemographics***					
	spreml(Rate ~ Cigarettes * THC * CBG * CBD + anlyr + Binge.Alcohol + Cocaine + Income + 5_Races)					
THC, 1	Cigarettes: THC	109.89 (22.86, 196.92)	0.0133	S.D.	0.4457	
CBG, 1	Cigarettes: THC: CBD	24.48 (3.55, 45.41)	0.0219	LogLik	−91.2983	
CBD, 1	THC	−22.12 (−42.11, −2.14)	0.0300	psi	0.8695	< 2.2e-16
	THC: CBD	−5.4 (−10.23, −0.58)	0.0282	rho	−0.3226	0.005233
	CBG	−1.41 (−2.34, −0.48)	0.0030			
	***2 Years Lag***					
	***Interactive Model - Including Sociodemographics***					
	spreml(Rate ~ Cigarettes * THC * CBG * CBD + anlyr + Binge.Alcohol + Cocaine + Income + 5_Races)					
THC, 2	CBD	1 (0.41, 1.6)	0.0009	S.D.	0.4457	
CBG, 2	CBG	1.74 (0.53, 2.94)	0.0046	LogLik	−91.2983	
CBD, 2	THC: CBD	1.75 (0.48, 3.03)	0.0072	psi	0.8514	< 2.2e-16
	THC	5.8 (0.8, 10.8)	0.0231	rho	−0.4179	0.00155

Table 18 collects some of the regression terms from earlier tables and presents their applicable computed E-Values for the inverse probability weighted mixed effects and panel models.

**Table 18:** Small Intestinal Stenosis or Atresia - E-Values from Mixed Effects and Panel Regression Models

Parameter	Estimate (C.I.)	R.R. (C.I.)	E-Values
***MIXED EFFECTS***			
***Cannabis Only***			
Cannabis	2.83 (2.03, 3.63)	5.66 (3.48, 3.19)	10.80, 6.43
***Additive Model – Drugs***			
Cannabis	1.46 (0.69, 2.22)	3.91 (1.93, 7.92)	7.28, 3.27
***Interactive Model – Drugs***			
Cigarettes: Cannabis: Binge.Alcohol	5638.66 (3549.85, 7727.46)	Infinity (Infinity, Infinity)	Infinity, Infinity
Cigarettes: Cannabis: Binge.Alcohol: Analgesics	1797.36 (1122.19, 2472.54)	Infinity (Infinity, Infinity)	Infinity, Infinity
Cannabis: Binge.Alcohol	8008.91 (4682.06, 11,335.77)	Infinity (Infinity, Infinity)	Infinity, Infinity
Cannabis: Binge.Alcohol: Analgesics	2546 (1467.93, 3624.06)	Infinity (Infinity, Infinity)	Infinity, Infinity
***Additive Model – Cannabinoids***			
THC	0.94 (0.41, 1.48)	2.41 (1.47, 3.93)	4.24, 2.31
CBD	0.84 (0.25, 1.43)	2.18 (1.27, 3.74)	3.78, 1.85
***Interactive Model – Cannabinoids***			
CBG: CBD	10.47 (7.47, 13.47)	4.59E+06 (6.01E+04, 3.51E+08)	3.18E+06, 1.20E+05
CBD	34.98 (24.72, 45.24)	1.85E+22 (6.74E+15, 5.11E+28)	3.71E+22, 1.34E+16
THC: CBG: CBD	0.57 (0.37, 0.76)	2.29 (1.73, 3.04)	4.03, 2.87
CBG	32.45 (19.49, 45.41)	2.45E+20 (3.32E+12, 6.15E+28)	3.05E+20, 6.65E+12
Cigarettes: THC: CBD	13.79 (6.1, 21.48)	6.03E+08 (8.99E+06, 4.03E+13)	1.21E+09, 1.80E+04
***Additive Model - Including Sociodemographics***			
THC	1.45 (0.79, 2.12)	3.97 (2.12, 7.41)	7.41, 3.67
CBD	0.81 (0.21, 1.4)	2.145 (1.24, 3.77)	3.74, 1.77
***Interactive Model - Including Sociodemographics***			
CBG	77.88 (58.11, 97.66)	3.15E+38 (7.30E+28, 1.36E+48)	6.30E+38, 1.46E+29
CBD	63.63 (47.13, 80.13)	2.82E+31 (2.57E+23, 3.09E+39)	5.64E+31, 5.15E+23
CBG: CBD	18.44 (13.62, 23.25)	1.29E+09 (5.85E+06, 2.87E+11)	2.59E+09, 1.17E+07
Cigarettes: THC	2351.21 (1186.17, 3516.25)	Infinity (Infinity, Infinity)	Infinity, Infinity
Cigarettes: THC: CBD	548.39 (275.16, 821.63)	2.07E+284 (4.31E+141, Infinity)	Infinity, 8.61E+141
Cigarettes: THC: CBG: CBD	135.08 (66.39, 203.77)	5.92E+66 (2.01E+33, 1.74E+100)	1.18E+67, 4.03E+33
Cigarettes: THC: CBG	575.97 (282.33, 869.61)	1.19E+271 (8.89E+137, Infinity)	Infinity, 1.77E+138
***PANEL MODELS***			
***Additive Model - Including Sociodemographics***			
CBG	1.07 (0.51, 1.63)	3.31 (1.77, 3.17)	6.06, 2.94
CBD	0.61 (0.23, 0.99)	1.97 (1.298, 3.02)	3.36, 1.91
***Interactive Model - Including Sociodemographics***			
Cigarettes: THC	20.18 (10.52, 29.83)	4.31E+28 (9.13E+14, 2.04E+42)	8.63E+28, 1.82E+15
CBG: CBD	0.92 (0.4, 1.44)	20.014 (3.65, 109.74)	39.53, 6.76
CBD	3.68 (1.19, 6.16)	1.65E+05 (49.84, 5.46E+08)	3.30E+05, 99.18
***1 Years Lag***			
Cigarettes: THC	6.68 (3.64, 9.72)	2.42E+06 (70.07, 8.34E+03)	4.83E+03, 139.63

Table 19 performs a similar role for regression terms derived from geospatial models.

**Table 19:** Small Intestinal Stenosis or Atresia - E-Values from Space – Time Regression Models

Parameter	Estimate (C.I.)	R.R. (C.I.)	E-Values
***SPACE-TIME MODELS***			
***Additive Model - Drugs***			
Cannabis	1.15 (0.46, 1.84)	9.60 (2.48, 37.17)	18.70, 4.40
***Interactive Model - Drugs***			
Cigarettes: Cannabis: Binge.Alcohol	57.95 (30.14, 85.75)	2.40E+28 (6.17E+14, 9.36E+41)	4.81E+28, 1.23E+15
Cannabis: Binge.Alcohol	30.95 (15.37, 46.53)	1.44E+15 (3.48E+07, 5.96E+22)	2.88E+15, 6.96E+07
Cigarettes: Cannabis: Binge.Alcohol: Analgesics	11.55 (3.04, 20.06)	4.54E+05 (31.57, 6.55E+09)	9.09E+05, 62.64
***2 Years Lag***			
***Interactive Model - Drugs***			
Cannabis: Analgesics	68.51 (39.94, 97.07)	6.69E+62 (4.80E+36, 9.34E+88)	1.33E+63, 9.61E+36
***4 Years Lag***			
***Interactive Model - Drugs***			
Cannabis: Analgesics	1284.76 (677.88, 1891.64)	Infinity (Infinity, Infinity)	Infinity, Infinity
Cannabis	4106.59 (2160.15, 6053.02)	Infinity (Infinity, Infinity)	Infinity, Infinity
***Additive Model - Cannabinoids***			
CBG	0.96221 (0.28, 1.64)	16.09 (2.45, 105.29)	31.67, 4.35
***Interactive Model - Cannabinoids***			
Cigarettes: THC: Binge.Alcohol	5169.433 (3191.79, 7147.08)	Infinity (Infinity, Infinity)	Infinity, Infinity
THC	172.247 (93.57, 250.92)	4.79E+103 (2.62E+56, 8.74E+150)	9.58E+103, 5.25E+56
Cigarettes: THC: CBG: Binge.Alcohol	480.252 (250.09, 710.41)	1.19E+289 (6.58E+150, Infinity)	Infinity, 1.31E+151
Cigarettes: CBG	339.558 (175.8, 503.32)	2.45E+204 (1.04E+106, 5.80E+302)	Infinity, 2.08E+106
CBG: Binge.Alcohol	276.267 (124.22, 428.31)	1.96E+166 (9.07E+74, 4.26E+257)	Infinity, 1.81E+75
***1 Years Lag***			
***Interactive Model - Cannabinoids***			
Cigarettes: CBD	510 (212.08, 807.92)	Infinity (2.65E+187, Infinity)	Infinity, Infinity
Cigarettes: THC: CBD	563 (229.8, 896.2)	Infinity (1.78E+204, Infinity)	Infinity, Infinity
Cigarettes: THC: CBG	1770 (513.64, 3026.36)	Infinity (Infinity, Infinity)	Infinity, Infinity
THC	5.51 (0.37, 10.65)	7.74E+04 (2.18, 2.74E+09)	1.55E+05, 3.79
***2 Years Lag***			
***Interactive Model - Cannabinoids***			
Cigarettes: CBG	2040.99 (821.21, 3260.77)	Infinity (Infinity, Infinity)	Infinity, Infinity
CBG: CBD	6381.11 (2226.34, 10,535.89)	Infinity (Infinity, Infinity)	Infinity, Infinity
THC	10.36 (1.06, 19.65)	7.65E+09 (10.81, 5.41E+18)	1.53E+10, 21.11
***3 Years Lag***			
***Interactive Model - Cannabinoids***			
CBD	3.38 (0.51, 6.26)	183.44 (2.20, 1.52E+04)	366.39, 3.83
***Interactive Model - Including Sociodemographics***			
CBG	1.15 (0.45, 1.85)	11.34 (2.58, 49.90)	22.17, 4.59
Cigarettes: CBD	1.33 (0.36, 2.3)	16.55 (2.15, 127.21)	32.59, 3.72
***1 Years Lag***			
***Interactive Model - Including Sociodemographics***			
Cigarettes: THC	109.89 (22.86, 196.92)	4.32E+91 (1.62E+19, 1.15E+164)	8.68E+91, 3.25E+19
Cigarettes: THC: CBD	24.48 (3.55, 45.41)	2.57E+20 (985.96, 6.70E+37)	5.14E+20, 1.97E+03
***2 Years Lag***			
***Interactive Model - Including Sociodemographics***			
CBD	1.00 (0.41, 1.60)	6.70 (2.18, 20.54)	12.89, 3.80
CBG	1.74 (0.53, 2.94)	26.83 (2.76, 260.21)	53.17, 4.98
THC: CBD	1.75 (0.48, 3.03)	274.86 (2.47, 313.56)	55.22, 4.38
THC	5.80 (0.80, 10.8)	2.96E+04 (4.60, 7.71E+08)	1.19E+05, 6.68

Supplementary Table 16 lists all 57 of these minimum E-Values in descending order. All 57 are noted to be above the threshold of 1.25, 34 are noted to be greater than 100 and 13 are infinite.

It is of interest to consider predicted values from geospatiotemporal models. For this purpose the comprehensive interactive model shown in Table 17 lagged to two years was chosen.

**Fig. 17:**
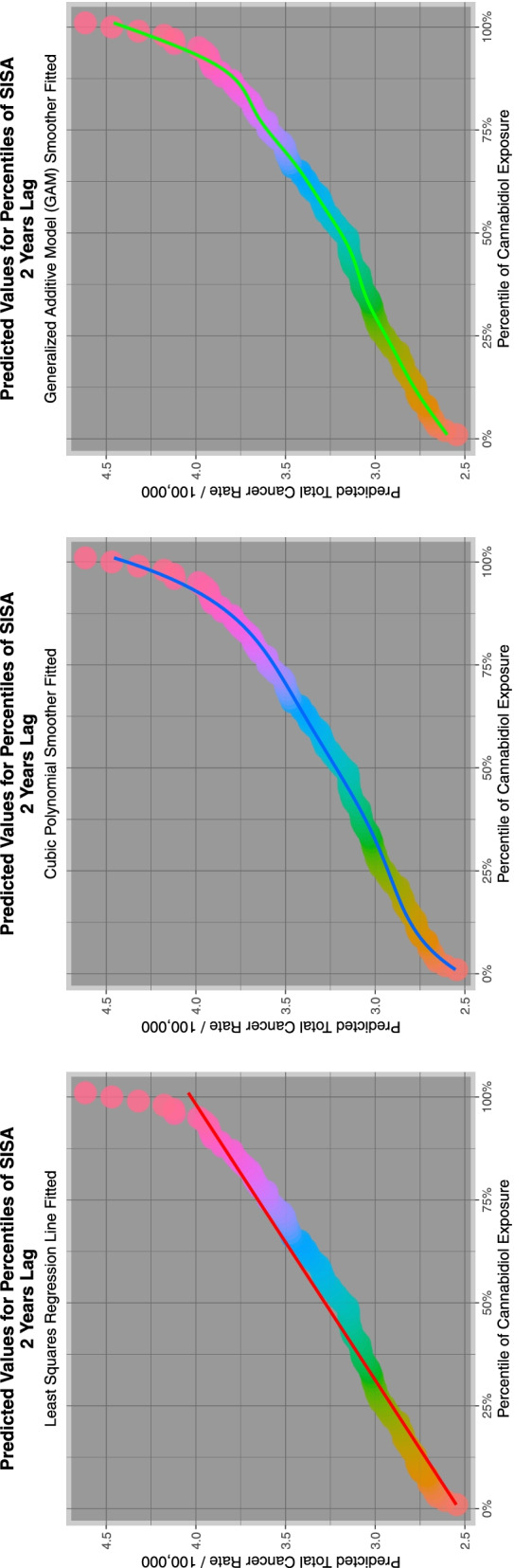
Modelled rates of small intestinal stenosis or atresia rates with rising cannabidiol exposure in a geospatial model lagged to two years

The 101 predicted percentile values from matrix multiplication and scale adjustment are shown graphically in Fig. 17 with least squares regression lines, cubic polynomial and GAM curves are fitted. Percentiles refer to percentiles of cannabidiol exposure. Supplementary Table 17 presents the comparison of the ninetieth and tenth percentiles, the 95th and fifth percentiles and the first and 99th percentiles. An increasing ratio is noted in the right hand column consistent with an increasing effect at higher doses, and the obvious upwards inflection point on the fitted curve.

Supplementary Table 18 presents concisely the results of the various linear, polynomial and GAM regressions. At Anova testing the cubic curve is noted to have a superior fit to the least squares regression line (Anova: F = 365.64, df = 2, 97, *P* = 7.86x10^−47^) and the GAM is also noted to have a superior fit to the least squares line (Anova: F = 265.91, df = 7.89, 91.11, *P* = 2.83x10^−60^). The GAM model was superior to the cubic model (Anova: F = 23.096, df = 5.85, 93.15, *P* = 3.37x10^−16^).

Supplementary Table 19 presents the E-Values which are applicable to these linear regression results. The minimum E-Values are noted to range up to 1.73x10^36^.

As mentioned the abscissa of this regression study was percentiles of cannabidiol exposure. When percentiles of the three cannabinoids THC, cannabigerol and cannabidiol were used instead similar results were obtained particularly with relation to strongly sigmoidal modelled trends (results not shown).

### Obstructive genitourinary defects

**Fig. 18:**
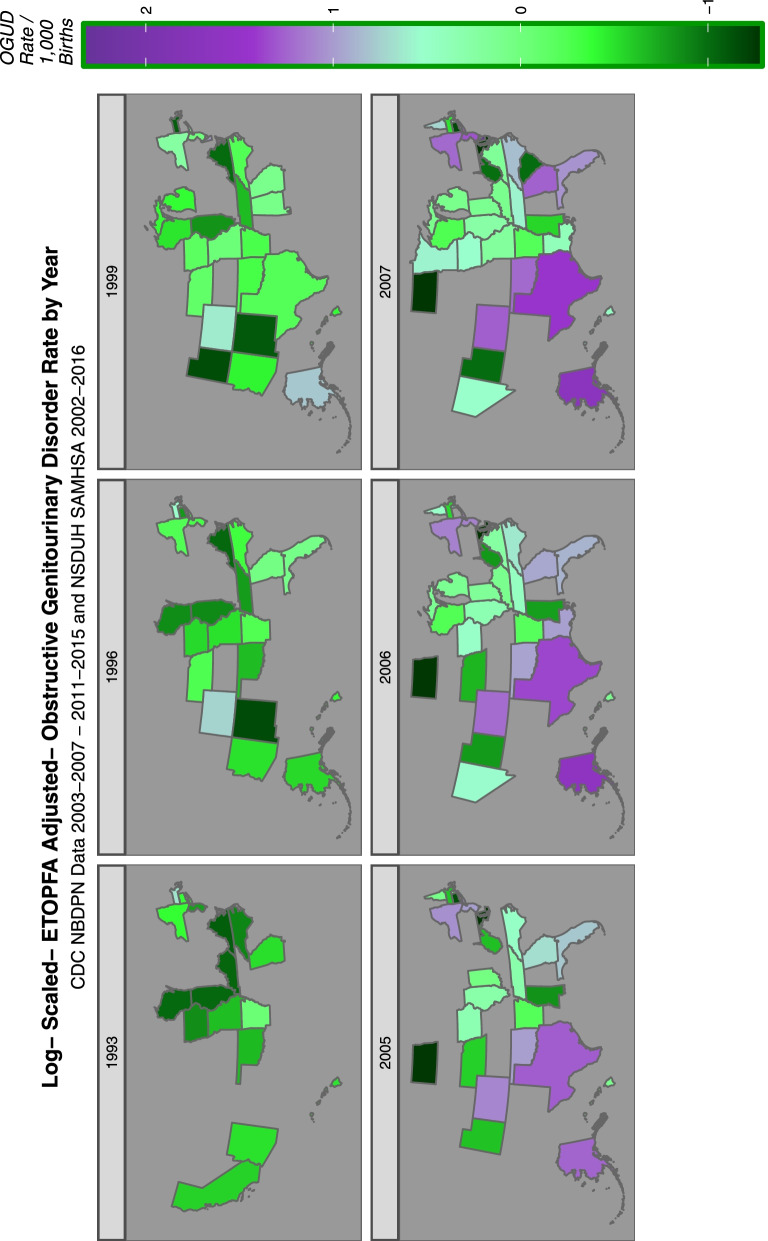
Map-graph of the incidence of obstructive genitourinary defects across USA over time

Figure 18 illustrates states contributing data to the obstructive genitourinary disorder (OGUD) dataset. This disorder is diagnosed prenatally but is not subject to ETOPFA practices.

Supplementary Table 20 presents final inverse probability weighted mixed effects models. Interestingly cannabis is again shown to be the only remaining term in the final additive model for drugs. In the last two models on the comprehensive dataset, the effect of cannabinoids is strongly positive. In the final comprehensive interactive model two significant terms include cannabidiol and have positive β-coefficients.

Final inverse probability weighted robust generalized linear regression models are presented in Supplementary Table 21. In the final comprehensive interactive model shown in this Table two terms for cannabidiol are strongly positive at high levels of statistical significance.

Final comprehensive inverse probability weighted panel regression models for cannabinoids are shown in Supplementary Table 22. Many positive terms for cannabinoids are noted.

Supplementary Fig. 10 illustrates the geospatial linkages which were derived and edited for the OGUD dataset.

Table 20 presents the results of final geospatiotemporal models for OGUD incidence. One notes that cannabis alone is highly signifcant. In an additive model limited to substance covariates, cannabis was the only remaining significant term in the final model. At two years of lag cannabis was again the most significant term. The overall effect of cannabis in this model was positive. The effects of THC, cannabigerol and cannabidiol considered separately were positive in each case.

**Table 20:** Obstructive Genitourinary Defects - Introductory Space – Time Regression Models

Lagged Variables	Parameter			Model Parameters		
	Parameter	Estimate (C.I.)	***P***-Value	Parameter	Value	Model ***P***-Value
	***Additive Model - Drugs***			S.D.	0.2111	
	***spreml(Rate ~ Cigarettes + Cannabis + Analgesics + Bng.Alcohol + Cocaine)***			LogLik	−34.1136	
	Cannabis Alone Significant					
	Cannabis	10.61 (4.7, 16.52)	0.0004	psi	0.9753	< 2.2e-16
	***Interactive Model - Drugs***			S.D.	2.5182	
	spreml(Rate ~ Cigarettes * Cannabis * Analgesics * Bng.Alcohol + Cocaine)			LogLik	−265.2450	
	Cannabis Alone Significant					
	Cannabis	10.61 (4.7, 16.52)	0.0004	psi	0.9752598	< 2.2e-16
	***Interactive Model - Drugs - 1 Years Lag***					
	spreml(Rate ~ Cigarettes * Cannabis * Analgesics * Bng.Alcohol + Cocaine)					
Cannabis, 1	No significant terms remaining in final model					
	***2 Years Lag***					
	***Interactive Model - Drugs***					
	spreml(Rate ~ Cigarettes * Cannabis * Analgesics * Bng.Alcohol + Cocaine)					
Cannabis, 2	Cannabis	241.68 (65.24, 418.12)	0.0073	S.D.	11.2206	
	Cocaine	28.63 (5.32, 51.93)	0.0161	LogLik	−118.9370	
	Cannabis: Bng.Alcohol	−1008.107 (−1720.7, −295.52)	0.0056			
	Bng.Alcohol	−3055.107 (−5206.69, −903.52)	0.0054			
	***THC***			S.D.	2.5182	
	***spreml(Rate ~ THC)***			LogLik	−265.2450	
	THC	8.14 (4.27, 12)	3.78E-05	psi	0.9769	< 2.2e-16
	***Cannabigerol***			S.D.	2.5789	
	***spreml(Rate ~ Cannabigerol)***			LogLik	−270.4920	
	Cannabigerol	7.54 (3.14, 11.94)	7.74E-04	psi	0.9752	< 2.2e-16
	***Cannabidiol***			S.D.	2.7184	
	***spreml(Rate ~ Cannabidiol)***			LogLik	−270.4921	
	Cannabidiol	4.42 (−0.34, 9.18)	0.0687	psi	0.9731	< 2.2e-16
	***Additive Model - Drugs & Cannabinoids***			S.D.	2.5182	
	***spreml(Rate ~ Cigarettes + THC + CBG + CBD + Analgesics + Bng.Alcohol + Cocaine)***			LogLik	−271.5570	
	THC Alone Significant					
	THC	8.14 (4.27, 12)	3.78E-05	psi	0.9769	< 2.2e-16
	***Interactive Model - Drugs & Cannabinoids***			S.D.	2.4848	
	spreml(Rate ~ Cigarettes * THC * CBG * CBD + Analgesics + Bng.Alcohol + Cocaine)			LogLik	−264.4223	
	THC Alone Significant					
	THC	8.14 (4.27, 12)	3.78E-05	psi	0.9768613	< 2.2e-16
	***Interactive Model - Cannabinoids - 1 Years Lag***					
THC, 1	spreml(Rate ~ Cigarettes * THC * CBD + Analgesics + Bng.Alcohol + Cocaine)					
CBD, 1	No significant terms remaining in final model					
	***Interactive Model - Cannabinoids - 2 Years Lag***					
THC, 2	spreml(Rate ~ Cigarettes * THC * CBD + Analgesics + Bng.Alcohol + Cocaine)					
CBD, 2	No significant terms remaining in final model					

Table 21 shows the results of spatial and temporal lagging of cannabinoids. Several terms positive for cannabinoids are evident.

**Table 21:** Obstructive Genitourinary Defects - Cannabinoid Space – Time Regression Models

Lagged Variables	Parameter			Model Parameters		
	Parameter	Estimate (C.I.)	***P***-Value	Parameter	Value	Model ***P***-Value
	***1 Spatial Lag - Interactive Model, Cannabinoids***					
	THC * CBD					
	spreml(Rate ~ Cigarettes * THC * CBD + Analgesics + Bng.Alcohol + Cocaine)					
THC, 2	Cigarettes	2767.39 (1031.71, 4503.07)	0.0018	S.D.	2.4975	
CBD, 2	Cigarettes: CBD	792.04 (292.87, 1291.2)	0.0019	LogLik	−264.8543	
	Cigarettes: THC: CBD	912.27 (282.87, 1541.68)	0.0045	psi	0.9768	< 2.2e-16
	Cigarettes: THC	3167.74 (941.61, 5393.88)	0.0053			
	THC	−712.92 (−1234.34, −191.5)	0.0074			
	THC: CBD	−208.76 (−356.95, −60.56)	0.0058			
	CBD	−188.38 (−305.1, −71.66)	0.0016			
	***1 Spatial Lag - Interactive Model, Cannabinoids***					
	THC * CBG					
	spreml(Rate ~ Cigarettes * THC * CBG + Analgesics + Bng.Alcohol + Cocaine)					
THC, 2	Cigarettes: THC: CBG	855.74 (286.28, 1425.2)	0.0032	S.D.	2.4975	
CBG, 2	Cigarettes: THC	2980.74 (841.4, 5120.08)	0.0063	LogLik	−264.8543	
	Cigarettes	2664.14 (705.35, 4622.92)	0.0077	psi	0.9768	< 2.2e-16
	Cigarettes: CBG	755.71 (171.07, 1340.34)	0.0113			
	THC	−655.51 (−1157.24, −153.79)	0.0104			
	CBG	−185.75 (−327.89, −43.62)	0.0104			
	THC: CBG	−194.05 (−327.64, −60.46)	0.0044			
	***1 Spatial, 1 Temporal Lag - Interactive Model, Cannabinoids***					
	spreml(Rate ~ Cigarettes * THC * CBD + Analgesics + Bng.Alcohol + Cocaine)					
THC, 1	Cigarettes: THC: CBD	1394.48 (386.59, 2402.38)	0.0067	S.D.	2.8611	
CBD, 1	Cigarettes: THC: THC.Spatial: CBD	1384.11 (374.49, 2393.72)	0.0072	LogLik	−189.0979	
THC, 1 Spatial	Cigarettes: THC	5000.58 (1323.37, 8677.78)	0.0077	psi	0.9833	< 2.2e-16
	Cigarettes: THC: THC.Spatial	4975.93 (1182.67, 8769.19)	0.0101			
	Cigarettes	1787.24 (184.11, 3390.37)	0.0289			
	Cigarettes: CBD	522.98 (44.27, 1001.7)	0.0323			
	CBD	−134.19 (−253.84, −14.55)	0.0279			
	THC	−1084.97 (−1955.48, −214.47)	0.0146			
	THC: THC	−1084.66 (−1951.65, −217.67)	0.0142			
	THC: CBD	−311.84 (−552.33, −71.36)	0.0110			
	THC: THC: CBD	−307.33 (−537.59, −77.07)	0.0089			
	***1 Spatial, 2 Temporal Lags - Interactive Model, Cannabinoids***					
	spreml(Rate ~ Cigarettes * THC * CBD + Analgesics + Bng.Alcohol + Cocaine)					
THC, 2	Cigarettes	137,535.9 (58,078.87, 216,992.93)	0.0007	S.D.	9.6638	
CBD, 2	Cigarettes: CBD	48,350.5 (20,095.92, 76,605.08)	0.0008	LogLik	−116.844	
THC, 1 Spatial	Cigarettes: THC	217,699.3 (89,605.46, 345,793.14)	0.0009	rho	−0.68203	0.002462
	Cigarettes: THC: CBD	76,973.5 (31,232.59, 122,714.41)	0.0010			
	THC	11,707.8 (4631.81, 18,783.79)	0.0012			
	THC: THC.Spatial	19,063 (7395.32, 30,730.68)	0.0014			
	THC: CBD	−18,888.3 (−30,246.11, −7530.49)	0.0011			
	Cigarettes: THC: THC	−78,290.4 (−125,332.16, −31,248.64)	0.0011			
	THC	−53,462 (−85,264.37, −21,659.63)	0.0010			
	Cigarettes: THC	−48,251.3 (−76,830.65, −19,671.95)	0.0009			
	CBD	−11,798.1 (−18,785.3, −4810.9)	0.0009			

Table 22 lists final comprehensive interactive and interactive temporally lagged models. All models include positive significant terms for cannabinoids.

**Table 22:** Obstructive Genitourinary Defects - Comprehensive Cannabinoid Space – Time Regression Models

Lagged Variables	Parameter			Model Parameters		
	Parameter	Estimate (C.I.)	***P***-Value	Parameter	Value	Model ***P***-Value
	***Interactive Model - Including Sociodemographics***					
	spreml(Rate ~ Cigarettes * THC * CBD + Analgesics + Bng.Alcohol + Cocaine + Income + 5_Races)					
	Hispanic	7.56 (3.56, 11.55)	0.0002	S.D.	2.3684	
	THC	37.58 (9.36, 65.79)	0.0090	LogLik	−254.1933	
	Am.Indian/Alaskan.Native	124.12 (30.78, 217.46)	0.0092	psi	0.9663	< 2.2e-16
	THC: CBG	6.95 (0.33, 13.56)	0.0395			
	Income	−13.2 (−23.45, −2.94)	0.0117			
	***1 Years Lag***					
	***Interactive Model - Including Sociodemographics***					
	spreml(Rate ~ Cigarettes * THC * CBD + Analgesics + Bng.Alcohol + Cocaine + Income + 5_Races)					
THC, 1	Hispanic	7.59 (3.07, 12.12)	0.0010	S.D.	3.2724	
CBD, 1	Cigarettes: THC	46.25 (16.84, 75.67)	0.0021	LogLik	−187.7251	
	Am.Indian/Alaskan.Native	148.61 (47.3, 249.93)	0.0040	psi	0.9689	< 2.2e-16
	Income	−17.24 (−30.36, −4.12)	0.0100			
	***2 Years Lag***					
	***Interactive Model - Including Sociodemographics***					
	spreml(Rate ~ Cigarettes * THC * CBD + Analgesics + Bng.Alcohol + Cocaine + Income + 5_Races)					
THC, 2	Hispanic	12.81 (8.33, 17.3)	2.17E-08	S.D.	3.2724	
CBD, 2	Cigarettes: THC: CBD	6151.83 (2693.75, 9609.91)	0.0005	LogLik	−187.7251	
	Cigarettes: THC	22,951.53 (9883.29, 36,019.77)	0.0006	psi	0.0000	NA
	Cigarettes	15,335.11 (5177.59, 25,492.63)	0.0031			
	Cigarettes: CBD	4078.6 (1248.29, 6908.9)	0.0047			
	Am.Indian/Alaskan.Native	107.64 (18.06, 197.22)	0.0185			
	CBD	−894.76 (−1549.98, −239.53)	0.0074			
	Bng.Alcohol	−186.96 (−318.68, −55.23)	0.0054			
	THC	−5115.33 (−8111.04, −2119.62)	0.0008			
	THC: CBD	−1370.14 (−2158.79, −581.48)	0.0007			

Table 23 lists the E-Values derived from mixed effects and panel regression models and Table 24 shows those derived from spatiotemporal models.

**Table 23:** Obstructive Genitourinary Defects - E-Values from Mixed Effects and Panel Regression Models

Parameter	Estimate (C.I.)	R.R. (C.I.)	E-Values
***MIXED EFFECTS MODELS***			
***Cannabis Only***			
Cannabis	14.35 (8.44, 20.27)	94.85 (15.13, 594.66)	189.20, 29.75
***Additive Model - Drugs***			
Cannabis	14.35 (8.44, 20.27)	94.85 (15.13, 594.66)	189.20, 29.75
***Interactive Model - Drugs***			
Cigarettes: Cannabis: Analgesics	333.48 (176.14, 490.83)	1.62E+51 (4.54E+27, 5.81E+74)	3.25E+51, 9.09E+27
Cannabis: Bng.Alcohol: Analgesics	700.3 (368.06, 1032.54)	3.47E+107 (6.41E+57, 1.88E+157)	6.94E+107, 1.29E+58
Cannabis: Bng.Alcohol	921.93 (370.26, 1473.6)	3.75E+1241 (9.87E+58, 1.43E_224)	7.51E+141, 1.97E+59
***Additive Model - Cannabinoids***			
THC	43.47 (8.06, 78.89)	1.72E+06 (18.61, 1.59E+11)	3.43E+06, 36.72
***Interactive Model - Cannabinoids***			
Cigarettes: THC	1945.4 (832.31, 3058.5)	2.73E+296 (5.73E+130, Infinity)	Infinity, 1.14E+131
Cigarettes: THC: CBG	482.22 (204.16, 760.28)	3.02E+73 (1.23E+32, 7.37E+114)	6.04E+73, 2.46E+32
***Additive Model - Including Sociodemographics***			
THC	11.62 (7.82, 15.42)	58.96 (16.01, 217.10)	117.42, 31.52
***Interactive Model - Including Sociodemographics***			
THC: CBG	918.55 (286.58, 1550.52)	2.27E+138 (4.07E+45, 1.27E+231)	4.55E+138, 8.15E+45
THC: CBG: CBD	248.54 (72.69, 424.4)	2.73E+37 (4.24E+11, 1.76E+63)	5.46E+37, 8.49E+11
THC	3517.29 (910.69, 6123.89)	Infinity (1.78E+147, Infinity)	Infinity, 3.57E+147
THC: CBD	946.55 (214.43, 1678.68)	3.75E+142 (1.34E+35, 1.05E+250)	7.51E+142, 2.69E+35
***PANEL MODELS***			
***Interactive Model - Including Sociodemographics***			
THC	7726.08 (3068.06, 12,384.1)	Infinity (9.29E+186, Infinity)	Infinity, Infinity
THC: CBD	2899.61 (1040.91, 4758.31)	1.47E+176 (2.93E+63, 7.39E+288)	Infinity, 5.86E+63
THC: CBG: CBD	202.14 (44.72, 359.56)	1.91E+12 (545.0179, 6.69E+21)	3.82E+12, 1.09E+03
***Sociodemographic Interactive Model - 1 Lag***			
Cigarettes: THC: CBD	163.56 (80.31, 246.82)	4.75E+08 (1.85E+04, 1.21E+13)	9.50E+08, 3.71E+04
Cigarettes: THC	719.66 (347.71, 1091.61)	1.50E+38 (3.05E+18, 7.39E+57)	3.01E+38, 6.11E+18
***Additive Model - Drugs***			
Cannabis	10.61 (4.7, 16.52)	5.66E+19 (8.37E+08, 3.82E+30)	1.13E+20, 1.67E+09
***Interactive Model - Drugs***			
Cannabis	10.61 (4.7, 16.52)	44.75 (5.40, 370.45)	89.06, 10.29
***Interactive Model - Drugs, 2 Lags***			
Cannabis	241.68 (65.24, 418.12)	3.25E+08 (204.43, 5.18E+14)	6.51E+08, 408.35
***THC***			
THC	8.14 (4.27, 12)	19.67 (4.78, 80.93)	38.84, 9.03
***Cannabigerol***			
Cannabigerol	7.54 (3.14, 11.94)	14.30 (3.04, 67.26)	28.10, 5.53
***Additive Model - Drugs & Cannabinoids***			
THC	8.14 (4.27, 12)	18.91 (4.68, 76.34)	37.31, 8.84
***Interactive Model - Drugs & Cannabinoids***			
THC	8.14 (4.27, 12)	19.67 (47.78, 80.94)	38.84, 9.04
1 Spatial Lag - Interactive Model, THC * CBD			
Cigarettes: CBD	792.04 (292.87, 1291.2)	2.15E+125 (3.19E+46, 1.45E+204)	4.31E+125, 6.39E+46
Cigarettes: THC: CBD	912.27 (282.87, 1541.68)	2.29E+144 (9.107E+44, 5.77E+243)	4.58E+144, 1.83E+45
Cigarettes: THC	3167.74 (941.61, 5393.88)	Infinity (5.16E+149, Infinity)	Infinity, 1.03E+150
1 Spatial Lag - Interactive Model, THC * CBG			
Cigarettes: THC: CBG	855.74 (286.28, 1425.2)	8.09E+135 (4.45E+45, 1.47E+226)	1.61E+136, 8.91E+45
Cigarettes: THC	2980.74 (841.4, 5120.08)	Infinity (2.06E+134, Infinity)	Infinity, 4.14E+134
Cigarettes: CBG	755.71 (171.07, 1340.34)	1.05E+120 (2.27E+27, 4.83E+212)	2.09E+120, 4.54E+27
***1 Spatial, 1 Temporal Lag Cannabinoids***			
Cigarettes: THC: CBD	1394.48 (386.59, 2402.38)	4.17E+192 (4.79E+53, Infinity)	Infinity, 9.59E+53
Cigarettes: THC: THC.Spatial: CBD	1384.11 (374.49, 2393.72)	1.53E+1981 (1.02E+52, Infinity)	Infinity, 2.05E+52
Cigarettes: THC	5000.58 (1323.37, 8677.78)	Infinity (6.65E+183, Infinity)	Infinity, Infinity
Cigarettes: THC: THC.Spatial	4975.93 (1182.67, 8769.19)	Infinity (2.62E+164, Infinity)	Infinity, Infinity
Cigarettes: CBD	522.98 (44.27, 1001.7)	1.74E+72 (1.77E+06, 1.70E+138)	3.47E+72, 3.54E+06
***1 Spatial, 2 Temporal Lags Cannabinoids***			
Cigarettes: CBD	48,350.5 (20,095.92, 76,605.08)	Infinity (Infinity, Infinity)	Infinity, Infinity
Cigarettes: THC	217,699.3 (89,605.46, 345,793.14)	Infinity (Infinity, Infinity)	Infinity, Infinity
Cigarettes: THC: CBD	76,973.5 (31,232.59, 122,714.41)	Infinity (Infinity, Infinity)	Infinity, Infinity
THC	11,707.8 (4631.81, 18,783.79)	Infinity (1.01E+190, Infinity)	Infinity, Infinity
THC: THC.Spatial	19,063 (7395.32, 30,730.68)	Infinity (2.51E+303, Infinity)	Infinity, Infinity
***Interactive Model - Including Sociodemographics***			
THC	37.58 (9.36, 65.79)	1.86E+06 (37.31, 9.29E+10)	3.72E+06, 74.13
THC: CBG	6.95 (0.33, 13.56)	14.44 (1.14, 1852.37)	28.36, 1.54
***Sociodemographic Interactive, 1 Lag***			
Cigarettes: THC	46.25 (16.84, 75.67)	3.85E+05 (109.80, 1.35E+09)	7.71E+05, 219.10
***Sociodemographic Interactive, 2 Lags***			
Cigarettes: THC: CBD	6151.83 (2693.75, 9609.91)	1.63E+301 (1.72E+132, Infinity)	Infinity, 3.45E+132
Cigarettes: THC	22,951.53 (9883.29, 36,019.77)	Infinity (Infinity, Infinity)	Infinity, Infinity
Cigarettes: CBD	4078.6 (1248.29, 6908.9)	5.02E+199 (2.51E+61, Infinity)	Infinity, 5.02E+61

**Table 24:** Obstructive Genitourinary Defects - E-Values from Space-Time Regression Models

Parameter	Estimate (C.I.)	R.R. (C.I.)	E-Values
***Additive Model – Drugs***			
Cannabis	10.61 (4.7, 16.52)	5.66E+19 (8.37E+08, 3.82E+30)	1.13E+20, 1.67E+09
***Interactive Model – Drugs***			
Cannabis	10.61 (4.7, 16.52)	44.75 (5.40, 370.45)	89.06, 10.29
***Interactive Model - Drugs, 2 Lags***			
Cannabis	241.68 (65.24, 418.12)	3.25E+08 (204.43, 5.18E+14)	6.51E+08, 408.35
***THC***			
THC	8.14 (4.27, 12)	19.67 (4.78, 80.93)	38.84, 9.03
***Cannabigerol***			
Cannabigerol	7.54 (3.14, 11.94)	14.30 (3.04, 67.26)	28.10, 5.53
***Additive Model - Drugs & Cannabinoids***			
THC	8.14 (4.27, 12)	18.91 (4.68, 76.34)	37.31, 8.84
***Interactive Model - Drugs & Cannabinoids***			
THC	8.14 (4.27, 12)	19.67 (47.78, 80.94)	38.84, 9.04
1 Spatial Lag - Interactive Model, THC * CBD			
Cigarettes: CBD	792.04 (292.87, 1291.2)	2.15E+125 (3.19E+46, 1.45E+204)	4.31E+125, 6.39E+46
Cigarettes: THC: CBD	912.27 (282.87, 1541.68)	2.29E+144 (9.107E+44, 5.77E+243)	4.58E+144, 1.83E+45
Cigarettes: THC	3167.74 (941.61, 5393.88)	Infinity (5.16E+149, Infinity)	Infinity, 1.03E+150
1 Spatial Lag - Interactive Model, THC * CBG			
Cigarettes: THC: CBG	855.74 (286.28, 1425.2)	8.09E+135 (4.45E+45, 1.47E+226)	1.61E+136, 8.91E+45
Cigarettes: THC	2980.74 (841.4, 5120.08)	Infinity (2.06E+134, Infinity)	Infinity, 4.14E+134
Cigarettes: CBG	755.71 (171.07, 1340.34)	1.05E+120 (2.27E+27, 4.83E+212)	2.09E+120, 4.54E+27
***1 Spatial, 1 Temporal Lag Cannabinoids***			
Cigarettes: THC: CBD	1394.48 (386.59, 2402.38)	4.17E+192 (4.79E+53, Infinity)	Infinity, 9.59E+53
Cigarettes: THC: THC.Spatial: CBD	1384.11 (374.49, 2393.72)	1.53E+1981 (1.02E+52, Infinity)	Infinity, 2.05E+52
Cigarettes: THC	5000.58 (1323.37, 8677.78)	Infinity (6.65E+183, Infinity)	Infinity, Infinity
Cigarettes: THC: THC.Spatial	4975.93 (1182.67, 8769.19)	Infinity (2.62E+164, Infinity)	Infinity, Infinity
Cigarettes: CBD	522.98 (44.27, 1001.7)	1.74E+72 (1.77E+06, 1.70E+138)	3.47E+72, 3.54E+06
***1 Spatial, 2 Temporal Lags Cannabinoids***			
Cigarettes: CBD	48,350.5 (20,095.92, 76,605.08)	Infinity (Infinity, Infinity)	Infinity, Infinity
Cigarettes: THC	217,699.3 (89,605.46, 345,793.14)	Infinity (Infinity, Infinity)	Infinity, Infinity
Cigarettes: THC: CBD	76,973.5 (31,232.59, 122,714.41)	Infinity (Infinity, Infinity)	Infinity, Infinity
THC	11,707.8 (4631.81, 18,783.79)	Infinity (1.01E+190, Infinity)	Infinity, Infinity
THC: THC.Spatial	19,063 (7395.32, 30,730.68)	Infinity (2.51E+303, Infinity)	Infinity, Infinity
***Interactive Model - Including Sociodemographics***			
THC	37.58 (9.36, 65.79)	1.86E+06 (37.31, 9.29E+10)	3.72E+06, 74.13
THC: CBG	6.95 (0.33, 13.56)	14.44 (1.14, 1852.37)	28.36, 1.54
***Sociodemographic Interactive, 1 Lag***			
Cigarettes: THC	46.25 (16.84, 75.67)	3.85E+05 (109.80, 1.35E+09)	7.71E+05, 219.10
***Sociodemographic Interactive, 2 Lags***			
Cigarettes: THC: CBD	6151.83 (2693.75, 9609.91)	1.63E+301 (1.72E+132, Infinity)	Infinity, 3.45E+132
Cigarettes: THC	22,951.53 (9883.29, 36,019.77)	Infinity (Infinity, Infinity)	Infinity, Infinity
Cigarettes: CBD	4078.6 (1248.29, 6908.9)	5.02E+199 (2.51E+61, Infinity)	Infinity, 5.02E+61

These 47 E-Values are listed in descending order in Supplementary Table 23. All 47 are noted to be above 1.25, 36 are noted to be above 100 and nine are noted to be infinite.

It is of interest to consider the way in which rising levels of cannabidiol might impact these results. The model chosen was the first comprehensive interactive model shown in Table 21 lagged to two years. Percentiles refer to percentiles of cannabidiol exposure.

**Fig. 19:**
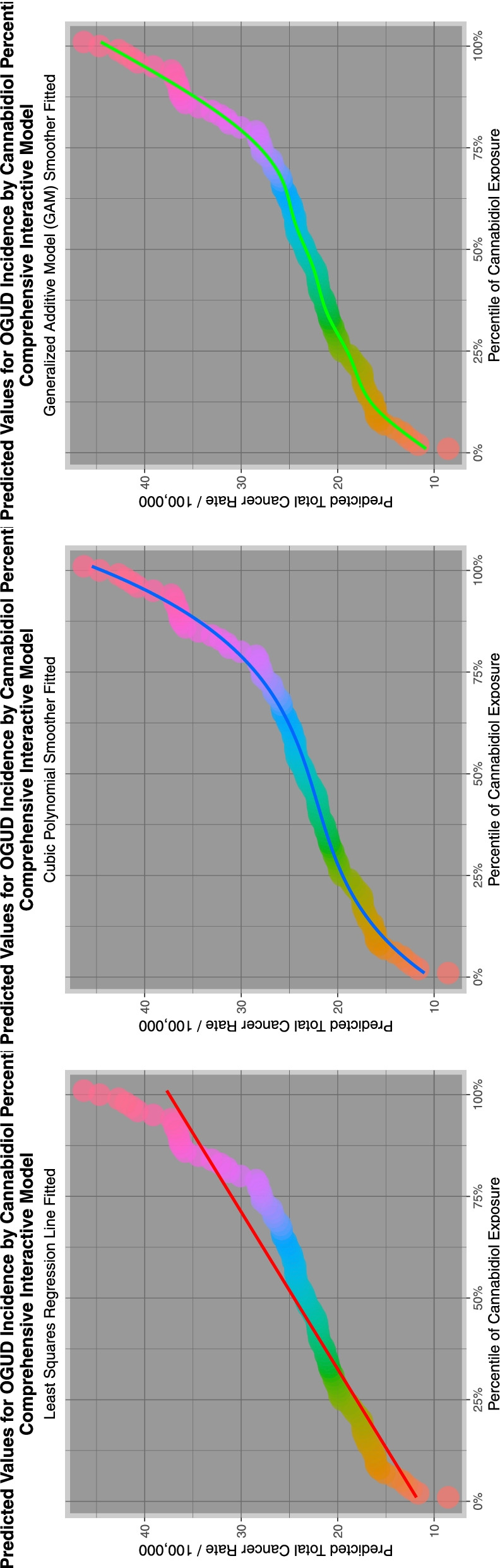
Modelled rates of obstructive genitourinary defect with rising cannabidiol exposure in a geospatial model lagged to two years

The results of matrix multiplication and scale revision are shown in Fig. 19 with least squares regression lines, cubic polynomial and GAM curves fitted. Percentiles are compared in Supplementary Table 24 and one again notes an increasing ratio reflecting the obvious inflection points in the fitted curves. Regression summaries for these three smoothers are shown in Supplementary Table 25. At Anova testing both the cubic polynomial (Anova: F = 499.86, df = 2, 97, *P* = 5.82x10^−51^) and the GAM curve (Anova: F = 172.08, df = 7.7934, 91.207, *P* = 1.61x10^−71^) are noted to be superior to the least squares regression line confirming the significance of the inflection points in the curves.

The E-Values from the two linear regression models are shown in Supplementary Table 26 and their minima are noted to range up to 8.36x10^41^ in the case of the cubic polynomial curve.

When this exercise was repeated for this congenital anomaly including percentiles of THC and cannabigerol in addition to cannabidiol exposure, again the sigmoidal non-linear shape of the fitted curve was strongly confirmed (results not shown).

## Discussion

### Main results

The overall picture to emerge from this national state level survey of cannabinoid teratogenesis confirms and extends the Hawaiian study of 2007 [13] in preference to the “standard model” of cannabinoid and cannabidiol teratogenesis widely canvassed in the medical profession. These findings support the genotoxic warnings placed by national regulatory agencies on approved cannabinoid products including cannabidiol.

The main outcome from this USA teratological survey and overview is that cannabis, THC, cannabidiol and cannabigerol have highly significant associations with congenital anomaly rates whether considered as continuous variables by regression line slope or categorical variables by comparing extreme quintiles and are accompanied by highly significant prevalence ratios, attributable fractions in the exposed, population attributable risks, significance levels and E-values. For the continuous variable analysis 28 of the 41 CAs listed in Table 11 have minimum E-Values greater than 9.0 which is the very high value found in the tobacco-lung cancer relationship [86]. As judged by the number of ETOPFACARs impacted this putative teratogenic effect is greater for THC (40 CAs) than for cannabis (35 CAs) than for tobacco (11 CAs). For cannabidiol (11 CAs) this effect is greater than either last month alcohol consumption (5 CAs) or binge alcohol consumption (2 CAs). For two CAs considered in detail by spatiotemporal analysis and the formal techniques of causal inference, namely small intestinal stenosis or atresia and obstructive genitourinary defects, there is clear epidemiological evidence of both close association across time and space which persists after full model adjustment, and of a causal relationship with cannabinoid including cannabidiol exposure. Moreover predictive modelling from selected spatiotemporal models demonstrates that the relationship between rising cannabidiol exposure and CA incidence is strongly sigmoidal in that both fitted curves show obvious strong positive inflections in their upper ranges which is closely and strongly reminiscent of the exponential dose-response curves observed in the laboratory in numerous genotoxic and mitochondriopathic assays [21, 24, 26, 31, 42, 54–65, 87]. *P*-values for this non-linearity are 2.83x10^−60^ and 1.61x10 ^−71^ respectively. For these CAs minimum polynomial E-Values for the predictive percentile models range up to 1.73x10^36^ and 8.36x10^41^.

The slope of the bivariate relationship between estimates of the ETOPFA-corrected CA incidence rate and the rate of substance exposure for many anomalies is significantly elevated for cannabis, THC and cannabidiol. As shown in Table 2 35 ETOPFA-corrected congenital anomalies have elevated minimum E-values by cannabis exposure regression slope which comprise nine cardiovascular anomalies, six anomalies of the urinary tract, five anomalies of the gastrointestinal tract, all five chromosomal anomalies, four limb musculoskeletal anomalies, two each of face and body wall anomalies and one brain anomaly. For 28 of these 35 anomalies the minimum E-Value is greater than 9.0. The forty CAs with elevated E-values after THC exposure may be grouped as ten cardiovascular CAs, six gastrointestinal CAs, six CAs of the urinary tract, all five chromosomal CAs, five CAs of the facial structures, four CAs of limb development including limb deficiencies and leg reductions, two central nervous system CAs including encephalocele and spina bifida without anencephalus, and two CAs of the body wall development diaphragmatic hernia and omphalocele (Supplementary Table 6).

The twelve ETOPFACARs with elevated E-Values from regression slopes after cannabidiol exposure include small and large intestinal esophageal and biliary atresias and stenoses, hip dislocation, obstructive genitourinary anomalies, and diaphragmatic herniae, cleft palate, reduction deformity of legs and transposition of the great arteries. Obstructive genitourinary defect, esophageal, small and large intestinal and biliary atresias and stenoses, diaphragmatic hernia, Hirschsprungs disease and hip dislocation have elevated E-Values when cannabidiol is considered as both continuous and categorical variables (Tables 3 and 5). For nine of these 12 CAs the minimum E-Value is greater than 18 (Table 3).

Tables 2 and 4 list the CAs with elevated E-Values when cannabis is treated as a continuous and as a categorical variable respectively. The defects which appear on both lists are the chromosomal anomalies Trisomies 13, 18 and 21 (Downs syndrome) and Deletion 22q11.2; the gastrointestinal anomalies esophageal atresia, small intestinal atresia or stenosis, biliary atresia and Hirschsprung disease; the cardiovascular defects hypoplastic left heart syndrome, coarctation of the aorta and pulmonary valve atresia or stenosis; the limb defects congenital hip dislocation and clubfoot, the body wall defect diaphragmatic hernia, and the urological disorder congenital posterior urethral valve.

### Interpretation

Hence these data show not only close association between cannabinoid exposure and various CAs but clearly indicate the existence of a threshold effect above which the teratogenic impact dramatically increases, closely mirroring in patterns of human disease the amply documented threshold effects seen in cellular, molecular, genotoxic and epigenotoxic laboratory studies [21, 24, 26, 31, 42, 54–65, 87].

The present study is intended to be introductory and pathfinding in the sense that its methods are not widely deployed across the published literature of the clinical teratological disciplines and we are keen to see advanced statistical methods more widely utilized to study the important questions raised by this study. However it is also true that sufficient evidence has been presented in the above material to enable several conclusions to be made definitively. Cannabinoid genotoxicity as tracked across multiple congenital anomalies is clinically significant and of public health importance and concern. Cannabis and cannabidiol test strongly positive on the bivariate results presented and are each implicated in more congenital anomalies than either tobacco or alcohol respectively both legal drugs which are widely acknowledged to be toxic to the developing foetus. Based on the very elevated minimum E-Values ofound cannabidiol is also a clinically significant teratogen and presumptive genotoxin and is more potent than either binge alcohol consumption or last month alcohol use. For selected congenital anomalies cannabinoid teratogenicity persists after multivariable adjustment in inverse probability weighted models of causal inference, and after consideration in their inherently space-time context. For both congenital anomalies studied in detail spatiotemporal modelling shows strong evidence of a threshold effect above which the impacts of cannabidiol and cannabinoid teratogenicity are supra-linear, sigmoidal and greatly amplified.

These findings lead to the sobering conclusion that cannabinoid genotoxicity is of great public health importance to maternal-foetal and reproductive medicine in contrast to the fact that it appears to be largely missing from public health discourse to date where it is essentially overlooked.

Moreover given that the prevalence of cannabis use and cannabinoid exposure in the global community is clearly rising increasing cannabinoid exposure will not be related in simple linear fashion to increased congenital anomalies across a wide spectrum of developmental disorders, but the non-linearity of the relationship and the existence of clear thresholds for genotoxicity both in the laboratory and across diverse human communities (in USA as a whole and in Hawaii, Colorado, Canada and Australia [13, 17–20]) implies that a much greater incidence of clinical teratogenesis might reasonably be expected to accompany this increased use, as was indeed recently demonstrated nationwide in USA for atrial septal defect secundum type [16] and for autism [66, 88] and has also recently been demonstrated in Canada and Australia [17–19]. This was also recently confirmed for all five chromsomal disorders reported across USA [89].

The present report is preliminary in the sense that a wider detailed geotemporospatial and causal inference study of many other congenital anomalies is clearly indicated. At the time of writing this more comprehensive and detailed manuscript is in preparation. Our unpublished findings are that such upper range predicted curve positive inflections and sigmoidality are typical and normative amongst geospatial models for almost all positively impacted congenital anomalies studied to date. Also strongly indicated are geotemporospatial studies at finer geospatial resolution such as was recently published from CDC for gastroschisis at county level and which employed similar prevalence ratio methodology to the present study [90].

One notes also that the USA is moving relatively rapidly into an era when cannabinoids are more widely available than previously as the legislative regimes relating to cannabis are progressively relaxed. The replacement of tobacco crops in many places with hemp crops implies that cannabinoids of various forms will increasingly enter the food chain both explicitly as lollies, candies, chocolates, sauces, health foods and oils, and implicitly as stock feed, bird feed and in dairy and egg products. It therefore seems inevitable in such a paradigm that population level cannabinoid exposure will necessarily increase. In this context the traditional way of doing teratological studies by simply asking a binary question as to maternal antenatal exposure to cannabis becomes increasingly inaccurate and passé. Calls for a quantitative biomarker of cannabinoid exposure have been issued derived potentially from epigenomic and / or glycomic metrics [91]. As we enter an era of more widespread known and unknown cannabinoid exposure in the community, higher level cannabinoid potency, higher intensity cannabis use and the widespread availability of highly concentrated cannabinoid oils, dabs, waxes, shatters, extracts and products it seems that the urgency of deriving such a quantitative biomarker necessarily proportionately increases. An important corollary of the deployment of such an objective biomarker is that much smaller numbers of maternal-foetal pairs can be used to measure effect sizes and the chance of mis-attribution is potentially greatly reduced with the added advantage for analysis and for statistical power that cannabinoid exposure can be treated more properly as a continuous variable.

### Mechanistic considerations

#### Role of morphogen gradients in body pattern formation

The gradients of various key morphogens control of the formation of the body in many respects [92]. This is well illustrated in the case of the neural tube which goes to form the spinal cord and central nervous system. Bone morphogenetic proteins and Wnts are released from the dorsal roof plate region in high concentration. Sonic hedgehog (shh) is released form the notochord and induces shh release form the ventral floorplate of the neural tube in high concentration [92]. Hence between the dorsal roof plate and the ventral floor plate there exist opposing and antagonistic gradients from BMPs and Wnts dorsally as against shh ventrally. Shh suppresses class I factors (Pax-3/7, Dbx-1, Dbx-2, Irx3 and Pax-6) and stimulates class II factors (Foxa-2, Nkx-6.2, Nkx-6.1, Olig-2, Nkx-2.2 and Nkx-2.9). These opposing gradients specify in detail the nature of the neurons which will develop in the various loci of the developing neural tube. At the same time lateral gradients of retinoic acid emanate from the lateral edges of the neural tube descending to very low concentrations along the lumen of the neural tube. Rostral-causal axial differentiation is controlled by opposing gradients of retinoic acid rostrally competing with FGF and Gli1 from the caudal end of the neural tube [92].

Hence in a very real way one could say that the structures of the neural tube are actually woven together by opposing and antagonistic but balanced morphogen gradients. Similar principles often operate in numerous other tissues at the level of the overall body pattern, at the organ level, for body rotation where it is not symmetrical, and at the cellular and subcellular levels.

In considering the impacts of cannabinoids on the forming embryo it is of interest to consider the effects cannabinoids might have on one of the main morphogen systems in the body which is sonic hedgehog. A brief consideration of their impacts on other fundamental morphogen systems follows.

#### Sonic Hedgehog

Sonic Hedgehog (shh) is one of the most important of all the body morphogens. Indeed one contemporary textbook includes 174 references to this key morphogen [92].

Shh has been shown to be critically involved in the development of the following structures [92]:

- Gastrula / Early Embryo
  - Primitive node of the late gastrula
  - Notochord
  - shh gradient along ventral surface of embryo
  - Gradient antagonizes its opposing morphogens, particularly FGFs, from posterior embryo

- Brain
  - Early Forebrain specifier and organizer
  - Controls ventral midbrain formation including the ventral tegmental area and Nucleus Accumbens
  - Cerebellum organizer – The large Purkinje cell secrete shh which stimulates granule cell proliferation [92]
  - Induces motor neuron development in the ventral neural tube [92]

- Face
  - Face organizer [92]
  - Shh is critical for the outgrowth of the Palatal shelves
  - Ectodermal tips of the facial processes
  - Controls midline tongue fusion
  - Controls development of the filiform papillae on the tongue
  - Controls tooth development
  - Controls taste bud development
  - Apical ectoderm of second pharyngeal pouch [92]

- Eyes
  - Splits the single eye field into two halves, right and left [92]
  - Induces the outgrowth of the optic cup from the forebrain which becomes the optic nerve and then the optic vesicle and later neural retina
  - The bulging frontal lobe of the forebrain secretes shh to induce an ectodermal organizing centre in the overlying skin called the frontonasal ectodermal zone which controls the development of the cheeks and nose again by the secretion of shh
  - Induction of the ventral and nasal retinae of the eye
  - Acts as a repulsive signal guiding axonal growth of retinal ganglion cells
  - Retinal patterning [92]

- Ears
  - Ear specification – shh specifies ventrality in the developing otocyst [92]

- Mouth
  - Controls mouth formation and size of mouth [92]
  - Breaks down the oropharyngeal membrane

- Respiratory
  - Tips of outgrowing lung buds [92]

- Gastrointestinal Tract
  - Upper and lower Intestinal portals [92]
  - Controls specification of the foregut
  - Shh secreted from the esophageal mucosa control radial specification of the esophagus and inhibits muscle development in the submucosa,
  - Shh signalling from the gastric mucosa controls smooth muscle development
  - Gastric development and enlargement [92]
  - Shh secreted from the intestinal mucosa control radial specification of the intestinal and inhibits muscle development in the submucosa,
  - The muscularis mucosae of the small intestine develops much later in foetogenesis when the shh gradients have declined
  - Intestinal elongation
  - Controls the activity of the gut stem cells deep in the intestinal crypts
  - Rostral and caudal intestinal portals
  - Controls the development of the anal opening
  - Controls pancreas development [92]

- Cardiac
  - Maintains cardiogenic proliferation in the secondary heart field [93]
  - The shh-dependent secondary heart field contributes to the conoventricular outflow tract [94]
  - Shh controls elongation of the conoventricular outflow tract via shh-dependent progenitors [94]
  - Shh is essential for aortic arch development [95]
  - Shh control outflow tract development [96, 97]
  - Shh is critical in cardiovascular development [98]
  - Shh plays a critical role in neural crest cell specification some of which contribute to cardiac cells [99]

- Vascular
  - Induces formation of the dorsal aortae [100]
  - Controls formation and remodelling of branchial arch blood vessels [101]
  - Together with BMP and notch signalling shh is critically involved with induction of the first dedicated haemopoietic cells which arise in the fusing dorsal aortae
  - Arterial differentiation is induced in a molecular cascade which commences with shh signaling to VEGFA and notch from a general endothelial background of angioblasts [92, 102, 103]
- Genitourinary
  - Contributes to bladder growth and sufficiency [92]
  - Contributes as a trophic factor to development and outgrowth of the genital tubercle under the influence of shh derived from the urethral endoderm [92]
- Limbs
  - Zone of polarizing activity in limb formation [92]
  - Key organizer of the patterning of the digits [92]
  - Hair buds development

Therefore the recent demonstration therefore that cannabidiol and THC inhibit shh signalling *necessarily* carries major implications for cannabinoid-related teratogenesis [42]. These cannabinoids were noted to both depress shh and Gli1 mRNA and induce the formation of a CB1R-smoothened (“smoothened” is the effector molecule of the shh “patched” receptor) heteromer which reverses the polarity of downstream signalling of smoothened. These authors noted that the critical period for foetal development in this regard is the third to fourth week of gestation in the embryonal period of development when many women are unaware that they are pregnant.

Interference with shh-dependent processes at key stages of development will likely result in the following anomalies which have been described in various studies as being cannabis-related:

- Exencephaly [11, 104]
- Encephalocele [13, 17]
- Deficiencies in spinal column formation – myelocele and meningomyelocele [13],
- Mental deficiencies such as ADHD and autism spectrum from deficient forebrain differentiation [10, 66, 88, 105]
- Lowered tone and motor control as has been described in children experiencing prenatal cannabinoid exposure [10, 106–109]
- Impaired visuomotor and executive processing seen in PCE children [110–112]
- Cleft lip and palate (USA- present study)
- Holoprosencephaly [42] including cyclopia (single eye) (USA- present study)
- Respiratory [18, 20]
- Limb defects [11–13, 18, 20, 104, 113] (USA- present study)
- Vascular catastrophes – in limbs [13] (USA- present study), body wall closure [7, 8, 13, 114–118]
- Epispadias, hypospadias [20] (USA- present study)
- Obstructive Genitourinary defect (USA- present study)
- Gastrointestinal stenoses and atresias (USA- present study)
- Anorectal agenesis

It has been reported by many investigators that cannabinoids reduce cell growth and reduce synthesis of the macromolecules of life such as DNA, RNA and proteins including histones [12, 23, 24, 26–32, 119–122].

The inhibition of cell growth and division would explain many features of cannabis teratogenesis including:

i) Failure of the anterior and posterior neuropores to close, resulting in encephalocele, exencephaly and spina bifida respectively;
ii) Cleft lip and palate due to failure of the facial and palatal processes to properly fuse
iii) Several cardiovascular defects including:
  - Atrial septal defect secundum, where the atrial septal folds fail to grow across the defect
  - Ventricular septal defects where the various components of the ventricular wall fail to join across the defect
  - Stenoses and atresias of the heart valves
  - Defective development of the great vessels, which have a very complex developmental course
iv) Body wall defects
v) Limb defects, where failure or interruption of cell division at key period of limb bud outgrowth interrupts the normal sequence of events required for normal limb development affecting:
  - The whole limb
  - The upper or lower segments of the limb
  - Digital development of fingers and toes
vi) Gastrointestinal stenoses and atresias including:
  - Esophageal atresia [7] (USA- present study)
  - Small intestinal stenosis and atresia (USA- present study)
  - Large intestinal stenosis and atresia (USA- present study)
  - Biliary stenosis and atresia (USA- present study)
  - Anorectal stenosis and atresias (USA- present study)
vii) Arterial vascular catastrophes
  - Limb development
  - Body wall – omphalocele, gastroschisis, diaphragmatic hernia

As shown above shh is known to be a key morphogen directing the differentiation of the arterial tree and its inhibition can be expected to disrupt normal vasculogenic and arterial supply of key tissues. Cannabinoids are also vasoactive [123]. Both type 1 and 2 cannabinoid receptors (CB1Rs and CB2Rs) along with other receptor subtypes have been described on the vasculature [123]. Cannabinoids acting at CB1Rs are often proinflammatory and vasoconstrictive [123–127]. Such vascular defects could be involved with the genesis of various congenital anomalies including:

i) Body wall defects (gastroschisis and omphalocele) – cocaine and various vasoconstrictive antihistaminic drugs are known to be associated with gastroschisis [128–133] and cannabinoids may act similarly at least in the foetal period of development
ii) Gastrointestinal stenoses and atresias
iii) Limb development as the developing limb anlage is highly vascular dependent any interruption of its blood supply will necessarily truncate development.

Hence it could be said that the full spectrum of cannabinoid-induced embryopathy follows to a close approximation a picture of shh mutation or deficit. The point has previously been made that embryonic shh deficiency causes a wide variety of congenital defects including effects on vertebra, anal atresia, cardiovascular anomalies, tracheoesophageal fistula, renal defects and limb defects (VACTERL syndrome) [134]. These defects also have similarities both to fetal alcohol syndrome [42] and Di George / Velocardiofacial (palatocardiofacial) syndrome which may also include kidney and intellectual problems [135].

### Other genotoxic mechanisms

In addition to direct and indirect interactions with specific morphogen pathways cannabinoids have also been shown to interact deleteriously with chromosomes, DNA, the epigenome and mitochondrial-metabolic-epigenomic pathways. These are reviewed in a companion manuscript and have been considered elsewhere [18–20, 24, 28, 31, 37, 38, 41, 91, 113, 136–142].

### Specific organ systems

#### Heart

In Hawaii five cardiovascular defects were related to elevated cannabis use, atrial and ventricular septal defects, pulmonary valve atresia and stenosis, tetralogy of Fallot and hypoplastic left heart syndrome [13]. In Colorado four cardiovascular defects rose across time with increasing community cannabinoid penetration, namely atrial septal defect, ventricular septal defect, patent ductus arteriosus and anomalies of the pulmonary artery [20]. In Canada total cardiovascular defects were related to increased cannabis use [18]. In Australia total cardiovascular defects, atrial and ventricular septal defects, transposition of the great arteries, tetralogy of Fallot and patent ductus arteriosus occurred with higher incidence in high cannabis using areas [19]. They also featured prominently in the present US overview.

It is important to appreciate that heart development occurs by including cells from many loci in the embryo including the primary and secondary heart fields, proepicardium, Juxtacardiac field [143], cardiac neural crest and neural crest [92].

Major morphogens acting are retinoic acid, FGFs and shh. Neuregulin is involved in the induction of both the heart valves and also the subendocardial electrical conducting system of the heart [92].

It therefore follows that heart and great vessels form as a result of a carefully orchestrated sequential complementation of progenitor cells from many areas, some quit remote from the cardiogenic field itself [92]. It is also apparent that numerous genes and transcription factors are involved in this process [92].

Given the wide diversity of cannabinoid actions in a wide variety of cell types it seems particularly unlikely that cannabinoids would not impact this delicate and intricate process at many points.

The numerous interactions of shh with both heart and great vessel formation were enumerated above.

#### Respiratory defects

Respiratory defects were noted to be elevated in the high cannabis using areas of Colorado and Canada [18, 20]. Shh is noted to be centrally involved in the budding and development of the respiratory tree [92].

#### Face

In the Hawaiian series incidence rates of cleft lip and palate together with anotia / microtia were elevated by prenatal cannabis exposure [13]. Microphthalmia was non-significantly elevated. In Canada facial clefts were non-significantly elevated [18]. In Australia facial and ear anomalies were non-significantly elevated [19].

As was noted above shh plays a large role in face development through the frontal facial organizer, at the tip of the frontonasal processes which form the sides of the cleft lip, at the tips of the palatal shelves, in the tongue, teeth, taste buds and filiform papillae [92].

Alcohol and steroidal alkaloids are known to disrupt shh signalling in the face [144].

#### Gastrointestinal tract

The Hawaiian series noted that several gastrointestinal anomalies were elevated following prenatal cannabis exposure including esophageal atresia, pyloric stenosis, and large bowel stenoses and atresias including anorectal atresia [13]. In Australia small intestinal stenosis was identified positively [19]. Gastrointestinal anomalies featured prominently in the present analysis including particularly small intestinal stenosis and atresia which was linked with cannabidiol use both causally and in a space-time context.

The prominent involvement of shh and major morphogens in the growth and development of all parts of the gastrointestinal tract was described above [92].

#### Urinary tract

Given the above notes on the location of shh in the genitourinary system it is of interest that obstructive genitourinary defects were identified both in Hawaii and in the present US survey series [13]. Hypospadias was identified positively in Australia [19].

#### Body wall anomalies

Gastroschisis and diaphragmatic hernia have previously been noted to be linked with prenatal cannabis exposure by CDC and NBDPN researchers [7] although gastroschisis was not positively identified in the present investigation [7]. In Colorado gastroschisis and diaphragmatic hernia were positively identified [20].

#### Limbs

Limb reductions were noted as significant correlates in the continuous bivariate analysis of THC and cannabis with minimal E-Values of 1.89 and 9.53. Leg reductions were noted as significant correlates of cannabidiol, THC and cannabis with minimal E-Values of 2.38, 1.32, and 2.57 (Tables 6, 7, 8). They were not seen in association with tobacco, alcohol or cocaine exposure. This finding is consistent with the arm reduction anomalies reported from Hawaii following prenatal cannabis exposure [13], the elevation of total congenital anomalies seen in Canada which also may have included limb reductions [18] and preclinical studies [11, 12, 104]. Cannabis of course is well known to interfere with both cellular division including macromolecular synthesis and blood vessel sprouting. Blood vessels are known to have high density cannabinoid receptors which are known to be frequently pro-inflammatory and vasoactive [123–127]. Moreover limb outgrowth occurs in a tight time window during embryogenesis [145]. It is therefore possible that cannabinoid exposure during this critical window of development interferes with cellar division in the limb bud and vascular budding and outgrowth thereby compromising limb development.

It is of interest that arm reduction anomalies along with polydactyly and syndactyly were noted to have occurred with increased incidence rates following prenatal cannabis exposure in the Hawaiian series, and leg anomalies rates rose in the present US series [13]. Polydactyly and syndactyly and total musculoskeletal anomalies rose in Colorado with cannabis legalization [20]. It is difficult to comment on the major limb anomalies as it is a congenital anomaly for which ETOPFA may be practised at high rates. In the Australian series there was a non-significant trend to higher rates of major arm and leg anomalies in the high cannabis using areas [19]. Similarly outbreaks of major limb anomalies were noted in both France and Germany [45, 47, 48, 50] in recent years where cannabinoids have been allowed to enter the food chain, but not in nearby Switzerland where this is not permitted.

Major morphogens involved in early limb development are opposing gradients of the Fibroblast Growth Factors (FGF) and Wnt on the one hand and retinoic acid on the other. Limb length is controlled by Hox genes D-9 to D-13. Specification and formation of the fingers and toes is controlled by alternating interactions and gradients between sonic hedgehog, gremlin and FGF4 and by manipulating these gradients and gene dosages experimentally one is able to control various malformations in a predictable manner [145].

It is of interest therefore that there are at least three major pathways by which cannabinoids can interfere with limb bud development and outgrowth:

i) Direct inhibition of cell division and cell growth
ii) Direct and indirect blockades of shh gradients from the zone of polarizing activity in the inferior axillary region and along the posterior edge of the limb and in the digital rays
iii) Vasculopathic mechanisms whereby interference with the ingrowing blood supply compromises limb development.

It is important to note that limb development is strictly sequential so that a block at critical developmental time periods will inevitably block subsequent steps. It is easy to appreciate in such a paradigm that significant cannabinoid intake in such critical windows of gestation may have potentially catastrophic implications for limb growth and development.

It is also noteworthy that cannabis shares many of the mechanisms of action of thalidomide [146–152] an agent which is notorious for interfering with limb outgrowth and bony skeletal development, albeit at higher potency [53, 146, 151, 153–156].

#### Chromosomal defects

Downs syndrome was identified positively in Hawaii, Colorado, Australia and Canada as well as in the present analysis of both categorical and continuous ETOPFA-corrected data [13, 18–20]. Chromosomal defects were found to be elevated in Canada and Australia [18, 19] as well as in the present US survey.

Several mechanisms of indirect chromosomal clastogenicity and DNA breakage have been described [24, 26, 28, 33, 138].

### Interactions of cannabinoids with other major morphogen systems

Interaction between FGF (Fibroblast Growth Factor) and endocannabinoid systems have also been described [157, 158] including transactivation of the FGF1R by CB1R [159].

Interactions between cannabinoids and bone morphogenetic proteins have also been described [160–162].

Interactions between cannabinoids and retinoic acid signalling have been described [163–165].

Interactions between cannabinoids and notch signalling have also been reported [166–172].

Interactions between cannabinoids and Wnt signalling have also been reported [173–179].

Interactions between cannabinoids and hippo have been reported [140].

Cannabinoids also interact with the neurexin-neuroligin system [180–182] which is key to the architecture and development of neural synapses.

Cannabinoids also interact with the slit-robo system [168, 169, 183] which control arterial pathfinding and also axonal growth cone steering mechanisms [92, 171, 184, 185]. Slit-robo signalling is also one of the major morphogens directing and controlling the exuberant outgrowth of the massive human neocortex [183, 186].

### Commonality

Given this plethora of actions of actions between cannabinoids and the major morphogens of human and mammalian development one might well wonder why such anomalies are not becoming much more common. There are several parts to this answer. One factor is that the birth defect data from states where cannabis is legal such as Washington state and Oregon are almost non-existent. Data from Colorado shows a dramatic rise in congenital anomalies across the period of legalization as has been mentioned elsewhere [20]. Also since cannabinoids are involved in virtually every aspect of reproduction including gamete formation and meiotic divisions, the function of supporting granulosa and Sertoli cells in ovary and testis, cells placentation, implantation, sperm fertility and hyperactivation, ovarian signals to the sperm and cell division at the early zygote, morula and embryonic stages a high rate of foetal loss is expected from severe anomalies which does not necessarily appear on lists of birth defects, but is chronicled in case series such as that described above from Washington D.C [14, 15]. Moreover the actual state level ETOPFA rate likely varies from place to place and this is a major determinant of the rates of many serious CAs.

### Causal assignment

Two of the commonest criticisms made of observational studies are that the exposure of interest is not distributed randomly across all experimental subjects, and that there may be some uncontrolled confounding operating from some unmeasured variables which account for the observed effect and for which the observed variables are acting merely as surrogates or substitute markers.

The first criticism is answered in the present study by the use of inverse probability weighting of the exposed groups. It is well established that the use of this procedure across observations transforms a merely observational dataset into a pseudo-randomized one from which causal conclusions can properly be drawn by comparing exposure groups. This technique is particularly suitable for those comparisons which would not generally be ethical to apply in randomized controlled studies, such as antenatal exposures.

The second criticism is addressed herein by the use of E-Values. E-Values, or expected values, calculate the degree of correlation required of some unknown confounding variable with both the exposure and the outcome to explain away the observed effect. The literature mentions that values above 1.25 are generally considered to indicate causal effects [67]. The E-value for the lung cancer – tobacco relationship is 9 which is considered high [67, 68, 86]. It is clear from the present study that many of the E-Values quoted are much higher than this gold standard metric.

Moreover it is entirely proper to use E-Values freely in relation both to specific models (which have model standard deviations) and to final predictive models as has been done in the present report [69].

One also notes that for two congenital anomalies we have conducted multiple regression by several techniques which have very similar conclusions. Moreover for these defects we have shown in their intrinsic natural space-time context that these relationships are conserved and indeed amplified.

Furthermore our results are also consistent with a long, robust and highly consistent tradition of laboratory and preclinical evidence as noted above.

As judged by the criteria of causation proposed by Hill [187] the present results fulfil the criteria of strength of association, consistency across studies in the manner described, specificity amongst substance exposures, temporality of sequence, coherence with known data, biological plausibility as described in the above mechanistic discussion, biological dose-response curve, analogy with similar situations in other places and experimental confirmation.

### Generalizability

The present study has several advantages. Its study subject is a sizeable base population comprising a national census birth population in excess of 18 million births, from a notional year-on-year aggregated annualized total population of over 2 billion persons. Drug use data is taken from a well verified nationally representative survey which has been faithfully repeated annually for several decades now with very little important change which greatly facilitates comparison between periods. Advanced statistical methods are employed on both the aggregate dataset of all defects and two congenital anomalies in particular. The techniques both of formal space-time analysis and of causal inference have been utilized. For these reasons internal to the study we are confident that the present work is widely applicable across the globe. Results reported herein strongly indicate that in those third world nations where cannabis is known to be much more widely used the results are expected to be much more severe than those reported for this nation where historically cannabis use was relatively restricted until recent years.

The demonstration that many of these effects give the appearance on bivariate analysis of being truly causal also necessarily implies that the results are truly biological and widely generalizable.

The present work is also entirely consistent with a large and growing external body of evidence from particular states within USA, namely Colorado and Hawaii [13, 20] and also from Australia and Canada which attest to the concordance with the findings reported herein [17–19].

Another important body of work which supports the present results is the preclinical literature which the present results closely replicate. As was noted above in fact virtually all of the mentioned congenital anomalies have been positively identified in the present study.

Hence for this variety of both internal and external reasons we feel that the findings in the present study are widely generalizable with the primary caveat that in nations where cannabis is more widely available we believe that the findings would be of even greater concern in those cases where reliable datasets exist for its accurate assessment.

### Strengths and limitations

In considering the strengths and limitations of the present study it is important to clarify exactly what this study is and what it is not. The present study sets out to present a broad overview of the apparent relationship of the US teratological experience to substance exposure in the population during the notional period 2005–2013 when both major datasets are available. It goes on to explore two particular anomalies in detail from both a causal inference and geotemporospatial perspective as examples of the manner in which such analyses can be carried forward using more versatile analytical techniques on extent data series. For these reasons we feel it is premature to propose a list of cannabinoid related congenital anomalies and limit ourselves merely to noting that the issue is of considerable concern and well warrants further advanced statistical, epidemiological and basic science investigation. Thus our study is not the last word on US substance-related teratology, but in that it applies a series of advanced sequential linear and predictive modelling and sophisticated analytical space-time and causal inferential techniques our study is more like the first word opening an important discussion which has not been well addressed in recent years.

This study has several strengths including using a nationwide census database for congenital anomalies, using a large well validated nationally representative sample of the non-institutionalized US population, using the major techniques of quantitative causal inference namely inverse probability weighting and E-values, and geospatial regression across space and time simultaneously to assess these roles, and continues by studying the predicted values from space-time models to examine the way in which increasing cannabidiol exposure can be related spatiotemporally to increasing dose-effect relationships. The analytical techniques featuring linear models in tidy format conducted serially on 62 congenital anomalies in purrr allow direct comparison of models within the same statistical run. The use of multi-facetted plots allow the direct visual comparison of the effect on multiple congenital anomalies to be visually inspected at a glance, and similarly between plot comparisons allows the effects of various environmental teratogens to be directly compared. Graphical presentations of E-Values also allow the quantitative and causal significance of findings between substances to be directly compared.

The limitations of this study relate to the limitations of its design. In common with most epidemiological studies individual patient level exposure data was not available to it. Obvious ways in which the present work might be extended such as by increasing the geospatial resolution of the work and by increasing the numbers of congenital anomalies for which detailed regression results are presented are outside the ambit of the present study, and represent a fertile area for future workers. NBDPN may be able to further extend the dataset by completing missing data fields. Moreover perhaps the most definitive technique by which to study these data would include the use of inverse probability weighting in spatiotemporal models. It may become possible with time to employ a weighting term which is actually a product of two lists of weights, one being a sparse geospatial matrix and one being IPW, similar to a current implementation in the R “survey” package. Since such techniques have not been developed at the time of writing it has not been possible to deploy them on these topics. In their stead multiple IPW causal models have been used to address pseudo-randomization and complete these gaps. This also represents an important area for future statistical methodological development. As the USA moves increasingly towards population wide exposure to cannabinoids the importance of quantifiable continuous measures of exposure to various cannabinoids, for example by epigenomic and or glycomic criteria proportionately increases as has previously been noted [91]. State level anomaly-specific ETOPFA rates were not available to this work and ETOPFA rates had to be estimated from the published literature. Their addition to the present dataset would improve the quality and accuracy of the various estimates used.

## Conclusion

In summary we note that bivariate analysis of ETOPFA-corrected CA incidence against state-based substance exposure rates indicates that cannabis and estimated THC are more important environmental teratogens than tobacco, and cannabidiol is likely more important in these metrics than either binge or regularly consumed alcohol. Elevated E-values for many defects indicates that a causal relationship is likely. Small intestinal stenosis and atresia and obstructive genitourinary defects were studied in detail by inverse probability weighted mixed effects, robust and panel regression and by space-time regression and by predictive modelling in spatiotemporal models where these findings were all strongly confirmed and again were shown to be epidemiologically causal in nature. Results are consistent and concordant with several decades of preclinical and laboratory work implicating cellular pathways at chromosomal, genomic, epigenomic and mitochondriopathic levels and with interruption of major embryonal-foetal morphogen gradients particularly sonic hedgehog and with patterns of fetotoxicity and embryotoxicity observed in preclinical models and fulfil the Hill criteria of causality. The present work is part of an on-going project to further investigate these themes in greater depth and finer detail. Further work by interested groups in related areas is strongly indicated.

The present situation where cannabidiol is widely available across USA and popularly perceived as harmless is unusually uninformed and particularly ill-advised. Our analyses implicate THC, cannabigerol and cannabidiol, and analyses could be presented similarly implicating also cannabinol and cannabichromene. From a public health point of view the present de facto policy of official negligence is at once unjustified and unjustifiable.

Data indicate that cannabinoid teratogenicity including cannabidiol teratogenicity and presumptive genotoxicity are clinically significant and carry far-reaching and multi-generational public health impacts in foetal-maternal and reproductive medicine. We feel that it is important that the transgenerational impacts of general register-wide overviews and surveys such as this be given wide canvas and discussion in the community and assume substantial prominence in the public debate on the proper and proven role of cannabinoids in the global community. Moreover the assignment of proper weight to inheritable considerations is essential to optimally formulate policy which balances the risk-benefit equation relating to the general widespread distribution of known genotoxins such as numerous cannabinoids – including cannabidiol - as indeed genotoxicity and fetotoxicity has always been a foundational cornerstone and was always the conceptual origin of modern drug regulation by national Government agencies.

## Supplementary Information

**Additional file 1.**

**Additional file 2.**

**Additional file 3.**

## Data Availability

All data generated or analysed during this study are included in this published article and its supplementary information files. Data has been made publicly available on the Mendeley Database Repository and can be accessed from this URL 10.17632/w6ks529sxd.1.

## Abbreviations

AFE: Attributable fraction in the exposed
BMP: Bone morphogenetic proteins
CA: Congenital anomaly
CBC: Cannabichromene
CBD: Cannabidiol
CBG: Cannabigerol
CBN: Cannabinol
CDC: Centers for disease control, Atlanta, Georgia
cGAS: Cyclic GMP-AMP Synthase
Dbx: Double homeobox
DEA: Drug enforcement agency
ETOPFA: Early termination of pregnancy for anomaly
ETOPFACAR: Early termination of pregnancy for anomaly -adjusted congenital anomaly rate
E-Value: Expected value
FVV: Fitted values
FGF: Fibroblast growth factor
Fox: Forkhead box
GAM: Generalized additive model
Gli1: Glioma-associated protein 1
IPW: Inverse probability weighting
NBDPN: National birth defects prevention network
Nkx: Homeobox protein Nkx
NSDUH: National survey of drug use and health
OGUD: Obstructive genitourinary defect
OLS: Ordinary least squares
PAR: Population attributable risk
Pax: Paired box
plm: Panel linear model
PR: Prevalence ratio
RDAS: Restricted-use data analysis system
re: Random effects
SAMHDA: Substance use and mental health data archive
SAMHSA: Substance abuse and mental health services administration
sem: Spatial error method
semsrre: Spatial error method, serial autocorrelation and random effects
sf: Simple features (Package in R)
SISA: Small intestinal stenosis and atresia
Shh: Sonic hedgehog
splm: Spatial panel linear model
spreml: Spatial panel random effects maximum likelihood
SPDSST: Spatial panel dataset in space-time
sr: Serial correlation
STING: Stimulator of interferon genes
THC: Δ9-Tetrahydrocannabinol
VEGFA: Vascular endothelial growth factor A
