# Supplementary material for: Geotemporospatial and causal inference epidemiological analysis of US survey and overview of cannabis, cannabidiol and cannabinoid genotoxicity in relation to congenital anomalies 2001–2015
Source: BMC Pediatr. 2022 Jan 19;22:47. doi: 10.1186/s12887-021-02996-3 (PMC8767720; doi:10.1186/s12887-021-02996-3)
Supplement: Supplementary file 2 — Additional file 2. [file 12887_2021_2996_MOESM2_ESM.pdf]

Log (ETOPFA−Corrected Defect Rates) by Defect Type by Monthly Binge Alcohol Exposure, USA,
Data: NBDPN, CDC Data 1989−1990 − 2011−2015 and NSDUH SAMHDA RDAS SAMHSA

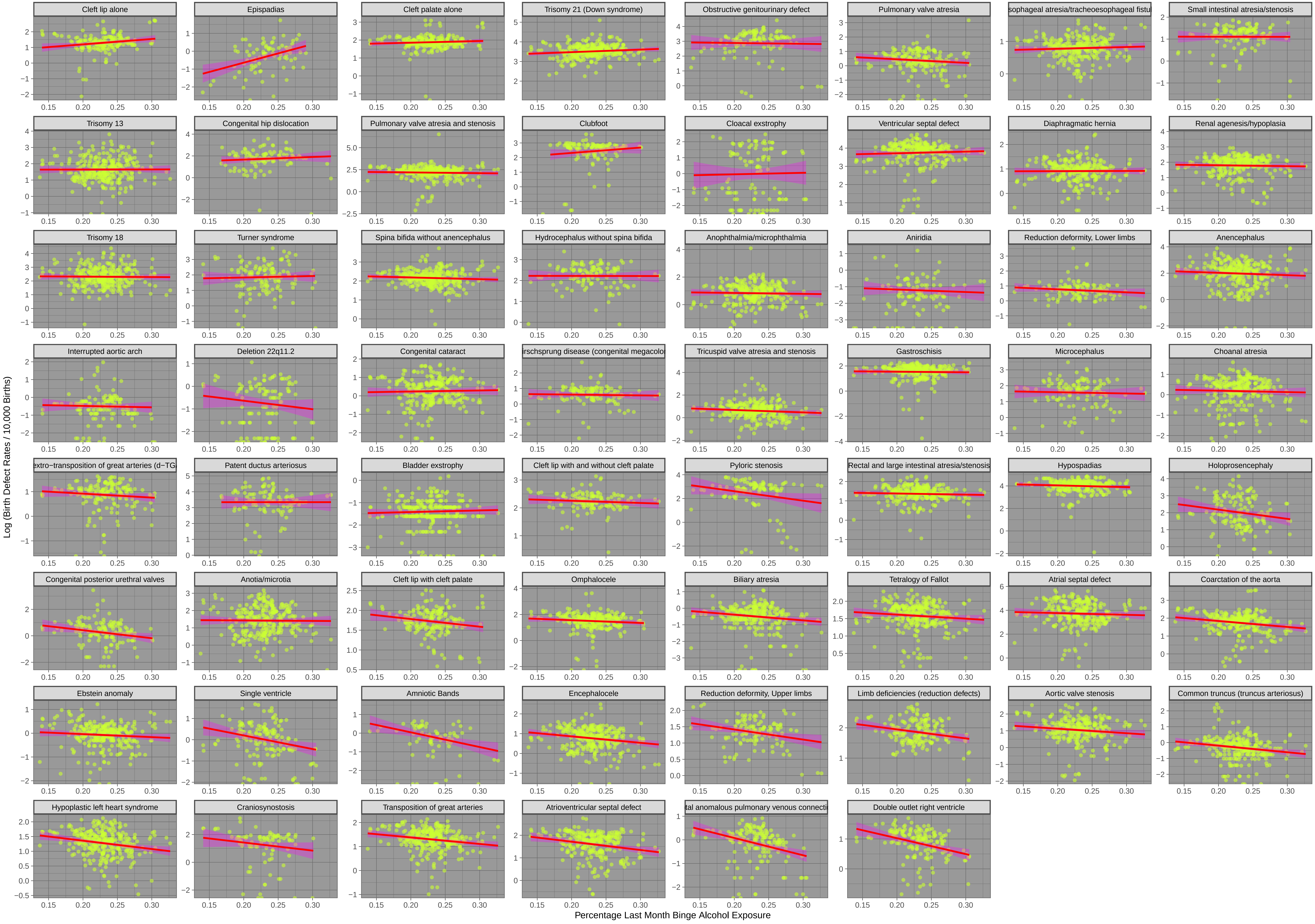

Log (ETOPFA−Corrected Defect Rates) by Defect Type by Monthly Alcohol Exposure, USA,
Data: NBDPN, CDC Data 1989−1990 − 2011−2015 and NSDUH SAMHSA 1999−2015 sas7bdat Datafile

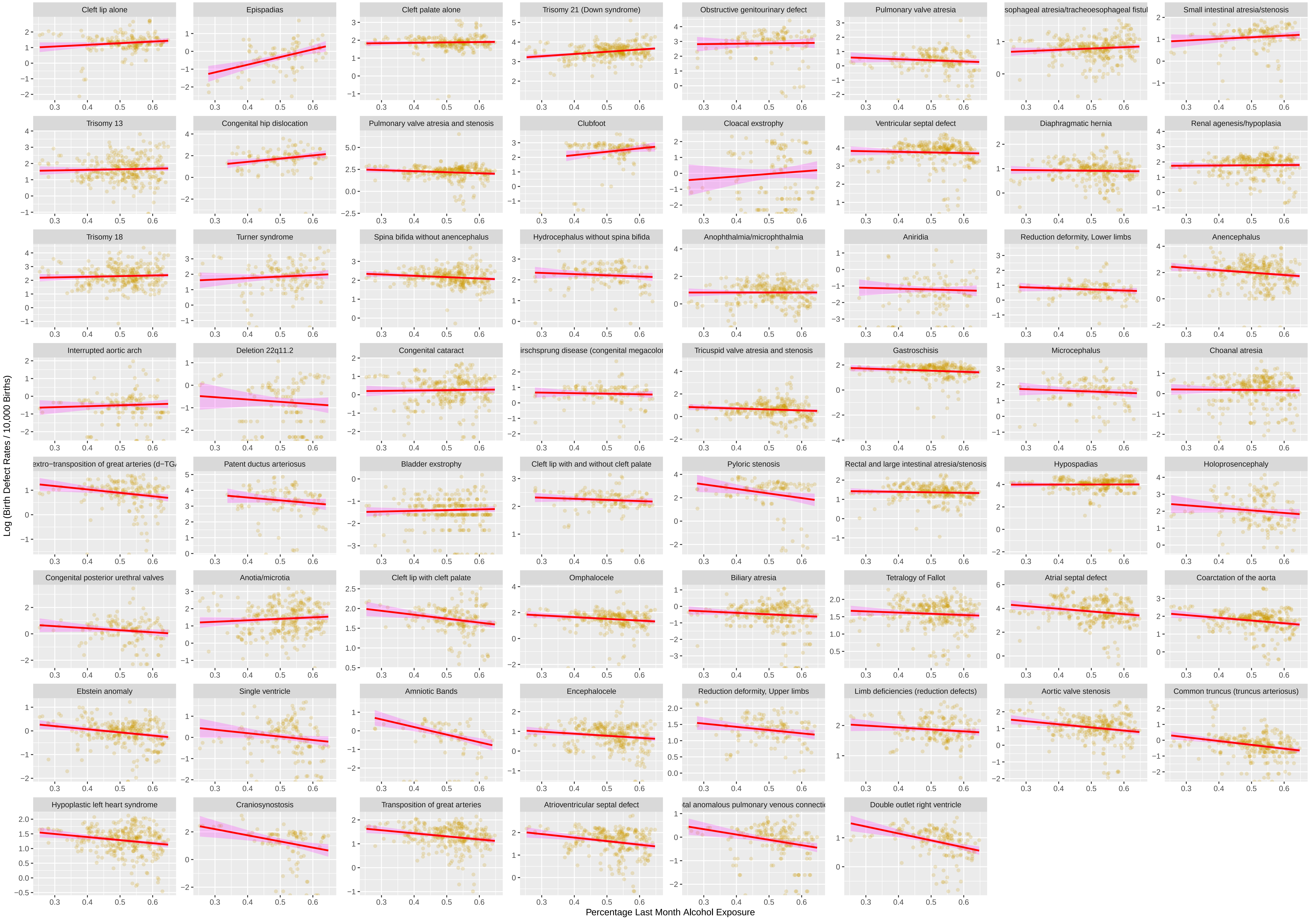

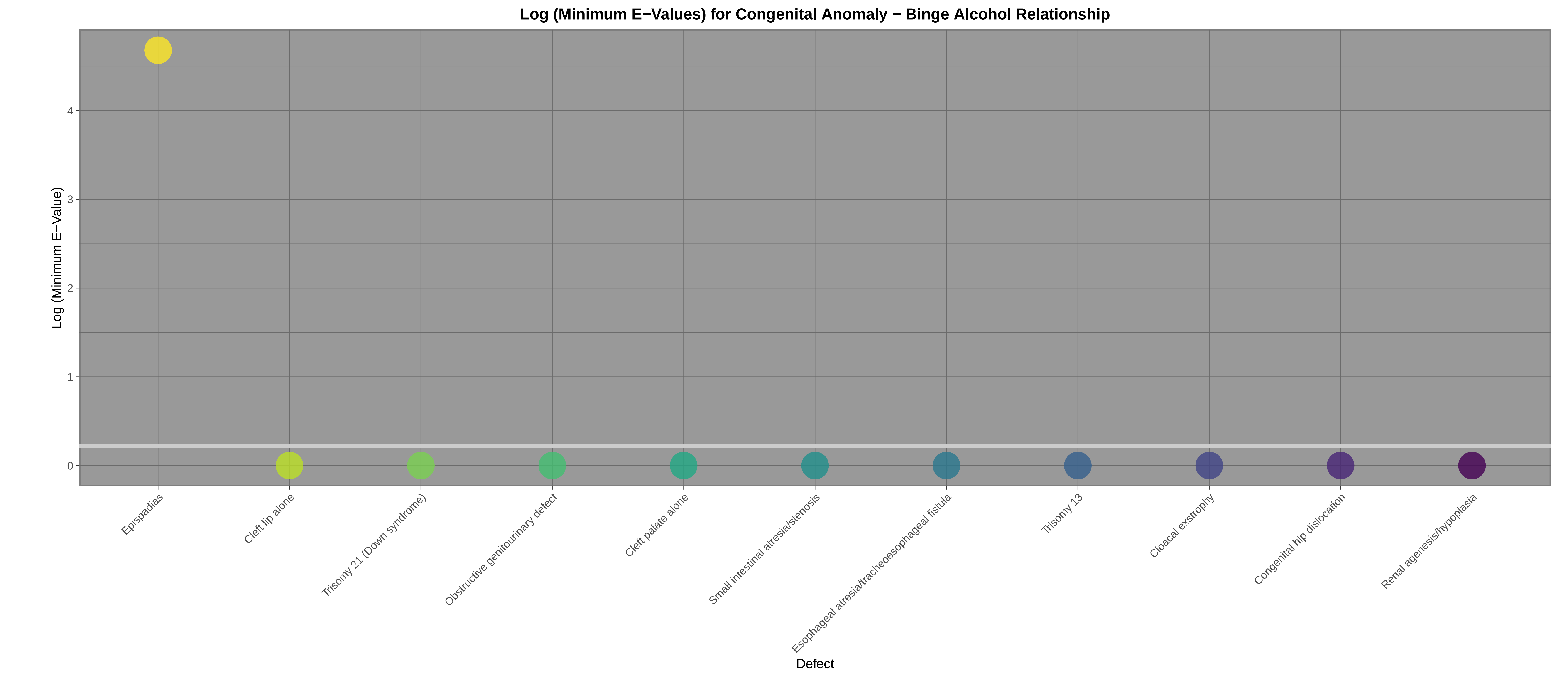

Log (Minimum E−Values) for Congenital Anomaly − Binge Alcohol Relationship
Log (Minimum E−Value)
4
3
2
1
0
Epispadias
Cleft lip alone
Trisomy 21 (Down syndrome)
Obstructive genitourinary defect
Cleft palate alone
Small intestinal atresia/stenosis
Esophageal atresia/tracheoesophageal fistula
Trisomy 13
Cloacal exstrophy
Congenital hip dislocation
Renal agenesis/hypoplasia
Defect

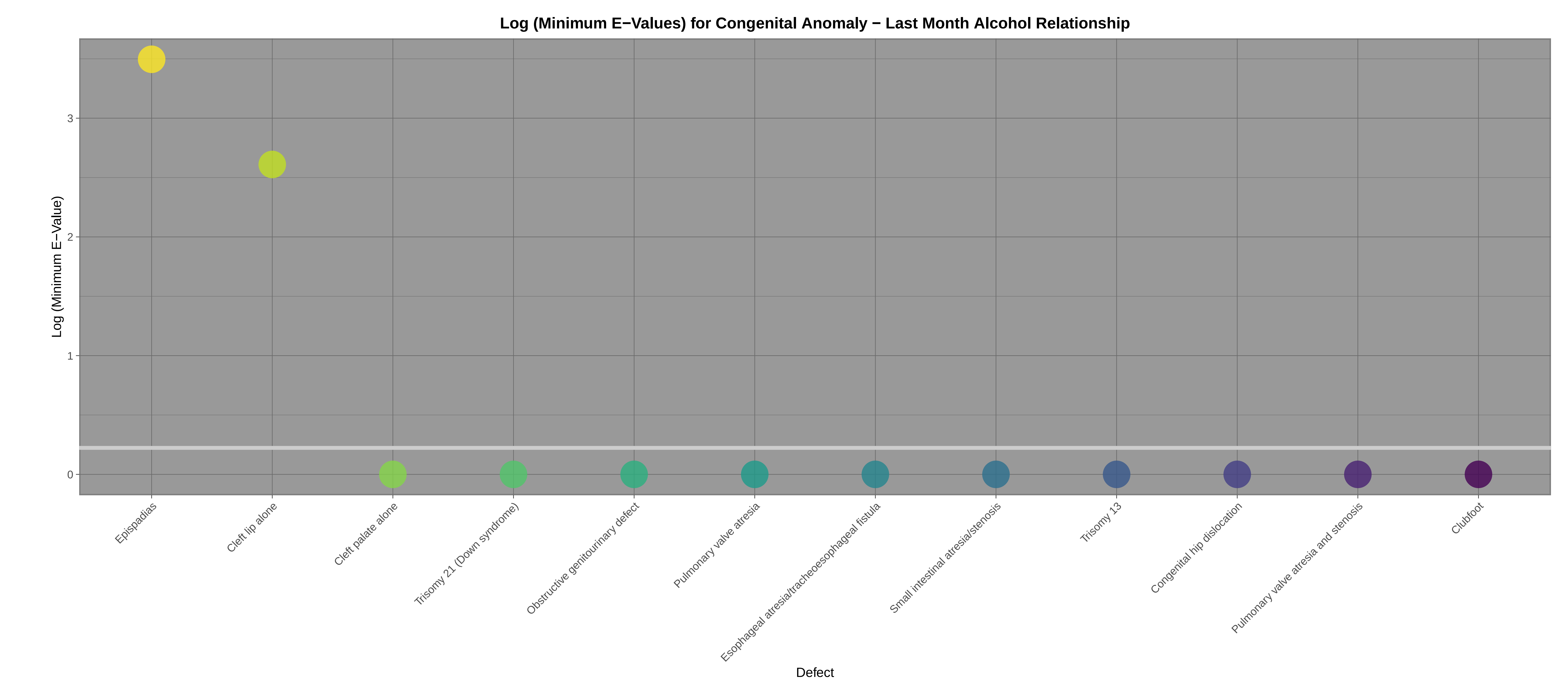
Log (Minimum E−Values) for Congenital Anomaly − Last Month Alcohol Relationship
Log (Minimum E−Value)
0
1
2
3
Epispadias
Cleft lip alone
Cleft palate alone
Trisomy 21 (Down syndrome)
Obstructive genitourinary defect
Pulmonary valve atresia
Esophageal atresia/tracheoesophageal fistula
Small intestinal atresia/stenosis
Trisomy 13
Congenital hip dislocation
Pulmonary valve atresia and stenosis
Clubfoot
Defect

Boxplot of Congential Anomaly Rates by Highest v Lowest Binge Alcohol Exposure Quintiles

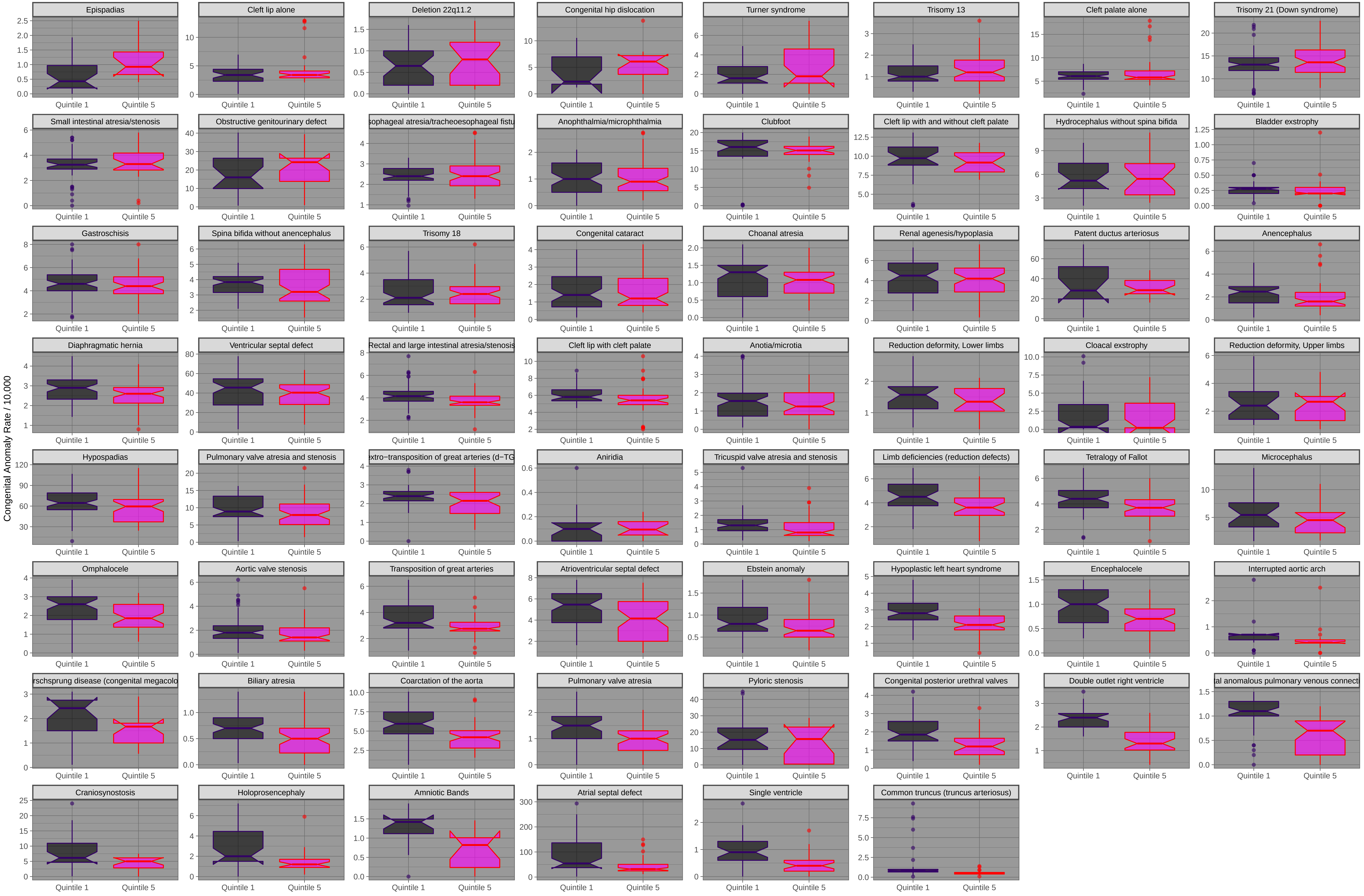

Boxplot of Congential Anomaly Rates by Highest v Lowest Monthly Alcohol Exposure Quintiles

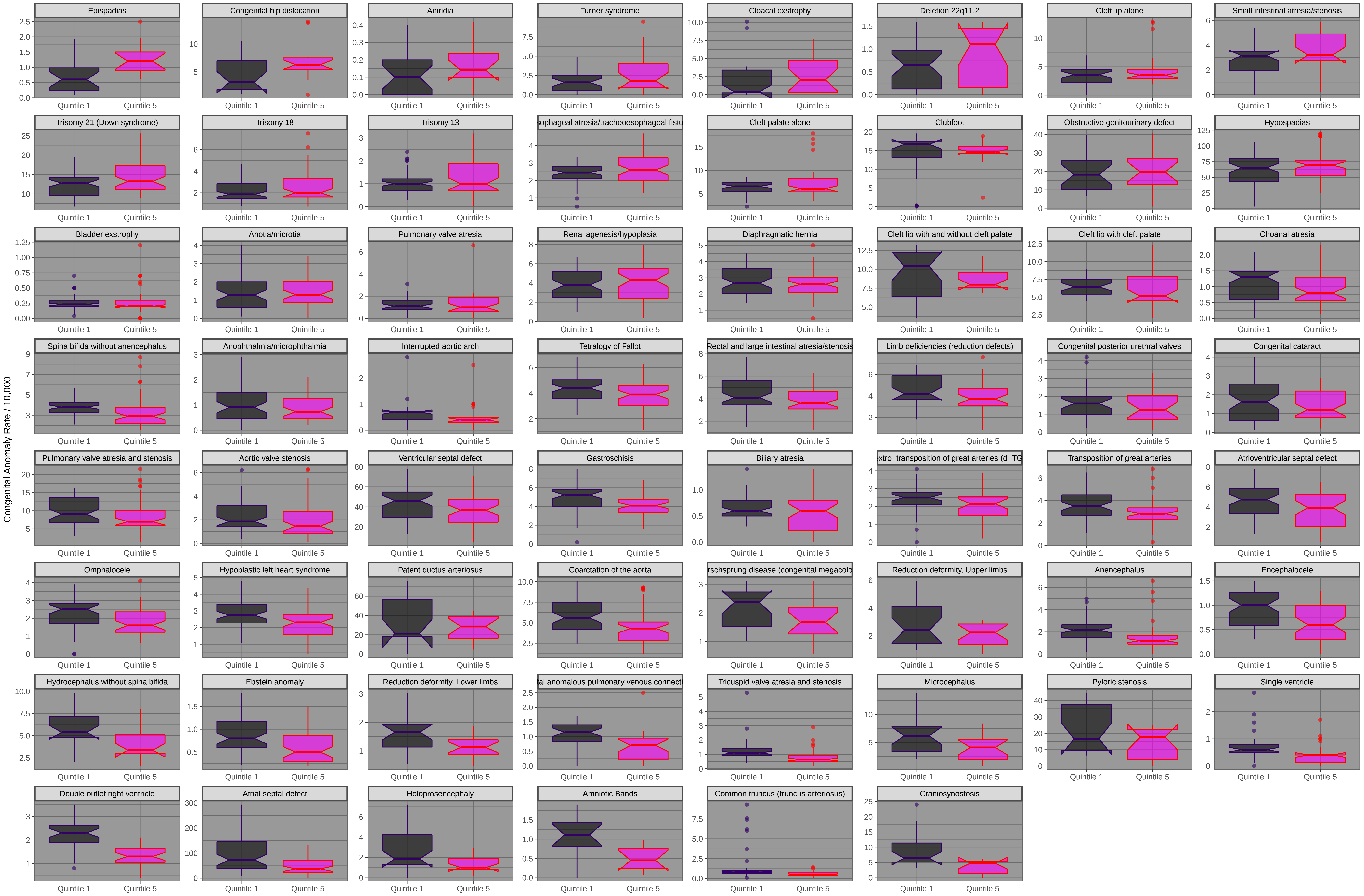

Boxplot of Congential Anomaly Rates by Highest v Lowest Analgesic Exposure Quintiles

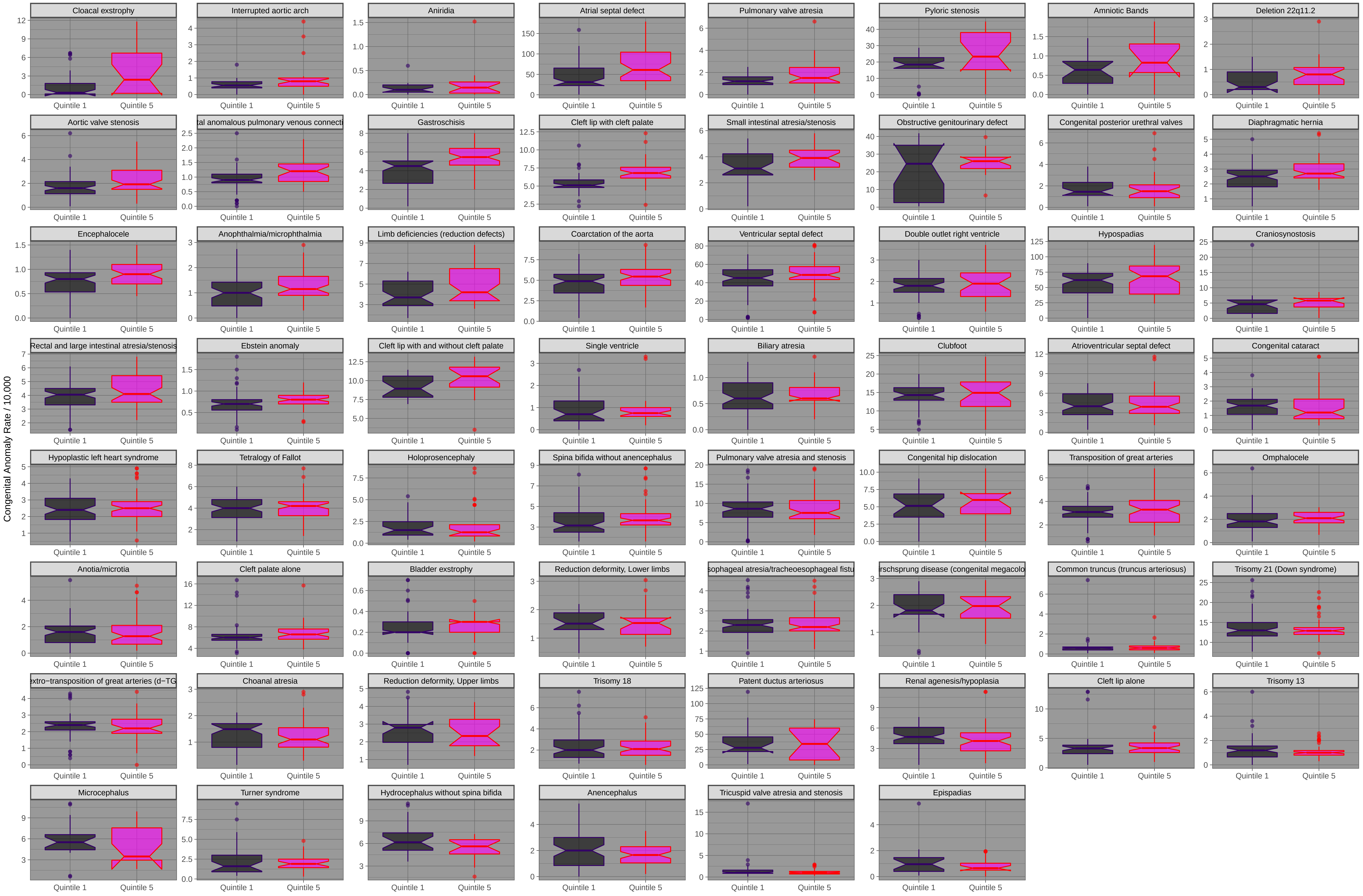

Boxplot of Congential Anomaly Rates by Highest v Lowest Cocaine Exposure Quintiles

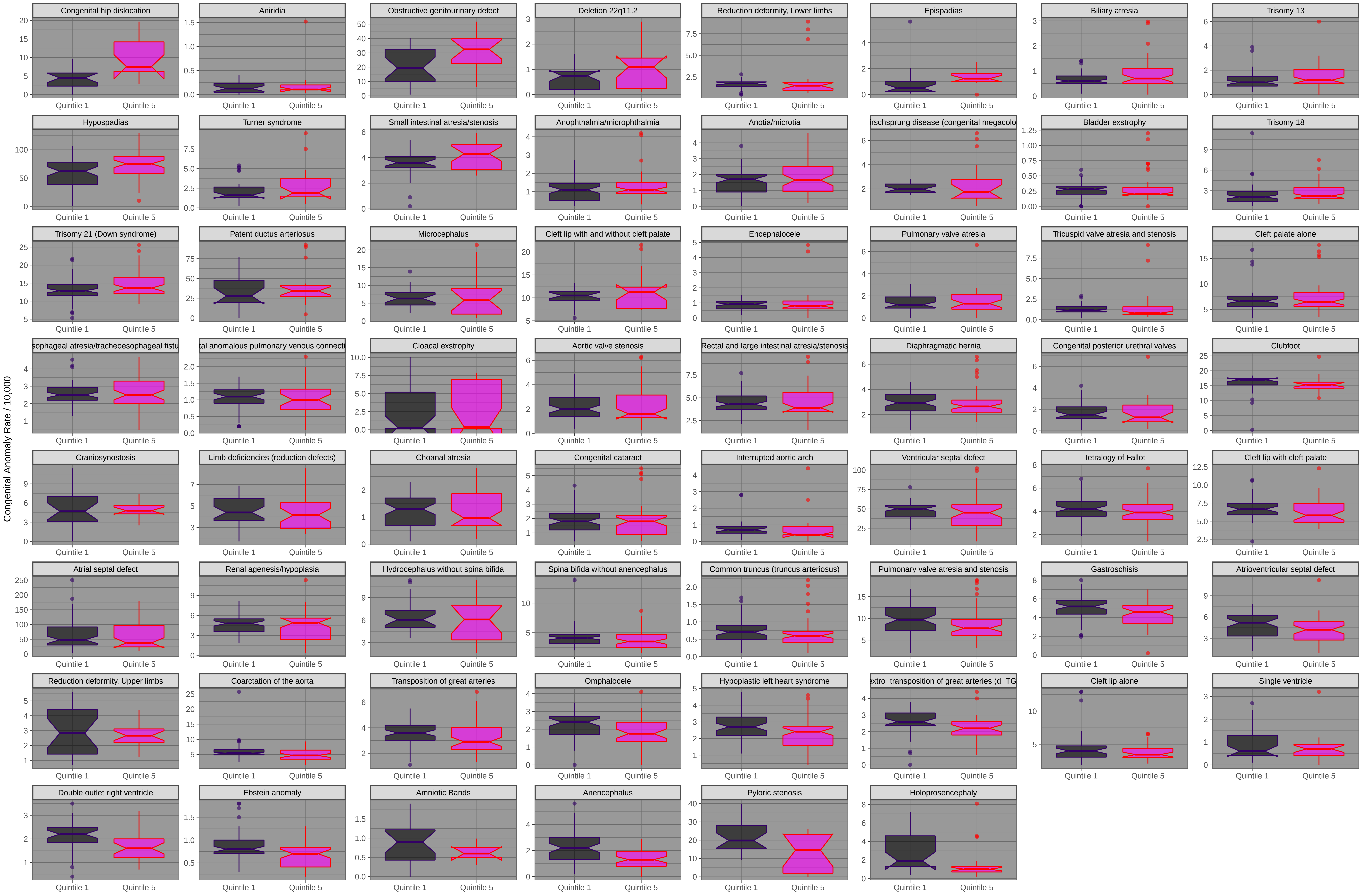

Additional Links to US State Neighbourhood Linkage Network for Small intestinal atresia/stenosis

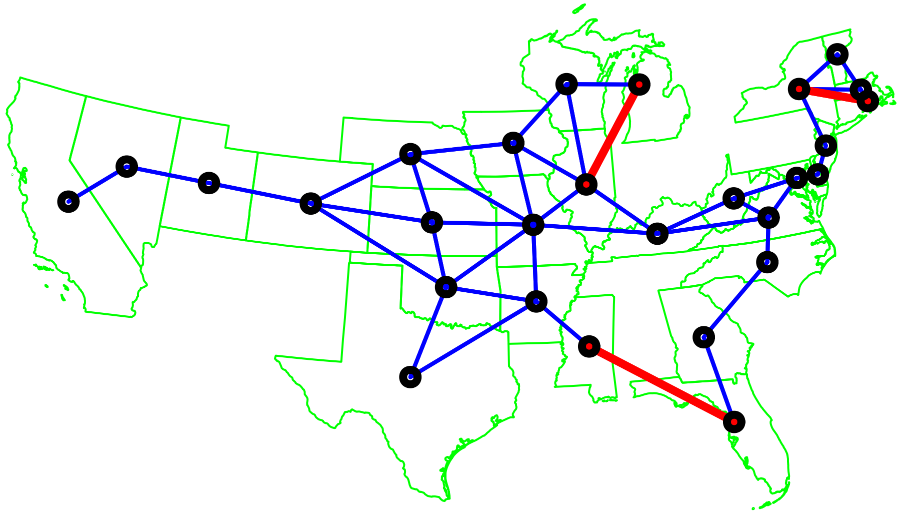

Final US State Neighbourhood Linkage Network for Small Intestinal Atresia / Stenosis

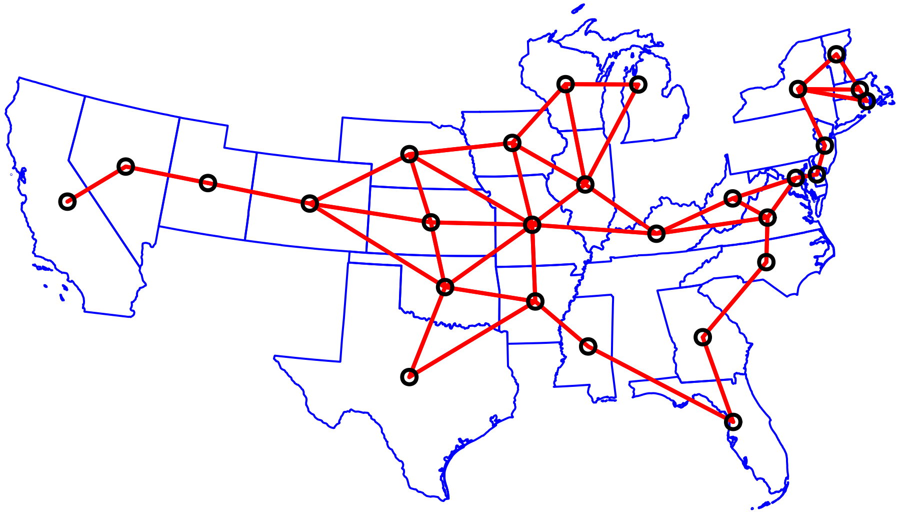

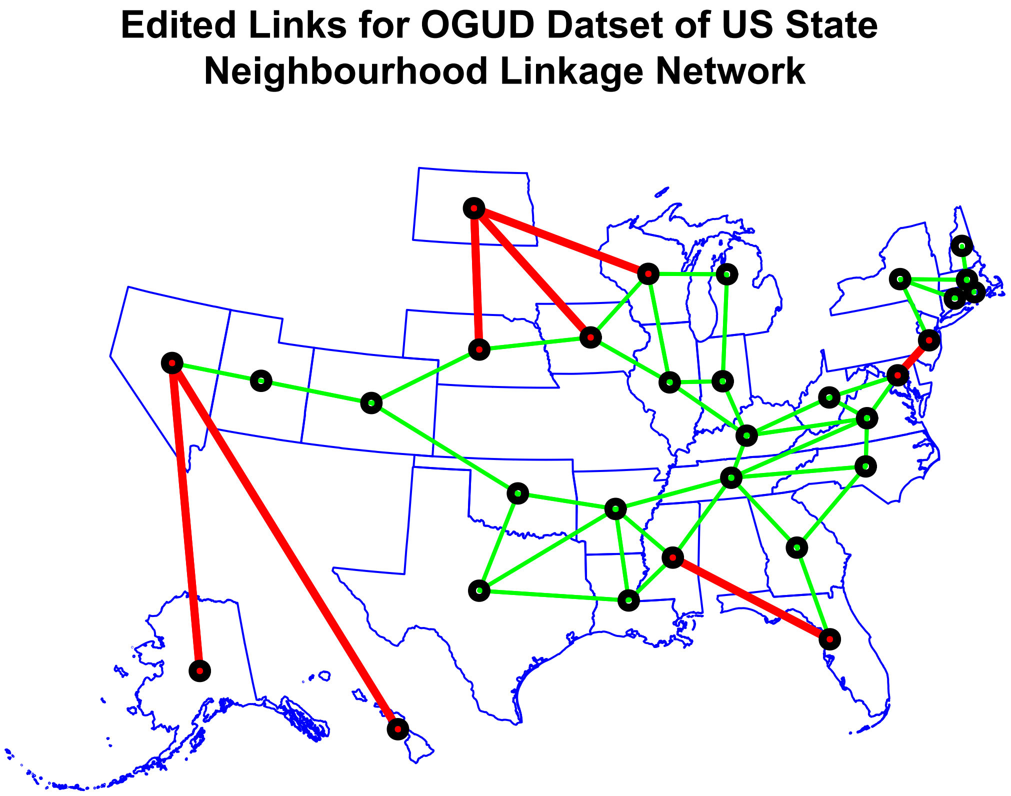
Edited Links for OGUD Datset of US State
Neighbourhood Linkage Network

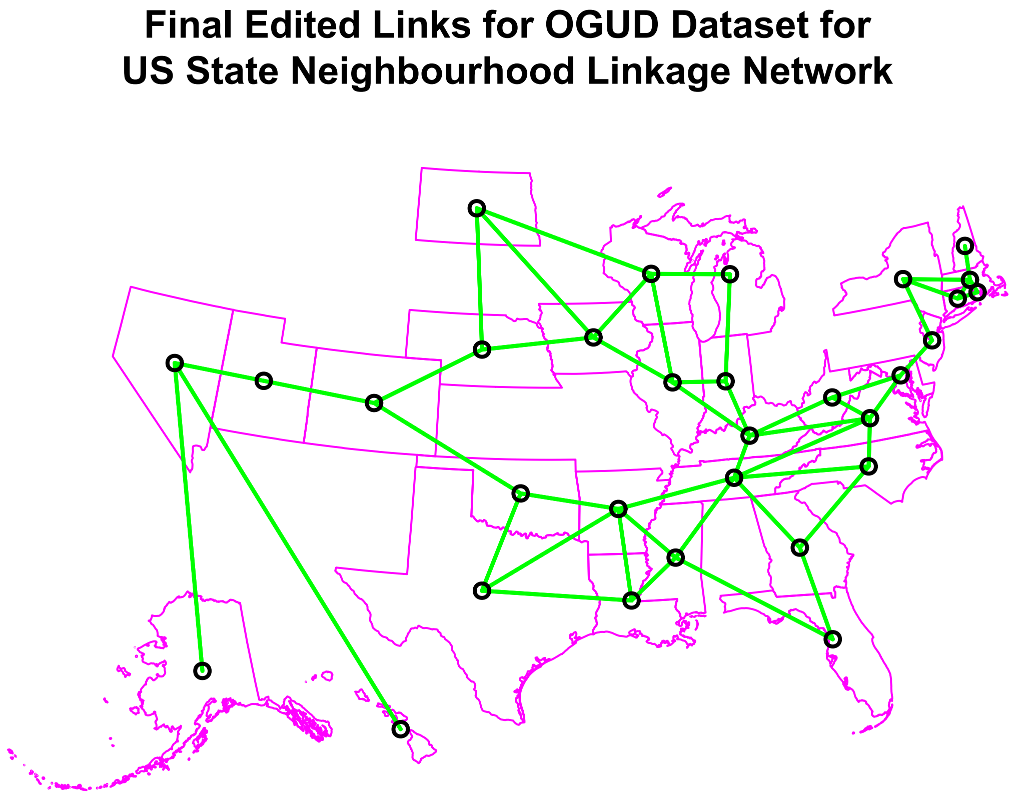
Final Edited Links for OGUD Dataset for
US State Neighbourhood Linkage Network
