# Supplementary material for: Geotemporospatial and causal inference epidemiological analysis of US survey and overview of cannabis, cannabidiol and cannabinoid genotoxicity in relation to congenital anomalies 2001–2015
Source: BMC Pediatr. 2022 Jan 19;22:47. doi: 10.1186/s12887-021-02996-3 (PMC8767720; doi:10.1186/s12887-021-02996-3)
Supplement: Supplementary file 3 — Additional file 3. [file 12887_2021_2996_MOESM3_ESM.docx]

Statistical Methods Supplementary Material

Statistical Analysis. Data was processed in R-Studio version 1.3.1093 (2009-2020) based upon R version 4.0.3 (2020-10-10). The decision to log transform covariates was guided by the results of the Shapiro-Wilks test. Data manipulation was with the “dplyr” package from the “tidyverse” suite [85]. Graphs were drawn using tidyverse “ggplot2” and maps were drawn in R-Base, “sf” (simple features) [86] and with ggplot2 [85, 87]. Colour palettes used both the plasma and viridis palettes from R-package “viridis” [88] and original specially created custom colour palettes. Bivariate maps were drawn with the two way colour matrices from R package “colorplaner”[89]. All maps and graphs were drawn specially for this report and are thus original. A new version of package “epiR” was specially developed for this project to allow the large integers involved to be processed. epiR is developed by Mark Stevenson and his colleagues [90]. Professor Stevenson was extremely kind and developed two new versions so that the present work could proceed. We were therefore able to utilize version epiR 2.0.11 in this work. epiR was used to calculate prevalence ratios, attributable fraction in the exposed, population attributable risks and significance levels along with their confidence intervals. R package “mgcv” was used to compute general additive models (GAM). Model comparison was with the Anova test from R-base.

Regression models. Linear trends were computed directly using linear regression from R-Base. The R-package “nlme” [91] using state as the random effect was used for repeated measures mixed effects regression. The R “survey” [92] package was used to conduct robust generalized linear regression and state was again used as the identity variable. The R-package “plm” was used to conduct panel regression with a space-time method [93]. For all regression formats model reduction was practised by the canonical method of the sequential manual deletion of the least signifcant term.

Geotemporospatial regression was conducted in the “splm” (spatial panel linear modelling) package [94] using the spreml (spatial panel random effects maximum likelihood) function. The R-package “spdep” [95] was used to compute spatial weights matrices describing the spatial relationship between states which was defined as edge and corner (“queen”) relationships and edited as described. Geospatial model specification was by the reverse method as described [96]. In full spatial panel random error maximum likelihood

(spreml) models four spatial coefficients are calculated as phi, psi, rho and lambda for the random effects, serial autocorrelation effects, spatial coefficient and autocorrelation of the spatial coefficients respectively [97]. In reverse model specification one deletes from the full model (error = “semsrre” + lag) those model error terms which are not significant [96]. This was the method used herein. Such procedures allow for fine control of the formal treatment of the model error terms.

Different forms of regression were employed for the following reasons. Mixed effects models have the advantage that repeated measurements can properly be considered from the same region. Inverse probability weighting is not possible in spatial models but can be performed in mixed effects, robust and panel models. Panel, mixed effects and spatial models allow the calculation of model standard deviations so E-Values can be computed from such models. Lagging cannot be used in mixed effects or robust models but can be applied in panel and spatial panel models. Instrumental variables can be employed in panel models but are not yet implemented for spatial panel models. In addition to allowing for formal consideration of spatial and temporal factors spatial panel models allow the use of both spatially and temporally lagged variables as well as spatially and temporally lagged variables considered simultaneously. It was therefore felt that by using several different types of regression the major results could be verified by several alternative methods.

Simultaneous multiple linear model analysis was conducted using the package “purrr” from the tidyverse [85] and tidy and glance from package “broom” [98] using established nest-map-unnest workflows. This recently developed and powerful technique allows the analysis of a whole long dataset providing data on all defects to be conducted by linear modelling in a single analysis run.

Causal inference. Two powerful techniques of formal causal inference were employed. Firstly inverse probability weighting (IPW) was included in all robust, mixed effects and panel models which had the effect of equilibrating exposure across all observed groups. This has the effect of pseudo-randomizing various exposures and allowing causal inferences to properly be drawn. The R-package “ipw” [99] was used to calculate inverse probability weights. Secondly the R-package “EValue” was used to compute E-values from both count data and from regression model outputs using the parameter estimate, its standard error and the standard model deviation [67, 68, 100]. E-Values were computed for regression models and for the predicted output from fitted models [67, 69, 101]. E-Values were calculated for panel, mixed effects and spatial panel models. It is noted in the literature that E-Values above 1.25 are indicative of causal relationships [67].

Predictive Spatial Modelling. Selected spatial panel models were chosen for predictive analysis as discussed in the text. Included in spatial panel (spreml) model objects is a vector of model predicted values ($fitted.values). Matrix multiplication was used to multiply 101 vectors, comprising percentiles zero to 100 of exposure to cannabidiol by the model parameter coefficients to produce a vector of model predicted values. Other terms were set at their mean value and the coefficient for the intercept was one. In each case the resulting predictions were outside and below the range of the NBDPN reported defect incidence, which was unsurprising as the models themselves included both log and lag terms.

The z-transformation is often used in statistics to adjust variable distributions and facilitate comparison between variables. Subtracting the mean of a data series from the values and dividing by the standard deviation of that dataset will transform it to have a mean of zero and a standard deviation of 1. This is the z-transformation which is widely used in statistical analysis. In the present case an extended z-transformation procedure was performed whereby the mean of the data series for the anomaly rate was added to the mean after z-transformation and the new standard deviation was set at the ratio of the median of the raw data series to the median of the fitted values from the model under consideration. The final scaling conversion formula may therefore be represented as follows:

*Recalibrated_Result =*

*((Res-mean(Res)) / ((sd(Res))/ (sd(FVV) * (median(SPDSST$DefxRt) / median(FVV))))) + (mean(SPDSST$DefxRt))*

where Res is the raw results from matrix multiplication, mean is the average, sd is the standard deviation, median is the median, SPDSST is the spatial panel space-time dataset for the congenital anomaly concerned, FVV is the fitted values from the spatial panel model, DefxRt is the observed rate for the congenital anomaly under consideration as reported by NBDPN, and $ is a placeholder for the dataframe indicating the variable name. The analysis of the model predictions which are reported were performed on the Recalibrated Results after application of the extended z-transformation conversion formula noted above.

P<0.05 was considered significant throughout.
